# Somatic CAG repeat expansion in blood associates with biomarkers of neurodegeneration in Huntington’s disease decades before clinical motor diagnosis

**DOI:** 10.1038/s41591-024-03424-6

**Published:** 2025-01-17

**Authors:** Rachael I. Scahill, Mena Farag, Michael J. Murphy, Nicola Z. Hobbs, Michela Leocadi, Christelle Langley, Harry Knights, Marc Ciosi, Kate Fayer, Mitsuko Nakajima, Olivia Thackeray, Johan Gobom, John Rönnholm, Sophia Weiner, Yara R. Hassan, Nehaa K. P. Ponraj, Carlos Estevez-Fraga, Christopher S. Parker, Ian B. Malone, Harpreet Hyare, Jeffrey D. Long, Amanda Heslegrave, Cristina Sampaio, Hui Zhang, Trevor W. Robbins, Henrik Zetterberg, Edward J. Wild, Geraint Rees, James B. Rowe, Barbara J. Sahakian, Darren G. Monckton, Douglas R. Langbehn, Sarah J. Tabrizi

**Affiliations:** 1 Huntington’s Disease Centre, Department of Neurodegenerative Disease, UCL Queen Square Institute of Neurology, University College London, London, UK; 2 Department of Psychiatry, University of Cambridge, Cambridge, UK; 3 School of Molecular Biosciences, College of Medical, Veterinary and Life Sciences, University of Glasgow, Glasgow, UK; 4 Department of Psychiatry and Neurochemistry, Institute of Neuroscience and Physiology, Sahlgrenska Academy at University of Gothenburg, Mölndal, Sweden; 5 Clinical Neurochemistry Laboratory, Sahlgrenska University Hospital, Mölndal, Sweden; 6 Department of Computer Science and Centre for Medical Image Computing, University College London, London, UK; 7 Dementia Research Centre, Department of Neurodegenerative Disease, UCL Queen Square Institute of Neurology, University College London, London, UK; 8 Department of Brain Repair and Rehabilitation, UCL Queen Square Institute of Neurology, University College London, London, UK; 9 Department of Psychiatry and Biostatistics, Carver College of Medicine and College of Public Health, University of Iowa, Iowa City, Iowa USA; 10 Dementia Research Institute, University College London, London, UK; 11 Department of Neurodegenerative Disease, UCL Queen Square Institute of Neurology, University College London, London, UK; 12 Faculdade Medicina da Universidade de Lisboa (FMUL), Lisbon, Portugal; 13 CHDI Management, Inc. Advisors to CHDI Foundation, Princeton, NJ USA; 14 Department of Psychology, University of Cambridge, Cambridge, UK; 15 Hong Kong Center for Neurodegeneassociated with substantially fasterrative Diseases, Clear Water Bay, Hong Kong, China; 16 Wisconsin Alzheimer’s Disease Research Center, University of Wisconsin School of Medicine and Public Health, University of Wisconsin—Madison, Madison, WI USA; 17 UCL Institute of Cognitive Neuroscience, University College London, London, UK; 18 Department of Clinical Neurosciences, University of Cambridge, Cambridge Biomedical Campus, Cambridge, UK; 19 Cambridge University Hospitals NHS Foundation Trust, Cambridge, UK; 20 Medical Research Council Cognition and Brain Sciences Unit, University of Cambridge, Cambridge, UK

**Keywords:** Huntington's disease, Prognostic markers, Disease genetics

## Abstract

Huntington’s disease (HD) is an autosomal dominant neurodegenerative disease with the age at which characteristic symptoms manifest strongly influenced by inherited *HTT* CAG length. Somatic CAG expansion occurs throughout life and understanding the impact of somatic expansion on neurodegeneration is key to developing therapeutic targets. In 57 HD gene expanded (HDGE) individuals, ~23 years before their predicted clinical motor diagnosis, no significant decline in clinical, cognitive or neuropsychiatric function was observed over 4.5 years compared with 46 controls (false discovery rate (FDR) > 0.3). However, cerebrospinal fluid (CSF) markers showed very early signs of neurodegeneration in HDGE with elevated neurofilament light (NfL) protein, an indicator of neuroaxonal damage (FDR = 3.2 × 10^−12^), and reduced proenkephalin (PENK), a surrogate marker for the state of striatal medium spiny neurons (FDR = 2.6 × 10^−3^), accompanied by brain atrophy, predominantly in the caudate (FDR = 5.5 × 10^−10^) and putamen (FDR = 1.2 × 10^−9^). Longitudinal increase in somatic CAG repeat expansion ratio (SER) in blood was a significant predictor of subsequent caudate (FDR = 0.072) and putamen (FDR = 0.148) atrophy. Atypical loss of interruption *HTT* repeat structures, known to predict earlier age at clinical motor diagnosis, was associated with substantially faster caudate and putamen atrophy. We provide evidence in living humans that the influence of CAG length on HD neuropathology is mediated by somatic CAG repeat expansion. These critical mechanistic insights into the earliest neurodegenerative changes will inform the design of preventative clinical trials aimed at modulating somatic expansion. ClinicalTrials.gov registration: NCT06391619.

## Abstract (web summary)

A comprehensive longitudinal analysis of individuals with preclinical Huntington’s disease identifies biomarkers of neurodegeneration and somatic expansion in blood DNA, detectable years before symptom onset.

## Main

Huntington’s disease (HD) is a devastating condition characterized by loss of striatal medium spiny neurons (MSNs) and striatal neurodegeneration^1^ leading to impaired motor, cognitive and neuropsychiatric function which typically manifests in middle age, with clinical diagnosis defined by the appearance of unequivocal HD-related motor signs. There are currently no disease-modifying treatments^2^.

HD is an autosomal dominant disorder and is caused by an expanded CAG repeat ≥40 in the huntingtin gene (*HTT*) coding for polyglutamine in the mutant huntingtin protein (mHTT), which is the presumed toxic entity leading to neuronal dysfunction and death. It is well established that inherited CAG repeat length has a strong influence on age at clinical motor diagnosis^3^. Notably, the *HTT* repeat is somatically unstable^4^ and expansion of tens or even hundreds of repeats are observed in the most vulnerable striatal neurons^5–8^; greater somatic expansion occurs with longer initial CAG length. Evidence indicating that faster individual-specific rates of somatic expansion in brain are associated with earlier clinical motor diagnosis and faster disease progression^9^ strongly suggests that somatic expansion is a key mechanism explaining the CAG effect on disease progression. Indeed, it has been suggested that somatic expansion is required to generate pathology, and that HD involves two thresholds as follows: first, the inherited CAG length that leads to further somatic expansion, and second, the intracellular pathogenic threshold above which neuronal dysfunction and death occur^10–13^. Consistent with this, a recent postmortem study suggests that neurons may experience decades of ‘biologically quiet’ somatic CAG repeat expansion with neuronal damage triggered by a cascade of repeat-length dependent transcriptional dysregulation events only when the CAG reaches a threshold of ~150 repeats^8^. Further understanding the dynamics of somatic expansion directly in the brain is hampered by the nonavailability of brain biopsy material from young living participants. Although somatic CAG expansion is clearly cell-type dependent^6–8^, faster individual-specific rates of somatic expansion in blood DNA are also associated with earlier clinical motor diagnosis^14^, suggesting that individual-specific somatic expansion rates in blood DNA are at least partially predictive of individual-specific somatic expansion rates in the brain. This hypothesis is supported by genetic modifier studies that reveal a panoply of DNA repair gene variants as modifiers of both *HTT* somatic expansion and HD clinical phenotypes^13–17^.

The polyglutamine-encoding CAG repeat tract in *HTT* is followed just downstream with a polymorphic polyproline-encoding CCG repeat. Typically, the intervening sequence between the CAG and CCG repeat tracts is comprised of a glutamine-encoding CAACAG cassette and a proline-encoding CCGCCA cassette. However, a number of atypical *HTT* repeat structures have been identified with loss of either or both of the intervening CAACAG or CCGCCA cassettes associated with an earlier age at clinical motor diagnosis; conversely, duplication of the CAACAG cassette delays this milestone^13,14,17–19^. These data reveal that both HD age at clinical motor diagnosis and the somatic expansion potential of the repeat are best predicted by pure CAG repeat length, rather than encoded polyglutamine length, providing additional support for a key role for somatic expansion in driving disease onset^13,14,18^.

The monogenic nature of HD and the existence of diagnostic and predictive testing for at-risk family members makes it a tractable disease and much progress has been made towards developing disease modification treatments^2^. The first phase 1/2 trial of an antisense oligonucleotide (ASO), tominersen, showed dose-dependent lowering of mutant huntingtin levels^20^. Although the subsequent phase 3 trial was halted early due to adverse safety concerns^21^, a phase 2 study to better establish safety and tolerability earlier in disease progression is ongoing (ClinicalTrials.gov registration: NCT05686551). Alternative approaches such as allele-specific huntingtin-lowering, protein splicing modulation, and gene therapy are also currently being trialed (reviewed in ref. ^22^). Additionally, somatic expansion and proteins, such as MSH3 and FAN1, are now being actively pursued as therapeutic targets in HD. A key question in using such therapies will be determining the optimal timing for treatment. The appearance of HD motor signs is already accompanied by substantial striatal neurodegeneration, and earlier treatment seems likely to produce greater clinical benefit. However, all the studies to date have relied on postmortem brain analyses to model the link between CAG repeat expansion to the earliest pathological progression of the disease. Understanding the triggers of the neurodegenerative process is vital in the search for future therapies and identifying the best time to treat to provide therapeutic intervention.

The greatest opportunity to influence disease progression lies in early treatment, with the goal of delaying or preventing clinical motor diagnosis. Numerous large observational studies show that brain changes occur decades from predicted clinical motor diagnosis^23–25^ and that subtle cognitive and motor signs emerge as HD gene expanded (HDGE) individuals approach clinical motor diagnosis. The recent introduction of the HD Integrated Staging System (HD-ISS) provides a new empirical framework for classifying people with HD throughout life^26^, with stage 0 being the HDGE group with striatal volumes within the general population range, stage 1 being the presence of a biomarker of pathogenesis (caudate and/or putamen volume change), stage 2 being the presence of motor and/or cognitive signs and stage 3 being marked by the onset of functional impairment^26^. Cohorts in the earliest stages will likely gain the most benefit from preventative therapies.

A key challenge in delivering preventative treatments is to identify and validate robust measures in HD-ISS stages 0 and 1, where the absence of outward signs of impairment renders established motor and cognitive testing batteries insensitive. HD Young Adult Study (HD-YAS) is a unique cohort, ~23 years from predicted clinical motor diagnosis at baseline with deep phenotyping including biofluid, imaging, clinical, cognitive and motor assessments. Our cross-sectional baseline data demonstrated subtle elevations in biofluid biomarkers, such as cerebrospinal fluid (CSF) neurofilament light (NfL), accompanied by slightly smaller putamen volumes in the HDGE group compared to unaffected controls^25^. Despite this, there was no difference in functional performance between the groups. This cohort, therefore, spans an optimum window for investigating the potential of interventions to delay or prevent symptoms.

Here we present 4.5-year follow-up data from HD-YAS, a deep-phenotyped longitudinal study of young stages 0 and 1 HDGE adults, ~19 years before clinical motor diagnosis. We hypothesized that the effects of somatic expansion in the brain might be detected long before clinical motor onset and tested this hypothesis through detailed longitudinal analysis of preclinical HD phenotypes, biomarkers of neurodegeneration and somatic expansion in blood DNA. We examined change over time in a range of assessments with the aim of identifying ongoing neuropathology and associations with somatic CAG expansion in blood DNA and *HTT* repeat structures, decades before predicted clinical motor diagnosis, and biomarkers of disease progression, which may have utility in future prevention trials.

## Results

### Participant characteristics

**Fig. 1:**
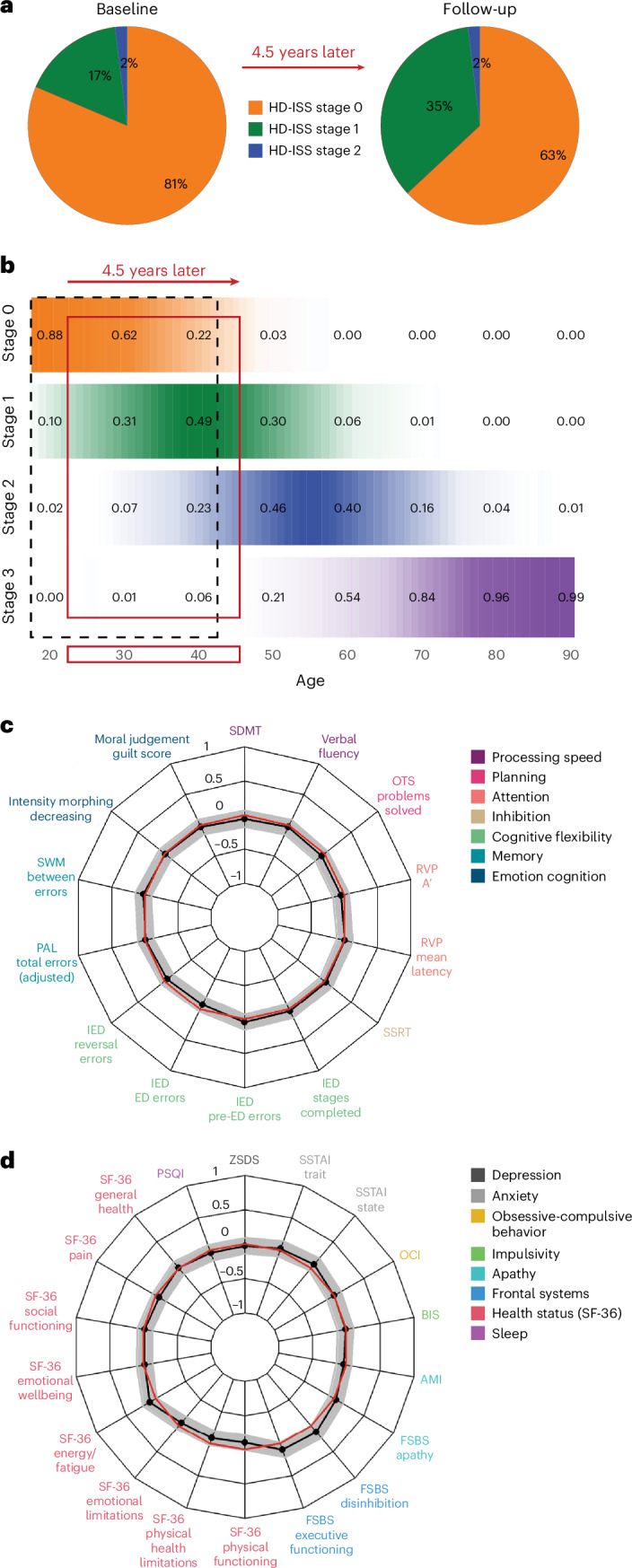
Longitudinal change in clinical, cognitive and neuropsychiatric measures. **a**, The distribution of HD-ISS stages at baseline and at follow-up 4.5 years later. **b**, A probability matrix for being in each HD-ISS stage across different ages for individuals with a mean CAG repeat length of 42, which is comparable to the HD-YAS cohort. These probabilities are derived from data in the Enroll-HD, PREDICT-HD and TRACK-HD studies, which were used to develop the HD-ISS^26^. The black dashed box highlights the HD-YAS cohort at baseline, while the red box indicates their position at follow-up after 4.5 years. **c**, A radar plot showing group differences in longitudinal changes in cognitive measures. **d**, A radar plot showing group differences in longitudinal changes for neuropsychiatric and functional measures. The black line represents the standardized mean difference between the HDGE and control groups, with conventional frequentist 95% CI shaded in gray. The red circle denotes no difference between means; values within this circle indicate greater change over time in the HDGE group. After FDR correction for multiple comparisons, there were no significant longitudinal group differences in any cognitive or neuropsychiatric measures. Further details on longitudinal changes in cognitive measures can be found in Supplementary Table 1 and neuropsychiatric measures in Supplementary Table 2. Cross-sectional changes in cognitive measures are presented in Supplementary Table 3 and neuropsychiatric changes in Supplementary Table 4. Cross-sectional findings are visualized in Supplementary Fig. 1. AMI, Apathy Motivation Index; BIS, Barratt Impulsivity Scale; CI, confidence interval; ED, extra dimensional; FSBS, Frontal Systems Behavioral Scale; IED, intra–extra-dimensional set shifting; OCI, Obsessive-Compulsive Inventory; OTS, One Touch Stockings; PAL, paired associates learning; PSQI, Pittsburgh Sleep Quality Index; RVP, rapid visual processing; RVP A’, a signal detection theory measure of target sensitivity and mean response latency; SDMT, Symbol Digit Modalities Test; SF-36, 36-item self-report survey; SSRT, stop-signal reaction time; SSTAI, Speilberger State-Trait Anxiety Inventory; SWM, spatial working memory; ZSDS, Zung Self-rating Depression Score.

**Extended Data Fig. 1:**
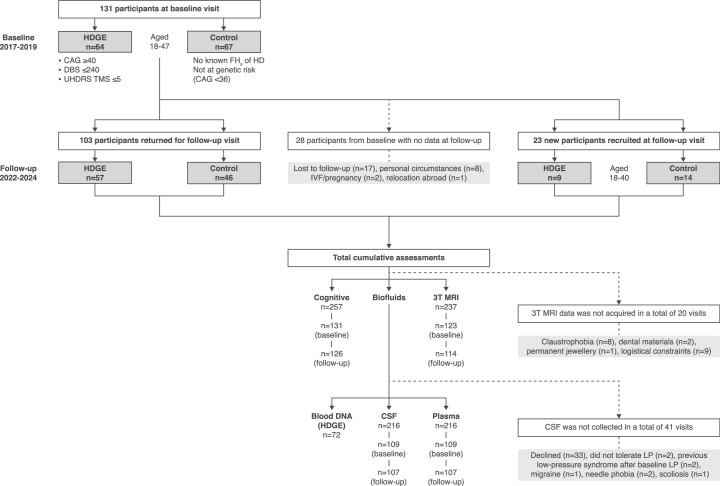
Flowchart of recruitment, follow-up and total cumulative assessments. Flowchart detailing participant enrollment at baseline (2017–2019) and the retention and recruitment of new participants at follow-up (2022–2024) in the Huntington’s Disease Young Adult Study (HD-YAS). CSF, cerebrospinal fluid; DBS, disease burden score; FH_x_, family history; HDGE, HD gene expanded; IVF, in vitro fertilization; LP, lumbar puncture; TMS, Total Motor Score; UHDRS, Unified Huntington’s Disease Rating Scale.

A total of 131 (64 HDGE and 67 controls) participants attended at baseline and 103 (57 HDGE and 46 controls) returned for follow-up ~4.5 years later (see Extended Data Fig. 1 for reasons for dropout). To account for those not returning, we recruited 23 new participants (9 HDGE and 14 controls) giving a total of 154 participants (73 HDGE and 81 controls). At baseline, 44 (81%) participants of the cohort were in HD-ISS stage 0, 9 (17%) in stage 1 and 1 (2%) in stage 2 (Fig. 1a). Over 4.5 years, 10 (~23%) participants moved from stage 0 to stage 1; there was no progression to stage 2. The transition in staging within the HD-YAS cohort is depicted by overlaying the probability matrix for each HD-ISS stage across different ages for individuals with a mean CAG repeat length of 42, comparable to the mean CAG repeat length of our cohort (Fig. 1b). Here we describe further longitudinal results from the participants; cross-sectional results, updated from the original baseline study, are provided in Supplementary Results and Discussion.

There were no significant differences (false discovery rate (FDR) < 0.15) between the HDGE and control groups in age, sex, interval between visits, education score or National Adult Reading Test (a measure of premorbid intelligence; Extended Data Table 1).

### Cognitive and neuropsychiatric assessments

There was no significant longitudinal disease-related decline in any of the comprehensive cognitive (FDR > 0.8; Fig. 1c) or neuropsychiatric (FDR > 0.3; Fig. 1d) assessments, demonstrating that change in the HDGE group was no different from matched controls. Cross-sectional results are shown in Supplementary Fig. 1 and summary statistics are provided in Supplementary Tables 1 and 2 for longitudinal and Supplementary Tables 3 and 4 for cross-sectional results.

### Neuroimaging

After quality control, longitudinal data were available for 88 (54 HDGE and 34 controls) participants for volumetric imaging, 83 (50 HDGE and 33 controls) for diffusion-weighted imaging (DWI) and 75 (43 HDGE and 32 controls) for multiparametric mapping (MPM). As left-handed participants were excluded, 70 (43 HDGE and 27 controls) participants were available for the structural connectivity analysis. See Supplementary Table 5 and Supplementary Methods for further details.

**Fig. 2:**
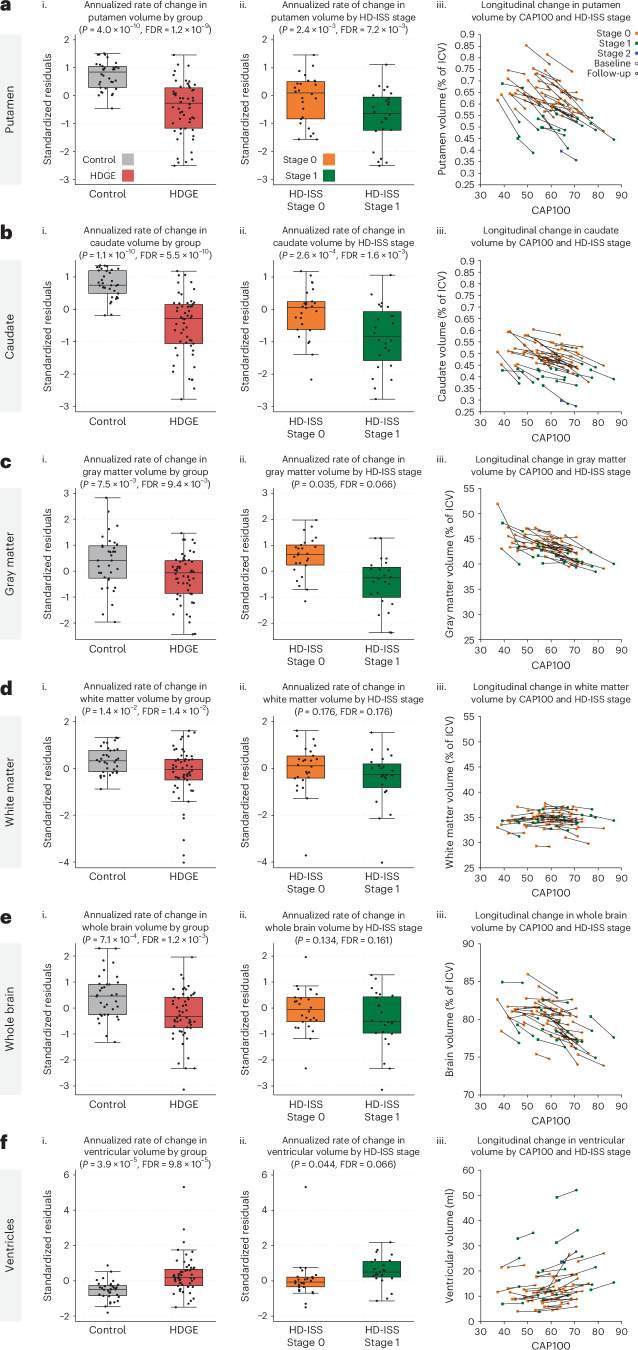
Annualized changes in volumetric measures longitudinally. **a**–**f**, Putamen (**a**), caudate (**b**), gray matter (**c**), white matter (**d**), whole brain (**e**) and ventricles (**f**) are shown. For each structure, we present (i) comparison of standardized residuals (age- and sex-adjusted) for the annualized rate of change in HDGE (*n* = 54; red) and control (*n* = 34; gray) groups, (ii) comparison of standardized residuals for annualized rate of change within HDGE by follow-up HD-ISS stage 0 (orange) and stage 1 (green) and (iii) scatterplots of volume by CAP100 score, colored by HD-ISS stage within HDGE. Repeated visits per participant are connected by black lines, with baseline shown as squares and follow-up as circles. HD-ISS stages are represented as follows: stage 0 (orange), stage 1 (green) and stage 2 (blue). Negative standardized residuals denote a rate of change below the adjusted mean across groups. Each box plot displays the median (horizontal line), interquartile range (box) and whiskers extending to 1.5× IQR. Sample sizes (*n*) reflect biological replicates per group, with *n* = 54 for HDGE and *n* = 34 for controls; data represent longitudinal measures per participant, with no technical replicates. Volumetric change analyses for brain structures, excluding the putamen, used a single boundary-shift integral measure or voxel-based morphometry measure of scan pairs per participant (baseline to follow-up) converted to annual rates and modeled by ordinary least squares regression. Putamen changes were calculated by subtracting baseline MALP-EM segmentations from follow-up segmentations and dividing the result by the follow-up duration. Analysis results and residual adjustments reflect control for baseline age, sex and their interaction. Statistical two-sided group comparisons were adjusted for multiple comparisons using the FDR, with *P* values, degrees of freedom and confidence limits provided in Extended Data Table 2. CAP, CAG-Age Product; ICV, intracranial volume; IQR, interquartile range.

The HDGE group showed significantly greater rates of atrophy in putamen (*P* = 4.0 × 10^−10^, FDR = 1.2 × 10^−9^) and caudate (*P* = 1.1 × 10^−10^, FDR = 5.5 × 10^−10^). There were also significant group differences for gray matter (*P* = 7.5 × 10^−3^, FDR = 9.4 × 10^−3^), white matter (*P* = 1.4 × 10^−2^, FDR = 1.4 × 10^−2^) and whole brain (*P* = 7.1 × 10^−4^, FDR = 1.2 × 10^−3^) with associated ventricular expansion (*P* = 3.9 × 10^−5^, FDR = 9.8 × 10^−5^; Fig. 2). Caudate, putamen and white matter loss were significantly predicted by age and CAG (*P* = 2.1 × 10^−7^, FDR = 1.0 × 10^−6^; *P* = 1.5 × I0^−8^, FDR = 8.9 × 10^−8^; *P* = 0.01, FDR = 0.012, respectively).

DWI demonstrated elevated rates of longitudinal change in all diffusion and neurite orientation and dispersion density imaging metrics across multiple regions of interest in the HDGE group compared to controls (FDR < 0.15). The splenium of the corpus callosum, the anterior capsule and the external capsule showed associations with age and CAG (FDR < 0.15). There were no significant between-group differences in the rate of change for any of the structural connectivity (all FDR > 0.4) or MPM measures (all FDR > 0.3), nor any evidence of an influence of age and CAG (all FDR > 0.15).

Neuroimaging results suggest that across HD-ISS stages 0 and 1, there are already elevated rates of brain atrophy accompanied by subtle microstructural white matter changes. See Extended Data Tables 2 and 3 for summary statistics for longitudinal volumetric and diffusion results, respectively. Summary statistics for remaining longitudinal metrics are provided in Supplementary Tables 6 and 7 and cross-sectional data in Supplementary Tables 8–11.

### Biofluids

A total of 216 biofluid samples were collected across baseline and follow-up visits over the 4.5-year interval. Paired fasting CSF and plasma samples were acquired in 86 (53 HDGE and 33 controls) of the 103 (83.5%) longitudinal participants.

**Fig. 3:**
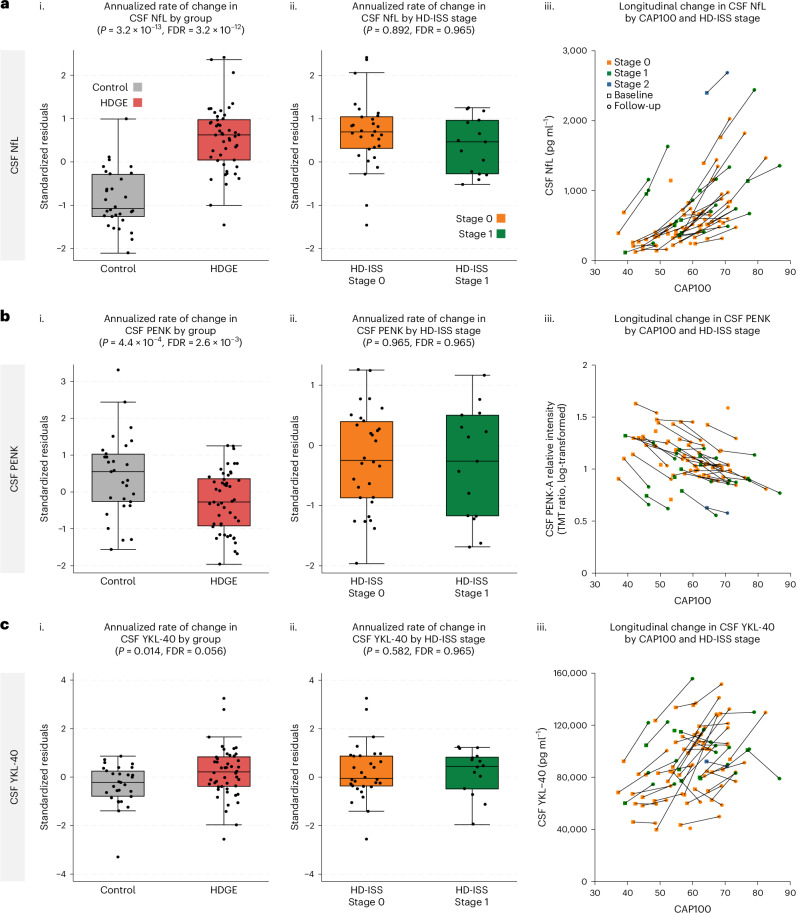
Annualized changes in biofluid markers longitudinally. **a**–**c**, CSF NfL (**a**), CSF PENK (**b**) and CSF YKL-40 (**c**) are shown. For each biofluid biomarker, we present (i) comparison of standardized residuals (age- and sex-adjusted) for the annualized rate of change in HDGE (*n* = 48; red) and control (*n* = 30; gray) groups, (ii) comparison of standardized residuals for annualized rate of change within HDGE by HD-ISS stage 0 (orange) and stage 1 (green) and (iii) scatterplots of biofluid marker levels by CAP100 score, colored by HD-ISS stage within HDGE. Repeated visits per participant are connected by black lines, with baseline shown as squares and follow-up as circles. HD-ISS stages are represented as follows: stage 0 (orange), stage 1 (green) and stage 2 (blue). Negative standardized residuals denote a rate of change below the adjusted mean across groups. Each box plot displays the median (horizontal line), interquartile range (box) and whiskers extending to 1.5× IQR. Sample sizes (*n*) reflect biological replicates per group, with *n* = 48 for HDGE and *n* = 30 for controls; data represent longitudinal measures per participant. All statistical analyses were conducted using mixed-effect linear models with a participant-specific random effect, controlling for age, sex and their interaction. Natural log-transformed concentrations served as the outcomes in these models. Statistical two-sided group comparisons were adjusted for multiple comparisons using the FDR, with *P* values, degrees of freedom and confidence limits provided in Supplementary Table 17. Please note one prominent outlier in the control group with marked NfL elevation, as previously reported at baseline^25^. This outlier showed no additional cause on further investigation, with normal T1 MRI brain scan and normal CSF white and red cell counts. Additionally, this control participant did not deviate from other biofluid or cognitive parameters and was, therefore, not excluded from the analysis.

**Extended Data Fig. 2:**
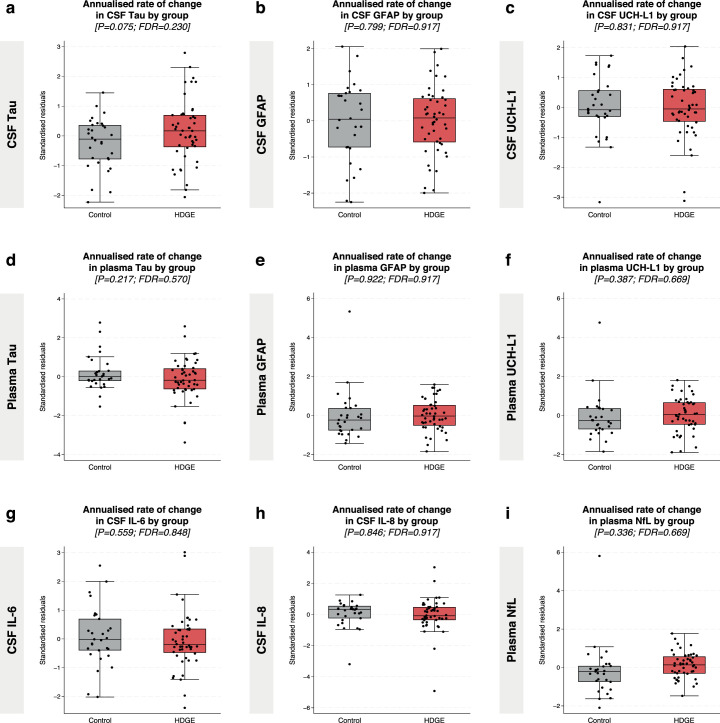
Annualized rate of change in non-significant biofluid markers. Panel shows comparison of standardized residuals for annualized rate of change in the HDGE vs. control groups for: (**a**) CSF Tau, (**b**) CSF GFAP, (**c**) CSF UCH-L1, (**d**) plasma Tau, (**e**) plasma GFAP, (**f**) plasma UCH-L1, (**g**) CSF IL-6, (**h**) CSF IL-8 and (**i**) plasma NfL. Negative standardized residuals indicate that the rate of change was less than the adjusted mean rate of change across both groups. The horizontal lines represent medians, boxes indicate the upper and lower quartiles, and whiskers are 1.5× IQR. All statistical analyses were conducted using mixed-effect linear models with a participant-specific random effect, controlling for age, sex and their interaction. Natural log-transformed concentrations served as the outcomes in these models. Statistical two-sided group comparisons and correlations controlled for the effects of age and sex and were corrected for multiple comparisons using the FDR. CSF, cerebrospinal fluid; FDR, false discovery rate; GFAP, glial fibrillary acidic protein; HDGE, HD gene expanded; IL, interleukin; IQR, interquartile range; NfL, neurofilament light; UCH-L1, ubiquitin carboxyl-terminal hydrolase L1; YKL-40, also known as chitinase-3 like-protein-1 (CHI3L1).

From a significantly increased baseline, CSF NfL (Fig. 3a) and CSF YKL-40 (also known as chitinase-3 like-protein-1 (CHI3L1)) (Fig. 3c) rose more rapidly in HDGE compared to controls (*P* = 3.2 × 10^−13^, FDR = 3.2 × 10^−12^ and *P* = 0.01, FDR = 0.056, respectively). New to this timepoint, proenkephalin (PENK), a surrogate marker for striatal MSN state, measured in CSF, showed a significant longitudinal reduction in HDGE individuals compared to controls (*P* = 4.4 × 10^−4^, FDR = 2.6 × 10^−3^; Fig. 3b). An increase in plasma NfL was nonsignificant (*P* = 0.336, FDR = 0.669; Extended Data Fig. 2).

Cross-sectionally, log concentrations of both CSF NfL (*P* = 5.4 × 10^−30^, FDR = 6.5 × 10^−29^) and PENK (*P* = 1.7 × 10^−7^, FDR = 1.0 × 10^−6^) were highly associated with age, CAG length and their interaction. There was also evidence for an influence on longitudinal change in CSF NfL (*P* = 0.027, FDR = 0.322) and PENK (*P* = 0.0547, FDR = 0.328). Plasma NfL had a similar cross-sectional association (*P* = 7.2 × 10^−7^, FDR = 2.9 × 10^−6^) but no significant longitudinal association with age and CAG. Regression coefficients are reported in Supplementary Tables 12–14.

Slightly higher annualized rates of change in NfL in CSF and plasma were observed in the HDGE group at stage 0 compared to stage 1 on follow-up but did not reach the threshold of significance (FDR > 0.15). Mean CSF NfL levels (across both visits) were higher in HD-ISS progressor (stages 0 to 1—mean = 6.89 pg ml^−1^, log scale) compared to nonprogressors (stage 0 to 0—mean = 6.11 pg ml^−1^, log scale; stage 1 to 1—mean = 6.37 pg ml^−1^, log scale; Supplementary Table 15). After adjusting for age, sex and their interaction, the difference between stage 0 to 1 progressors and stage 0 nonprogressors was statistically significant (*P* = 0.0004). Similarly, the difference between stage 0 to 1 progressors and stage 1 nonprogressors was significant (*P* = 0.045) when controlling for age and sex, but nonsignificant without these adjustments. No significant differences were observed for plasma NfL levels (Supplementary Table 16).

CSF mHTT levels were notably very low than later disease stages^27^, with only 38.3% (*n* = 41/107) of samples exceeding the lower limit of quantification and demonstrating an acceptable coefficient of variation below 30% (Supplementary Fig. 2).

The rate of change in other biofluid markers, including plasma NfL, CSF and plasma tau, CSF and plasma glial fibrillary acidic protein (GFAP), CSF and plasma ubiquitin carboxyl-terminal hydrolase L1 (UCH-L1), and CSF interleukin-6 (IL-6) and IL-8, showed no significant differences between groups (Extended Data Fig. 2). Additionally, none of the fluid biomarkers, including NfL, had an association with age, CAG or age-by-CAG interaction (FDR > 0.15). See Supplementary Table 17 for longitudinal and Supplementary Table 18 for cross-sectional summary statistics.

### Somatic expansion ratios in blood

**Fig. 4:**
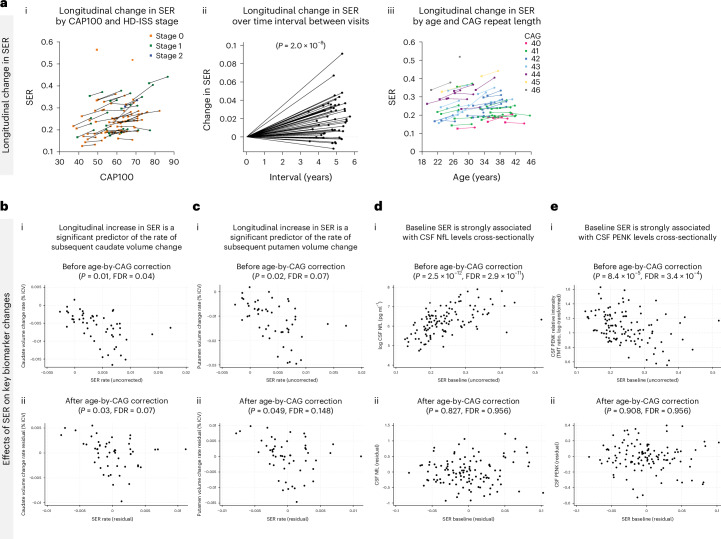
Effects of somatic expansion. **a**, Longitudinal changes in SER—(i) SER trajectories by CAP100 and HD-ISS stage with baseline visits represented by squares and follow-up visits by circles, and lines connecting data from the same individual, where stage 0 is shown in orange, stage 1 in green, and stage 2 in blue; (ii) changes in SER between visits and (iii) changes in SER by age and CAG repeat length. **b**,**c**, Associations between longitudinal SER increase and caudate (**b**) and putamen (**c**) volume change, (i) before and (ii) after age-by-CAG correction. **d**,**e**, Associations of baseline SER with cross-sectional CSF NfL (**d**) and CSF PENK (**e**) levels, with (i) before and (ii) after age-by-CAG correction. Associations were modeled via mixed effects regression using the measure on the vertical axis as the outcome and controlled for age, sex and age-by-sex interaction. Longitudinal caudate change based on a single boundary-shift integral measure per participant was an exception where an analogous ordinary least-squares model was employed.

Significant longitudinal increases in the somatic expansion ratio (SER) were detected in blood DNA in the HDGE group over 4.5 years (*P* = 2.0 × 10^−8^), with SER clearly increasing as early as HD-ISS stage 0 (Fig. 4a). SER rates of change were strongly influenced by an accelerating effect of CAG repeat length (*P* = 3.0 × 10^−5^).

### *HTT* allele structures

**Fig. 5:**
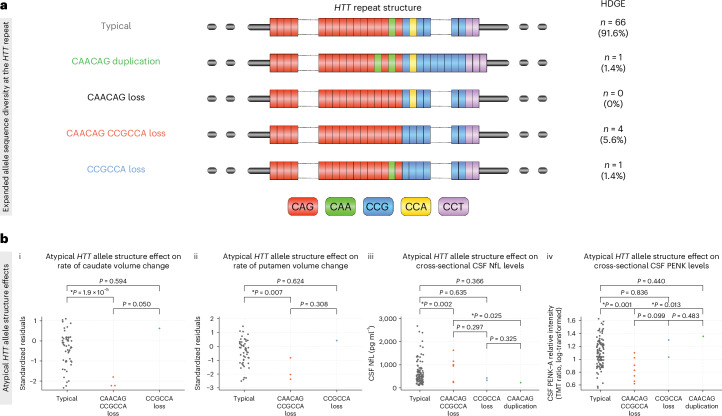
Effects of CAG architecture and allelic variants. **a**, Illustration of the *HTT* repeat structure and allelic variations in the HDGE cohort (*n* = 72), including the typical structure and four atypical variants—CAACAG duplication (green), CAACAG loss (black, not observed in cohort), CAACAG CCGCCA loss (red) and CCGCCA loss (blue). **b**, Illustration of atypical allele differences for key biomarker measures. The ANOCOVA models controlled for CAG length, sex and age, including age interactions with CAG and sex. Participant-specific random effects were included except for caudate change based on one boundary-shift integral per participant. (i) The effect on caudate volume change, with significant differences between typical alleles and CAACAG CCGCCA loss (*P* < 0.0001) and a trend with CCGCCA loss (*P* = 0.050). (ii) The effect on putamen volume change, with significant differences between typical alleles and CAACAG CCGCCA loss (*P* = 0.007). (iii and iv) Cross-sectional effects on CSF NfL (iii) and CSF PENK (iv) levels, respectively, with significant differences noted for CAACAG CCGCCA loss. Statistically significant comparisons (*P* < 0.05) are indicated by asterisks. Please note no longitudinal imaging for CAACAG duplication, hence no plot in (i) or (ii).

The majority of the HDGE group exhibited the typical *HTT* repeat structure on their expanded allele (*n* = 66, 91.6%), while a small subset (*n* = 6) showed atypical allelic variations (Fig. 5a). Specifically, the CAACAG duplication was observed in 1 (1.4%) participant, the CAACAGCCGCCA double loss was found in 4 (5.6%) and 1 (1.4%) had the CCGCCA loss.

### Predictors of progression

Baseline NfL, both plasma and CSF, and CSF PENK were predictors of atrophy over time in all brain regions (all FDR < 0.04), even after controlling for the effect of age and CAG (all FDR < 0.12; Extended Data Table 4). Rate of change in caudate and putamen was most strongly associated with change in CSF NfL (*P* = 3.0 × 10^−4^, FDR = 0.003 and *P* = 2.2 × 10^−4^, FDR = 0.003, respectively) and plasma NfL (*P* = 0.002, FDR = 0.01 and *P* = 0.03, FDR = 0.06, respectively) and the association remained after controlling for age and CAG effects (all FDR < 0.09). Rates of change in caudate and putamen were also associated with longitudinal change in CSF PENK before (*P* = 2.0 × 10^−4^, FDR = 0.003 and *P* = 1.0 × 10^−4^, FDR = 0.001, respectively) and after (*P* = 0.002, FDR = 0.021 and *P* = 9.0 × 10^−4^, FDR = 0.011, respectively) age-by-CAG correction.

Longitudinal increase in SER was a significant predictor of the rate of subsequent caudate volume change before (*P* = 0.01, FDR = 0.04) and after age-by-CAG correction (*P* = 0.03, FDR = 0.07; Fig. 4b). Longitudinal increase in SER was also a significant predictor of the rate of subsequent putamen volume change before (*P* = 0.02, FDR = 0.07) and after (*P* = 0.049, FDR = 0.148) age-by-CAG correction (Fig. 4c). Baseline SER was strongly associated with cross-sectional levels of CSF NfL (*P* = 2.5 × 10^−12^, FDR = 2.9 × 10^−11^; Fig. 4d) and CSF PENK (*P* = 8.4 × 10^−5^, FDR = 3.4 × 10^−4^; Fig. 4e) before age-by-CAG correction. However, these associations did not remain significant after the correction (CSF NfL—*P* = 0.827, FDR = 0.956; CSF PENK—*P* = 0.908, FDR = 0.956).

**Extended Data Fig. 3:**
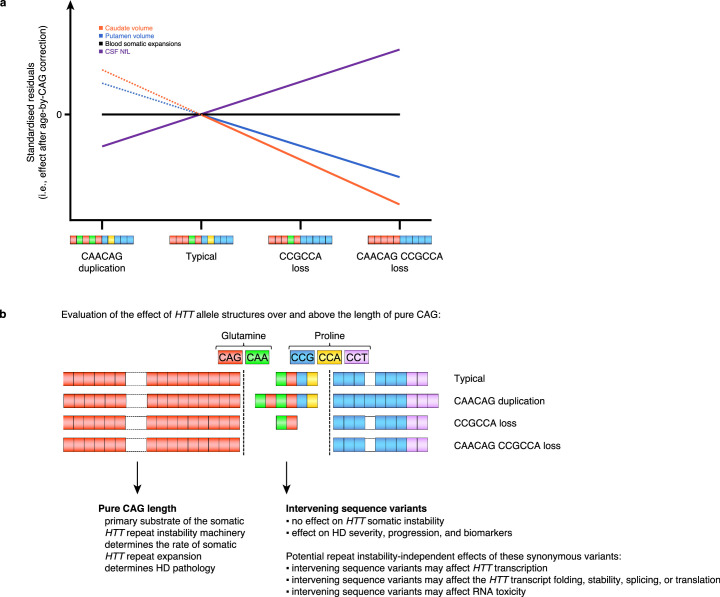
The effect of atypical *HTT* allele structure on blood somatic expansions does not explain their effect on HD phenotypes and biomarkers. (**a**) Schematic representation of the absence of effect of the atypical *HTT* allele structure on blood somatic expansion in the HD-YAS cohort and of their relative effect on caudate and putamen volumes and on CSF NfL levels after age-by-CAG correction. (**b**) Graphical representation of the *HTT* allele structures observed in the HD-YAS cohort. On the left-hand side is the pure CAG tract, which is likely the primary substrate of the somatic *HTT* repeat instability machinery and is the primary determinant of the rate of somatic HTT repeat expansion and HD pathology. Center, the sequence variants intervening between the CAG and CCG tracts, which define the atypical *HTT* allele structures observed (that is, CAACAG duplication, CCGCCA loss and CAACAG CCGCCA loss).

After controlling for CAG, age, age-by-CAG, sex and SER effects, compared to typical allele structure, the loss of CAACAG CCGCCA atypical allele had significant effects on rates of caudate (*P* = 1.90 × 10^−5^; Fig. 5b.i) and putamen (*P* = 0.007; Fig. 5b.ii) atrophy as well as cross-sectional CSF NfL (*P* = 0.002; Fig. 5b.iii) and CSF PENK (*P* = 0.001; Fig. 5b.iv) levels, with the loss of the intervening CAACAG CCGCCA associated with an accelerated neurodegenerative course (Extended Data Fig. 3). Notably, after correction for pure CAG length, there was no detectable association between atypical allele structure and SER (FDR > 0.15).

### Sample size calculations

Extended Data Table 5 shows hypothetical sample size calculations for those variables with significant longitudinal effects in the HDGE group. For a 50% treatment effect over 2 years in stages 0 and 1, total sample sizes would be 232, 282 and 326 for rates of change in CSF NfL levels, caudate and putamen volume, respectively. For a 3-year trial, these numbers would be reduced to 104, 126 and 146, respectively.

## Discussion

**Fig. 6:**
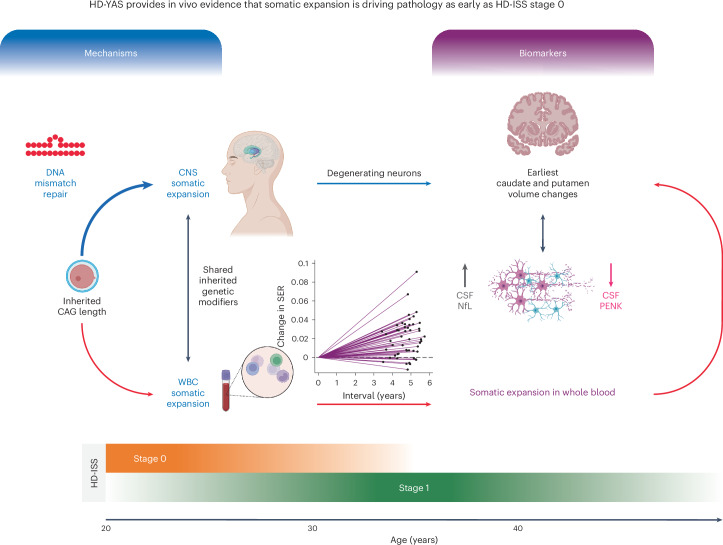
Graphical abstract. This graphical abstract illustrates the proposed pathways linking somatic expansion and its effects on biomarkers in HD-YAS. Inherited CAG repeat length is identified as the primary driver of disease progression in HD^57^. Red arrows represent observed data associations in HD-YAS and blue arrows reflect assumed causal relationships. The black bidirectional arrow under mechanisms indicates somatic expansion in WBCs as a proxy for CNS expansion, based on shared inherited genetic modifiers^14,17^. The black bidirectional arrow under biomarkers shows associations between elevated CSF NfL (marker of neuroaxonal damage) and reduced CSF PENK (surrogate of striatal MSN state) with the earliest caudate and putamen volume changes. Somatic expansion is influenced by inherited CAG repeat length, age and DNA mismatch repair gene variants^14,17^. DNA mismatch repair, highlighted in the schematic and shown as a repeat loop-out mismatch icon, is a key mechanism linking inherited CAG repeat length to somatic expansion^16^ with repair activity increasing with longer CAG repeat lengths^58^. Within the CNS, somatic expansions substantially contribute to disease progression, as supported by recent postmortem research^8^. While brain biopsy would be the gold standard for direct assessment of CNS somatic expansion in vivo, WBC-derived somatic expansion is detectable peripherally, showing early and longitudinal changes by HD-ISS stages 0 and 1. The biomarkers section shows associations of somatic expansion with the earliest caudate and putamen volume changes, and CSF NfL and PENK levels. The age continuum from HD-ISS stage 0 to stage 1 illustrates early detection of somatic expansion and biomarker changes, influencing pathology from stage 0 onward. HD-YAS provides in vivo evidence that somatic expansion drives early pathology during these early stages, highlighting its potential as a promising therapeutic target in proof-of-concept clinical trials at HD-ISS stages 0 and 1 to slow or prevent further neurodegeneration before clinical motor diagnosis. WBC, white blood cell. The figure is created with BioRender.com.

We have used state-of-the-art multimodal measures of cognition, neuroimaging, genetics and biofluid markers in a new assessment battery to study a unique cohort of young adult HDGE who were at baseline, on average, approximately 23 years before predicted clinical motor diagnosis, comparing them to matched controls in an unprecedented level of detail. Our baseline cross-sectional data identified early signs of neurodegeneration despite the maintenance of intact brain function^25^ and here we present 4.5-year follow-up data with important new mechanistic insights into what drives neurodegeneration in humans carrying the HD mutation (Fig. 6).

Our data highlight the role of inherited CAG repeat length and somatic expansion on neurodegeneration, decades before clinical motor diagnosis. We identify brain atrophy, elevated levels of CSF NfL, a marker of neuronal damage, and reduced levels of CSF PENK, a marker of striatal MSN state, in the earliest adult HD cohort studied to date. Despite evidence for the start of the neurodegenerative process, there is an absence of any decline in cognitive, motor or neuropsychiatric function at HD-ISS stages 0 and 1. Notably, we show that somatic CAG repeat expansion measured longitudinally in blood, a validated measure of somatic expansion in living patients^14,17^, is a predictor of the effect of CAG repeat length on striatal markers of very early neurodegeneration.

Consistent with the elevated levels of CSF NfL we reported at baseline^25^, we now show substantially greater rates of increase in CSF NfL in HDGE compared to controls, indicating accelerating neuroaxonal injury from the earliest stages. Most notably, the rate of change in CSF NfL in HD-ISS stage 0 was at least as fast as in stage 1, suggesting rapid neuroaxonal injury increases even before reaching the threshold of caudate or putamen volumetric loss cutoff for stage 1. Interestingly, mean CSF NfL levels were higher in HD-ISS stage 0 to 1 progressors compared to nonprogressors in both stage 0 and stage 1. The annualized rates of increase in CSF NfL across the whole HDGE group (mean = 63.38 pg ml^−1^ yr^−1^) are slightly lower than those reported in the previous HD-CSF cohort (mean = 79.16 pg ml^−1^ yr^−1^)^27^, which is consistent with the HD-YAS cohort being towards the beginning of the neurodegenerative process.

Axonal damage and injury lead to leakage of NfL into the CSF^28–30^ and are elevated in active inflammation^31^. NfL is a nonspecific marker of neuronal injury, and elevated levels have been reported in other neurodegenerative conditions^32–39^. Increases in CSF NfL are not necessarily attributable to neuronal death and could result from other degenerative processes such as leaky axons. Nevertheless, it is a clear marker of neuroaxonal pathology and therefore understanding CSF NfL temporal dynamics and kinetics can provide valuable insights into mechanisms in neurodegenerative diseases^29^.

Previously, cross-sectional studies have revealed lower levels of CSF PENK in manifest HD compared to other neurodegenerative conditions^40^, as well as compared to HDGE before clinical motor diagnosis and controls^41,42^. Our longitudinal findings in a larger cohort, and our demonstration of a significant association between PENK levels and striatal imaging measures, serve to substantially strengthen the rationale for using PENK as a surrogate marker for striatal MSN state.

Astrocytes are implicated in disease processes through both cell-autonomous and non-cell-autonomous mechanisms^43,44^, with one key study identifying a core signature of astrocyte genes with expression altered by mHTT in both humans and mouse models^44^. A recent study provided the first evidence of mHTT-induced alterations in basal pro-inflammatory cytokine production in microglia without immune stimulation, along with a reduction in endocytic and phagocytic activity in mHTT-bearing microglia under basal conditions, suggesting a possible role for microglial cell-autonomous inflammation and activity in the early stages of HD^45^. Consistent with our previous findings of elevated microglial marker CSF YKL-40 levels at baseline^25^, we now show greater rates of increase in CSF YKL-40 longitudinally in the HDGE group compared to controls. However, we do not observe significant longitudinal changes in pro-inflammatory cytokine markers IL-6 and IL-8, which are components of the innate immune system, nor in GFAP, an intermediate filament protein of astrocytes associated with astroglial activation^46^. It is known that mHTT is expressed in microglia^47^ and that microglial activation correlates with severity later in the disease^48^, where mHTT-induced dysfunction of central nervous system (CNS) immune cells is closely linked to pathogenesis^49^. We postulate that the isolated elevation of YKL-40 may be due to both cell-autonomous and non-cell-autonomous mechanisms at play with activation driven by mHTT dysregulation of astrocytes, rather than general gliosis, which would be additionally indicated by a concomitant rise in GFAP. Our findings suggest that astrocytic dysfunction is more prominent than any abnormal innate immune response at this stage of the disease, as IL-6 and IL-8 levels, which are upregulated in HD and correlate with disease progression^49,50^, remained unchanged longitudinally, reinforcing the importance of treating early at this stage, before widespread neuroinflammation occurs.

The presence of neuronal damage within HD-ISS stage 0 is further supported by the evidence of substantially elevated rates of brain atrophy and a corresponding reduction in CSF PENK levels. Stages 0 to 1 progressors also had substantially higher elevations in CSF NfL than stage 0 nonprogressors. The substantially higher rates of caudate and putamen atrophy and global brain measures and their association with disease burden suggest that neurodegenerative processes are already occurring across our cohort and at the earliest ages observed in this study. This atrophy was measurable in those with basal ganglia volumes distributed throughout the volume range observed in unaffected controls, implying the beginning of detectable neurodegeneration. In addition to these changes seen at the macrostructural level, diffusion imaging provides evidence that there is ongoing very early microstructural white matter damage. The strong predictive power of baseline NfL (in both plasma and CSF) for subsequent atrophy in all brain regions further supports the suggestion that there is early neuroaxonal damage which leads to macroscopic effects such as brain atrophy.

Despite the evidence of ongoing pathological changes in our stages 0 and 1 cohort, neurodegeneration is not yet impacting measurable function as we saw no significant disease-related decline in any of the cognitive, neuropsychiatric or functional measures. Previous work has shown that such changes only become evident from HD-ISS stage 2 (ref. ^51^).

**Extended Data Fig. 4:**
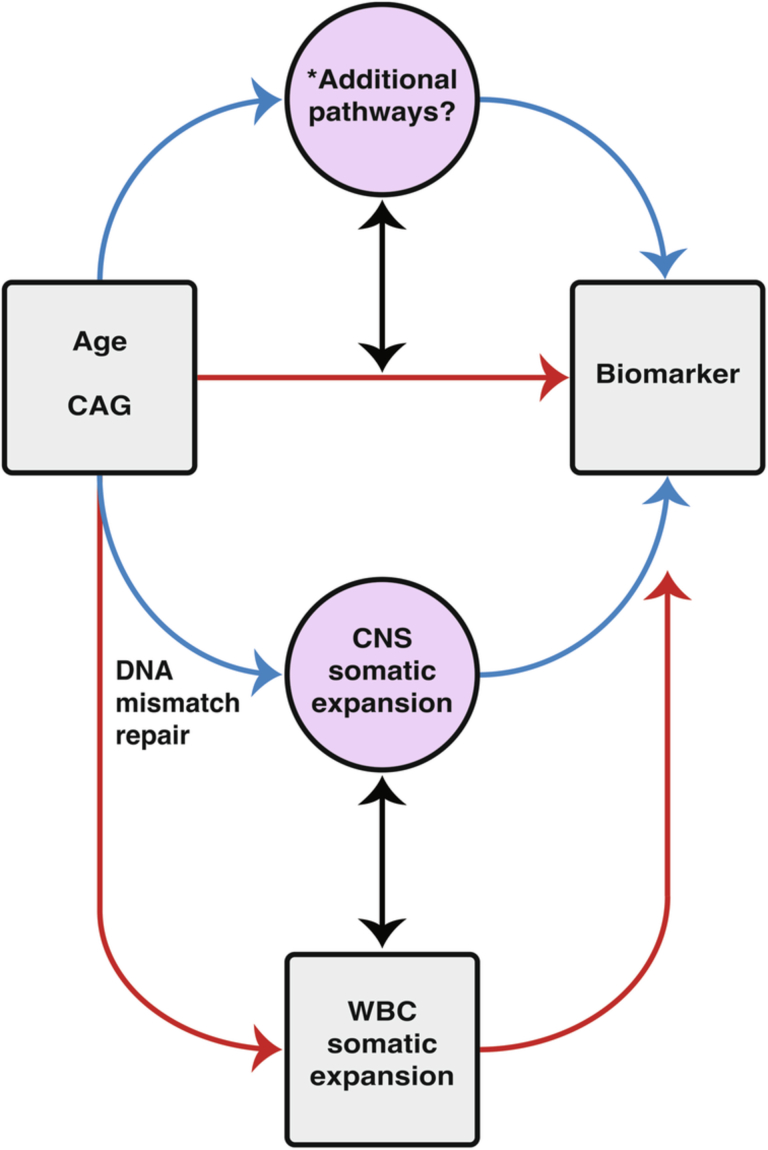
Conceptual causal model. Conceptual model illustrating the causal relationships among age-related CAG repeat length (age, CAG), CNS somatic expansion, WBC somatic expansion and biomarkers. The squares in gray depict measured data, while the circles in purple represent unobservable quantities. The black, bidirectional arrow signifies the assumption that somatic expansion in WBCs serves as a proxy for somatic expansion in the CNS. Red arrows indicate correlations between observable data and the underlying biological processes of interest. Blue arrows are presumed to reflect true causal pathways indirectly. We note that DNA mismatch repair is a key mechanism linking inherited CAG repeat length to somatic expansion, with mismatch repair activity increasing with longer CAG repeat lengths. *Note additional pathways could be involved due to the imprecise and indirect measurement of actual CNS expansion. Abbreviations: CNS=Central Nervous System. WBC=White Blood Cell.

We demonstrate the accumulation of somatic expansion of the *HTT* CAG repeat in blood DNA over time in HD-ISS stages 0 and 1 and, critically, show that it is associated with both brain atrophy and CSF NfL, a marker of neuronal–axonal injury, and CSF PENK, a surrogate marker of striatal MSN state. A higher inherited CAG length was associated with a faster increase in SER over time. SER was associated with caudate and putamen atrophy, both cross-sectionally and longitudinally, even after controlling for age-by-CAG interactions. Baseline SER was strongly associated with cross-sectional levels of CSF NfL and CSF PENK before age-by-CAG correction; however, these associations did not remain significant after the correction. We postulate that bioassay measurements demonstrate higher variability and noise compared to striatal volume measurements. Therefore, the lack of significance in associations with CSF NfL and PENK does not undermine the significant association between the longitudinal increase in SER and volumetric changes in the caudate and putamen. Additionally, the statistical strength of the influence of CAG length on atrophy was weakened in models also controlling for blood SER. Assuming that, via the common baseline CAG length effects and shared genetic modifiers, SER measured in blood is an indirect quantifiable indicator of the greater somatic expansion occurring in neurons, these results may be seen as providing in vivo evidence for the key role of somatic CAG repeat expansion in very early HD pathology in humans (Extended Data Fig. 4), reinforcing the putative pathological role of somatic expansion as a critical factor in disease progression^5–9^.

If the recent suggestion from HD postmortem brains that asynchronous somatic expansion leads to asynchronous stochastic crossing of the transcriptional dysregulation threshold and asynchronous neuronal death^8^ is correct, then our data would support the hypothesis that somatic expansion is already an active process in the brain and that some neurons have already crossed a critical repeat length threshold ~20 years before clinical motor diagnosis. Indeed, this phenomenon is both predicted by the stochastic models and consistent with autopsy observations of early neuronal loss^8^. This would suggest that suppressing CAG repeat somatic expansion from this point in the disease process could prevent additional neurons from passing the neuronal toxicity threshold and reduce neurodegeneration before functional deficits are manifest. Therapeutic agents targeting DNA repair proteins that modify somatic expansion show great potential, with MSH3 as a particularly attractive target for HD and other repeat expansion disorders^52^, and various MSH3-targeting therapeutics are currently under development^53,54^. To this end, somatic expansion of CAG repeats in blood DNA could be a useful biomarker to demonstrate target engagement of somatic expansion-suppressing therapies with peripheral exposure.

Within our cohort, a small number of individuals carried atypical CCGCCA or CAACAG CCGCCA loss of intervening sequence *HTT* alleles. These atypical structures have a high potential to cause mid-estimation of the CAG repeat length^13,14,17–19^, and using the MiSeq-derived CAG lengths changed the mean baseline years to predicted clinical motor diagnosis in HD-YAS from 24 to 23 years. After correcting for pure CAG length, these structures have previously been associated with earlier clinical motor diagnosis^13,14,17–19^. Consistent with this, we find those participants with the loss of intervening sequence structures exhibit higher rates of caudate and putamen atrophy, and have some of the greatest elevations in CSF NfL and reductions in CSF PENK, which together suggest an acceleration of the degenerative process (Fig. 5b). Detecting these effects in such small numbers so early in the course of disease suggests these synonymous DNA structural differences are exerting a substantial influence on the rate of neuropathological change. Interestingly, after correcting for pure inherited CAG there was no residual association between these allele structure variants and SER. This is consistent with previous work in other cohorts in blood, postmortem brains and cell lines^14,17,19^ showing that the loss of the intervening CAACAG CCGCCA does not increase the rate of CAG expansion over and above the effects of pure CAG length. Relevant available brain data is limited so it is still possible that the CAACAG CCGCCA loss increases CAG expansions in brain but not blood. An alternative hypothesis is that, after correcting for pure CAG, the residual disease-modifying mechanism of the CAACAG CCGCCA loss is independent of somatic expansion of the *HTT* repeat via effects on RNA transcription, RNA stability, or canonical or repeat-associated non-ATG translation (Extended Data Fig. 3). Regardless, these variants clearly have a profound impact on the disease course.

This work not only provides evidence to support the potential of therapies targeting somatic expansion but also identifies robust markers of disease progression, which may have utility as likely surrogates for future preventative clinical trials. CSF NfL, PENK and brain atrophy measures have the potential to monitor disease progression in HD-ISS stages 0 and 1, where clinical endpoints are not applicable. Change in CSF NfL level has previously been used as an outcome measure for a trial of the ASO nusinersen^55^ in children with spinal muscular atrophy. Earlier treatment initiation was also associated with a larger decrease in CSF NfL levels, underscoring the importance of early intervention to preserve neuronal health.

At this stage of the disease, CSF mHTT levels are very low, with only 38.3% of samples in the HDGE group exceeding the detection level. These findings underscore the limitations of available CSF mHTT assays and confirm there is an urgent need for a reliable assay capable of detecting very low concentrations of mHTT in HDGE, ideally at attomolar levels, if HTT-lowering therapies are to be pursued in stage 0 and 1 HDGE cohorts.

Our extensive phenotypic characterization of HD-ISS stages 0 and 1 may allow us to enrich recruitment for future preventative trials. For example, we demonstrate that baseline NfL and PENK levels predict subsequent brain atrophy, and the potential to establish cutoffs for enriching HD-ISS stage 0 based on these biofluids holds significant promise. Harmonization of HD-YAS with existing cohorts across the disease spectrum such as HD-CSF and HDClarity (ClinicalTrials.gov: NCT02855476) will help to establish reliable cutoffs for inclusion. Another important consideration in clinical trial design is that atypical repeat structures, although infrequent, substantially affect disease progression and may additionally impact therapeutic efficacy. Identification of these rare cases through MiSeq will be important to control for these effects and more accurately assess treatment efficacy.

If these biomarkers can serve as likely surrogate outcomes, sample size calculations suggest feasible numbers for clinical trials in an HD-ISS stage 0/1 cohort given sufficiently large treatment effects. For example, in a clinical trial over 3 years with a 50% treatment effect, 104 participants would be required with CSF NfL as an outcome measure, with 126 for caudate and 146 for putamen atrophy. Notably, the caudate boundary-shift integral measure of change we use here is already well-validated and has previously been used in the laquinimod trial in HDGE with a clinical motor diagnosis^56^.

In summary, the results presented strongly support the hypotheses that individual-specific somatic expansion in blood DNA predicts individual-specific somatic expansion in the brain. We show in living participants, decades before clinical motor diagnosis, that somatic expansion of the CAG repeat appears to be an important driver of the earliest pathological disease processes, as evidenced by its association with striatal atrophy rates and CSF NfL and PENK levels. Somatic expansion of repeats underlying disease pathogenesis is likely relevant to many repeat expansion diseases, where similar DNA repair mechanisms may play a role. With new therapies in development to target the DNA repair proteins that are known to influence somatic expansion, our results are timely in demonstrating its association with measurable disease markers. By intervening with therapies targeting somatic CAG repeat expansion at the start of the neurodegenerative process, that is, HD-ISS stages 0 and 1 decades before clinical motor diagnosis, while function remains intact, there is the very real possibility that treatments can delay or even prevent the appearance of clinical signs. To this end, we have identified robust measures of early pathology with potential to act as possible biomarker surrogates of disease progression, and identified the ideal cohort for intervention to delay or prevent clinical motor diagnosis.

## Methods

### Participant characteristics

Participants were recruited across the UK and enrolled at one study site (University College London (UCL)). The inclusion criteria are detailed in the Supplementary Methods. Participants (131 in total, 64 HDGE and 67 controls) attended at baseline and 103 (57 HDGE and 46 controls) returned for follow-up approximately 4.5 years later.

The study was registered on ClinicalTrials.gov (NCT06391619) where the study protocol and the predefined statistical analysis plan are provided. All participants underwent comprehensive assessment of clinical, cognitive and neuropsychiatric function, neuroimaging, blood sampling, and optional CSF collection consistent with the baseline procedure (Supplementary Methods)^25^. See Extended Data Table 6 for a list of assessments and Supplementary Table 5 for missing data. Supplementary Methods provides further details for all assessments.

### Ethics

The study received approval by the London—Bloomsbury Research Ethics Committee (22/LO/0058). All study procedures adhered to principles outlined in the Declaration of Helsinki, and before enrollment, written consent was obtained from all participants.

### Clinical, cognitive and neuropsychiatric assessments

A clinical examination was performed including assessment for lumbar puncture suitability.

All the HDGE participants with longitudinal neuroimaging (*n* = 54) were staged according to the HD-ISS^26^ at each visit. The longitudinal pipeline of FreeSurfer version 6 (https://surfer.nmr.mgh.harvard.edu/pub/dist/freesurfer/) was used to derive caudate and putamen segmentations to classify stages 0 and 1, and stage 2 was defined by participants reaching the age- and education-adjusted cutoffs for the Symbol Digit Modalities Test and/or Total Motor Score. Additionally, predicted years to clinical motor diagnosis were linked to the standardized CAG-Age-Product (CAP) score. A CAP score of 100 occurs at the CAG-specific expected age of motor diagnosis^59^.

All cognitive and neuropsychiatric tasks from baseline were repeated at follow-up with two exceptions. Due to participant feedback, we replaced the Progressive Ratio Task from baseline with the Goals Prior Assay task at follow-up to assess the motivational domain. Additionally, Stroop interference was included within the core cognitive tasks in follow-up. See Supplementary Methods and Supplementary Figs. 3–5 for details of the cognitive and neuropsychiatric testing battery.

### Neuroimaging

Scanning was performed on a 3T Siemens Prisma (Siemens Healthineers, Erlangen, Germany) and parameters were consistent between baseline and 4.5-year follow-up. Neuroimaging assessments included volumetric T1-weighted imaging (T1W), DWI and MPM. We applied the same predefined regions of interest (ROI) that were examined at baseline. Volumes were extracted from T1W images and mean values across the relevant ROI were derived for (1) standard DWI and neurite orientation and dispersion density imaging (NODDI) metrics^60^, (2) structural connectivity (right-hand dominant participants only) and (3) MPMs.

Longitudinal imaging changes were derived by subtraction of values, except for direct measures of change for some volumetric ROIs. The boundary-shift integral is a direct measure of change between positionally matched (registered) serial images, which is more sensitive to longitudinal change than subtraction^61^. This technique was used for whole brain, ventricles and caudate^62^. Within-participant voxel-compression maps were derived and convolved with gray and white matter maps generated by voxel-based morphometry^24^ to estimate volume change within these tissues.

Details of diffusion, structural connectivity and MPM processing pipelines are provided in Supplementary Methods and Supplementary Fig. 6.

### Biofluids

Biofluids were collected at baseline and follow-up under the same standardized, well-validated conditions, methods and equipment^63^. To remove potential batch effects, biofluid samples collected at baseline were reanalyzed in parallel with the follow-up samples, employing the assays detailed in Supplementary Table 19. Quantification of analytes was performed blinded to group status.

Measurements in CSF included mHTT, NfL protein, total tau (tau), GFAP, UCH-L1, YKL-40 (also known as chitinase-3 like-protein-1 (CHI3L1)), IL-6 and IL-8. In plasma, NfL, tau, GFAP and UCH-L1 were quantified. The Neurology 4-Plex A (GFAP, NfL, Tau and UCH-L1) was measured in singlicate, yielding the following inter-plate coefficients of variation (%CV): CSF GFAP (3.7%), CSF NfL (8.2%), CSF Tau (11.1%), CSF UCH-L1 (62.8%), plasma GFAP (5.6%), plasma NfL (5.0%), plasma Tau (11.1%) and plasma UCH-L1 (63.8%). The %CVs were calculated from duplicate measurements of internal plate controls made of pooled human plasma. CSF IL-6, IL-8 and YKL-40 were measured in duplicate, with the following %CVs: CSF YKL-40 (10.4%), CSF IL-6 (29.2%) and CSF IL-8 (12.2%). CSF mHTT was measured in triplicate and the relative abundance of CSF PENK was quantified in single measurements.

Unbiased liquid chromatography-mass spectrometry (LC–MS)-based proteomics analysis was performed using the tandem mass tag (TMT) technique^64^ to measure relative abundance of CSF PENK. CSF samples were prepared including an initial multi-affinity depletion step to reduce interference by high-abundant blood-derived proteins (Supplementary Methods). Following this step, samples were subjected to reduction and alkylation of cysteine residues, digestion with trypsin and endoproteinase Lys-C and isobaric labeling using TMTpro 18-plex reagents^65^ (Thermo Fisher Scientific). TMT multiplex peptide samples were fractionated by high-pH reversed-phase high-performance LC (HPLC)^66,67^, and analyzed by nano-HPLC (EasyLC, Thermo Fisher Scientific) coupled to a high-resolution Orbitrap hybrid mass spectrometer (Orbitrap Lumos Tribrid, Thermo Fisher Scientific). Protein identification and data processing for quantification was performed using Proteome Discoverer 2.5 (Thermo Fisher Scientific), and R Statistics. More details are provided in Supplementary Methods.

### *HTT* CAG repeat structure and somatic expansion

DNA was extracted from whole blood using the chemagic 360-D instrument (Perkin Elmer) for automated DNA extraction. The modal length of the pure *HTT* CAG repeat, the *HTT* repeat structure and quantity of blood *HTT* somatic expansions in the HDGE group were determined by ultra-deep amplicon MiSeq sequencing^14,68^. The *HTT* MiSeq reads were processed and genotyped using ScaleHD (v1 (ref. ^14^)) and RGT (https://github.com/hossam26644/RGT). The SER^14^ of the *HTT* CAG repeat was quantified from MiSeq data at baseline and follow-up, allowing for longitudinal assessment of somatic expansion changes during the interval between the two visits. CAG repeat length estimated using MiSeq was used for all statistical analyses of associations.

### Somatic expansion mediation models

Within the HDGE group, we estimated statistical associations between white blood cell (WBC) somatic expansion ratios with NfL, PENK and with volumetric brain measures. The analyses were performed with and without correction for age, CAG and age-by-CAG interaction. The models also controlled age, sex and age-by-sex interaction as covariates. We modeled cross-sectional SER versus biomarker relationships via random effects regression as described earlier. We estimated relationships between baseline SER and longitudinal change in SER versus longitudinal change in biomarkers using ordinary least square regression as described above for other models of volumetric direct-change rates. For NfL and putamen volumes, we calculated change rates by subtraction of baseline from follow-up values.

To assess the potential causal implications of SER-biomarker associations, we compared the statistical strength of SER as a predictor of the biomarker before and after controlling for age and CAG length (Extended Data Fig. 4 illustrates a schematic of the underlying causal reasoning). A substantially weakened SER relationship after age and CAG control would suggest that the associations may be due to mutual influence of CAG length over time without more direct causality. Conversely, we assessed the strength of the age–CAG versus biomarker relationships with and without SER control. Weakened age–CAG associations with a biomarker in the presence of both a significant predictive SER effect on the biomarker and a significant age–CAG association with SER are consistent with an intermediate causal role for CNS somatic expansion (as indirectly assessed by SER in WBC DNA). These statistical comparisons are the essence of statistical assessments of plausible causality in formal causal models. In the simple, cross-sectional case, the degree of mediation is often expressed by the relative reduction in the adjusted correlation or regression coefficient of a single distal variable. However, the analyses here involve the joint mediation of two terms—CAG length and its interaction with age. Mediation is no longer simply quantified by this approach. Hence, we instead emphasize the hypothesis testing aspect of the underlying statistical tests.

We used the same regression methods as for the somatic expansion models to assess associations between log NfL levels and volumetric outcomes. However, we attempted no causal interpretation of NfL versus volumetric relationships. Although these are both HD-related biomarkers, they are considered concurrent indicators of the same pathology and there is no well-justified conception of one of these changes ‘causing’ the other.

### Sample size estimates

Approximate sample size estimates for clinical trials involving equal allocation to one treatment group and a placebo group were calculated based on observed HDGE group versus control longitudinal models. The group differences in longitudinal rates were converted to Cohen’s *d* effect sizes using within-group longitudinal standard deviations derived from estimated random effect variances. Assumed treatment effects were then defined as a percent slowing of the group difference in longitudinal rates adjusted for the assumed trial length. These should be considered somewhat optimistic order-of-magnitude estimates. We could not estimate potential increased within-group variance over time (and resultant sample size increase) based on only two observed time points. We did not factor in trial dropout rates. On the other hand, we did not consider potential sample size reduction due to efficient (but perhaps controversial) estimates of treatment-induced slope deviation derived from repeated measures over time, but instead based calculations on net group differences at the end of a trial. See Supplementary Methods for further details.

### Statistical analysis

Unless otherwise noted, all statistical analyses were performed in accordance with a prespecified statistical analysis plan. Analyses testing the relationship between caudate and putamen volumes or NfL levels to observed or predicted disease progression were defined as primary hypotheses, with a statistical significance level of *P* = 0.05. Analyses of potential disease associations for the rest of the wide-ranging test battery were considered exploratory and primarily assessed by estimated FDR calculated using the Benjamini–Hochberg method^69^. FDR was calculated separately per measurement domain (cognitive, volumetric imaging, bioassays, etc.) to examine conceptually and technically sensible sets of underlying *P* values. We suggest an FDR threshold of 15% for identifying exploratory results of interest.

Demographic group comparisons were conducted by *t* test for continuous variable, by chi-square test for sex differences and by Mann-Whitney U test for education level. Both the longitudinal and repeated-measure cross-sectional analyses were performed by maximum likelihood random effect regression modeling with (necessarily) only random intercepts per participant. The primary regression predictors of interest were group status (control versus HDGE) and in models testing age and CAG effects, terms for age, MiSeq-derived CAG length and age-by-CAG interactions nested within the HDGE. The joint significance of age and CAG effects were estimated by the maximum likelihood test for differences in model fit. The models also contained covariates for sex, a non-nested age term and sex-by-age interaction. The non-nested age term was included to make HDGE group-specific aging effects estimable. Cognitive models also included International Standard Classification of Education education level and estimated intelligence quotient via the National Adult Reading Test score. Due to the consistent skewness of measured distributions, logarithmic transformations were used on all bioassay concentration measures.

Longitudinal volumetric analyses of brain structures other than putamen relied on a single measure of volume change per participant derived from pairs of baseline and follow-up scans. These changes were converted to annual rates and modeled by ordinary least squares regression with predictor variables analogous to those listed above. All other models of longitudinal change used total values at the two visits as outcomes and longitudinal effects of primary predictors and covariates were estimated by interactions with follow-up time between the visits. Those models also retained all baseline effects for predictors and covariates. To preserve the unbiased estimation of model parameters when data is missing at random, baseline data from participants with no follow-up were included in the model.

### Reporting summary

Further information on research design is available in the Nature Portfolio Reporting Summary linked to this article.

## Online content

Any methods, additional references, Nature Portfolio reporting summaries, source data, extended data, supplementary information, acknowledgements, peer review information; details of author contributions and competing interests; and statements of data and code availability are available at 10.1038/s41591-024-03424-6.

## Supplementary information

Supplementary Information Supplementary Methods, results and discussions, Tables 1–19 and Figs. 1–6.

Reporting Summary

## Figures and Tables

**Extended Data Table 1:**
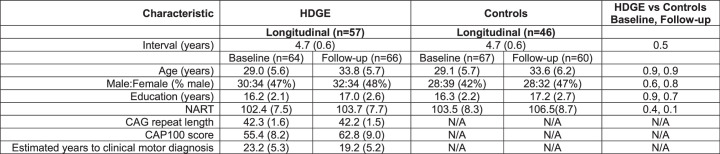
Participant demographic characteristics

Table presenting longitudinal participant demographics in HD-YAS. Values are means (SD), n (%) or median (IQR). Two-tailed group comparisons were made using *t* tests (interval, age, education, NART) and *χ*^2^ tests (sex). CAP100, CAG–age product, whereby CAP100 is the expected age of diagnosis. HDGE, HD gene expanded; IQR, interquartile range; NA, not applicable; NART, National Adult Reading Test, an estimate for IQ; SD, standard deviation; IQ, intelligence quotient.

**Extended Data Table 2:**
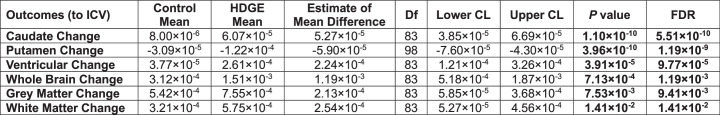
Longitudinal volumetric data

Table showing longitudinal volumetric outcomes (normalized to ICV) for HDGE participants compared to controls. Volumetric analyses for brain structure longitudinal changes, excluding the putamen, modeled a single change measure per paired participant scans (boundary-shift integral or voxel-based morphometry convolution) after conversion to annual change rates. These changes were modeled by ordinary least squares regression. Putamen changes were derived from subtraction of baseline and follow-up MALP-EM segmentations divided by follow-up length. Analysis results and residual adjustments reflect control for baseline age, sex, and their interaction. Statistical two-sided group comparisons were adjusted for multiple comparisons using the FDR, with P values, degrees of freedom, and confidence limits provided in the table. Significant values at FDR < 0.15 are highlighted in bold. CL, confidence limit; Df, degrees of freedom; FDR, false discovery rate; HDGE, HD gene expanded; ICV, intracranial volume.

**Extended Data Table 3:**
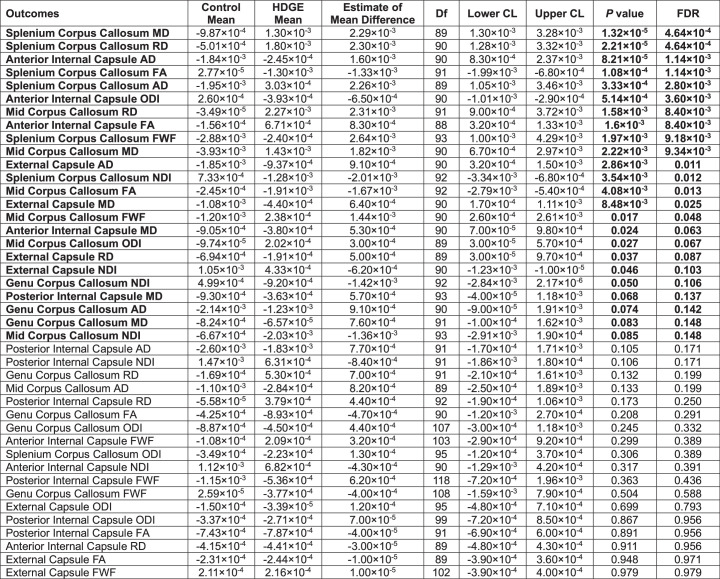
Longitudinal diffusion data

Diffusion (AD, FA, MD, and RD) and NODDI (FWF, ODI, and NDI) metrics were derived for the following regions of interest: corpus callosum (genu, mid and splenium), internal capsule (anterior and posterior) and external capsule. Longitudinal change was derived by subtraction of baseline from follow-up value and change was annualized. Analysis results and residual adjustments reflect control for baseline age, sex and their interaction. Statistical two-sided group comparisons were adjusted for multiple comparisons using the FDR, with P values, degrees of freedom, and confidence limits provided in the table. Significant values at FDR < 0.15 are highlighted in bold. AD, axial diffusivity; CL, confidence limit; Df, degrees of freedom; FA, fractional anisotropy; FWF, free water fraction; HDGE, HD gene expanded; MD, mean diffusivity; NDI, neurite density index; ODI, orientation dispersion index; RD, radial diffusivity.

**Extended Data Table 4:**
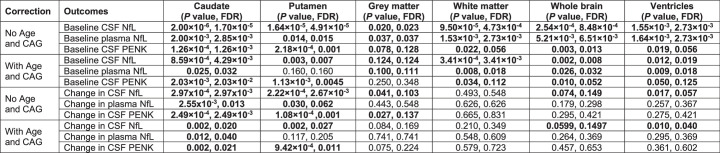
Predictors of brain atrophy measures

Table summarizing the association between baseline and change in key fluid biomarkers with brain atrophy measures across different brain regions, both with and without adjustment for age and CAG repeat length. The primary regression predictors of interest were group status (control vs. HDGE) and in models testing age and CAG effects, terms for age, MiSeq-derived CAG length, and age-by-CAG interactions nested within the HDGE. The joint significance of age and CAG effects were estimated by the maximum likelihood test for differences in model fit. The models also contained covariates for sex, age and sex-by-age interaction. Longitudinal volumetric analyses of brain structures other than putamen relied on a single measure of volume change (boundary-shift integral or voxel-based morphometry convolution) per participant derived from pairs of baseline and follow-up scans. These changes were converted to annual rates and modeled by ordinary least squares regression with predictor variables analogous to those listed above. All other models of longitudinal change used the difference between the two visits as outcomes and longitudinal effects of primary predictors and covariates were estimated by interactions with follow-up time between the visits. Those models also retained all baseline effects for predictors and covariates. To preserve the unbiased estimation of model parameters when data is missing at random, baseline data from participants with no follow-up were included in the model. Significant values at FDR < 0.15 are highlighted in bold. CSF, cerebrospinal fluid; FDR, false discovery rate; NfL, neurofilament light, PENK, proenkephalin.

**Extended Data Table 5:**
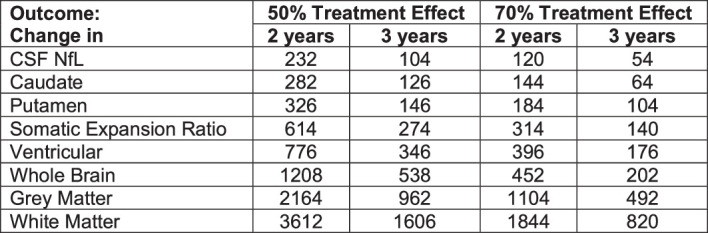
Sample size calculations

Table showing approximate sample size estimates for clinical trials involving equal allocation to one treatment group and a placebo group, which were calculated based on observed HDGE group versus control longitudinal models. The group differences in longitudinal rates were converted to Cohen’s *d* effect sizes using within-group longitudinal standard deviations derived from estimated random effect variances. Assumed treatment effects were then defined as the percent slowing of the group difference in longitudinal rates multiplied by the assumed trial length. Treatment effect assumes a reduction in the difference in rate between the HDGE and control groups. Boundary-shift integral is used to measure the change in the caudate, brain and ventricles. Gray matter and white matter changes are generated by convolving baseline voxel-based morphometry-derived gray or white tissue maps with voxel-compression maps of within-participant change. Putamen change is measured by subtraction of baseline and follow-up MALP-EM segmentations. See Supplementary Methods for further details. CSF, cerebrospinal fluid; HDGE, HD gene expanded; NfL, neurofilament light.

**Extended Data Table 6:**
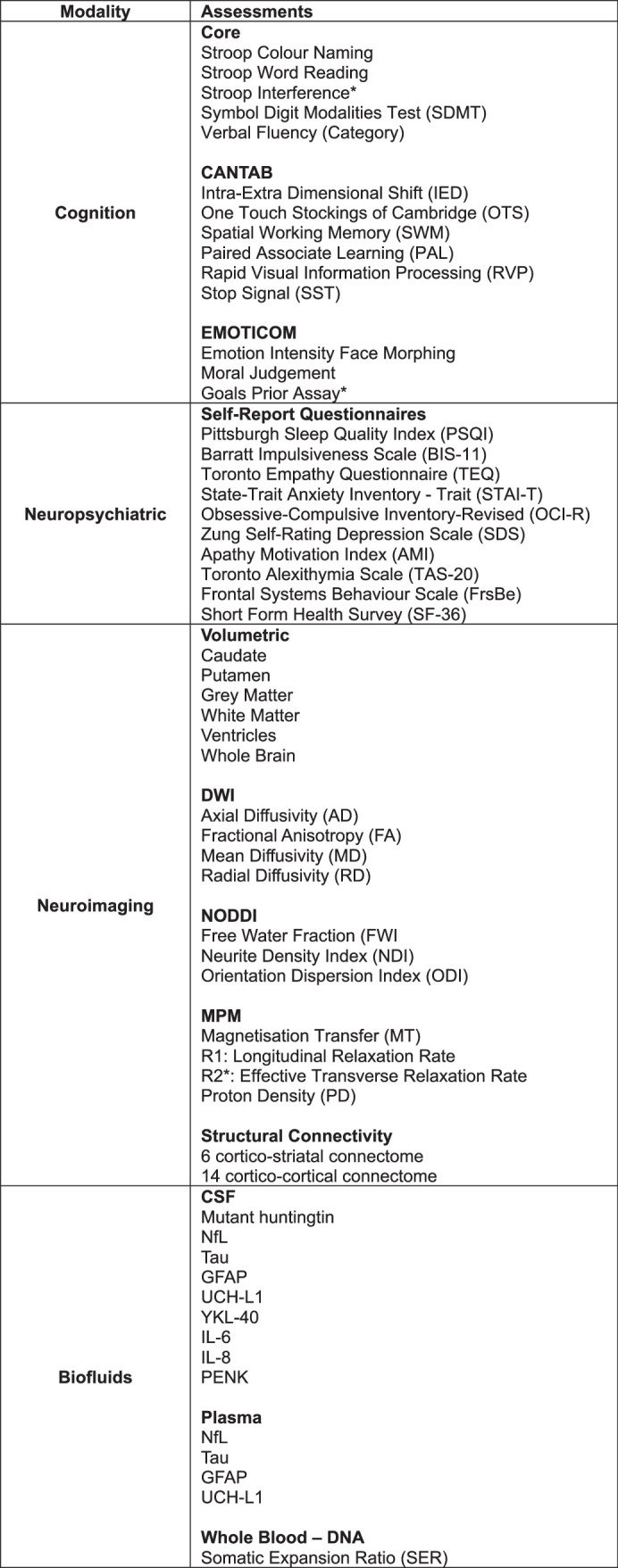
Panel of assessments

Table providing a detailed overview of assessments across the following four modalities: cognition, neuropsychiatric, neuroimaging and biofluids.

*New tasks at follow-up.

CANTAB, Cambridge Neuropsychological Test Automated Battery; CSF, cerebrospinal fluid; DWI, diffusion-weighted imaging; EMOTICOM, emotion, motivation, impulsivity and social cognition; GFAP, glial fibrillary acidic protein; IL, interleukin; MPM, multiparametric mapping; NfL, neurofilament light; NODDI, neurite orientation dispersion and density imaging; PENK, proenkephalin; UCH-L1, ubiquitin carboxyl-terminal hydrolase L1; YKL-40, also known as chitinase-3 like-protein-1 (CHI3L1).

## Data Availability

We are committed to data sharing while maintaining confidentiality due to the sensitive and potentially identifiable nature of these data. Biofluid samples will not be shared due to the limited amount of material available. The remaining samples will be required for replication for the next HD-YAS visit. Upon reasonable request, data will be made available 24 months after the end of data collection, through application via UCL to the Principal Investigator, Professor Sarah Tabrizi. Researchers will be required to submit a proposal meeting the research criteria and must demonstrate full GDPR compliance. A data access agreement with UCL will be required.

## Code availability

All software is freely available with the exception of the in-house MIDAS software used to generate the boundary-shift integral for caudate, whole brain and ventricles. This can be requested from Professor Nick Fox at the Dementia Research Centre, UCL, UK.
