# Supplementary material for: Somatic CAG repeat expansion in blood associates with biomarkers of neurodegeneration in Huntington’s disease decades before clinical motor diagnosis
Source: Nat Med. 2025 Jan 17;31(3):807–18. doi: 10.1038/s41591-024-03424-6 (PMC11922752; doi:10.1038/s41591-024-03424-6)
Supplement: Supplementary file 1 — Supplementary Methods, results and discussions, Tables 1–19 and Figs. 1–6. [file 41591_2024_3424_MOESM1_ESM.pdf]

nature medicine

Supplementary information https://doi.org/10.1038/s41591-024-03424-6

Somatic CAG repeat expansion in blood associates with biomarkers of neurodegeneration in Huntington's disease decades before clinical motor diagnosis

In the format provided by the authors and unedited

Supplementary Information

Table of Contents

Supplementary Methods ... 2
Participant characteristics ... 2
Clinical assessments ... 3
Cognitive assessments ... 3
Neuropsychiatry ... 6
Neuroimaging ... 7
Imaging quality control ... 11
Biofluids ... 12
Statistics ... 14
Supplementary Results and Discussion ... 15
Participant characteristics ... 15
Clinical ... 15
Cognitive ... 15
Neuropsychiatric ... 16
Neuroimaging ... 16
Biofluids ... 17
Predictors ... 18
Supplementary Table 1: Longitudinal cognitive data ... 20
Supplementary Table 2: Longitudinal neuropsychiatric data ... 21
Supplementary Table 3: Cross-sectional cognitive data ... 22
Supplementary Table 4: Cross-sectional neuropsychiatric data ... 24
Supplementary Table 5: Data included by modality ... 26
Supplementary Table 6: Longitudinal structural connectivity data ... 27
Supplementary Table 7: Longitudinal multiparametric mapping data ... 28
Supplementary Table 8: Cross-sectional volumetric data ... 30
Supplementary Table 9: Cross-sectional diffusion data ... 31
Supplementary Table 10: Cross-sectional structural connectivity data ... 33
Supplementary Table 11: Cross-sectional multiparametric mapping data ... 34
Supplementary Table 12: Log CSF NfL cross-sectional model ... 36
Supplementary Table 13: Log CSF PENK cross-sectional model ... 37
Supplementary Table 14: Log plasma NfL cross-sectional model ... 38
Supplementary Table 15: Cross-sectional HD-ISS progression comparisons for CSF NfL ... 39

Supplementary Table 16: Cross-sectional HD-ISS progression comparisons for plasma NfL........ 41
Supplementary Table 17: Longitudinal biofluid data 43
Supplementary Table 18: Cross-sectional biofluid data........ 44
Supplementary Table 19: Biofluid assay details........ 45
Supplementary Figure 1: Cross-sectional differences in (a) cognitive and (b) neuropsychiatric measures between the HDGE and control groups........ 46
Supplementary Figure 2: CSF mHTT assay performance........ 48
Supplementary Figure 3: Composite image of the CANTAB tests 49
Supplementary Figure 4: EMOTICOM Moral Judgment test 50
Supplementary Figure 5: Goals Prior Assay task........ 51
Supplementary Figure 6: Structural connectivity pipeline 52

Supplementary Methods

Participant characteristics

Data for the baseline assessments were collected at the National Hospital for Neurology and Neurosurgery, Queen Square, UK between August 2, 2017, and April 25, 2019, and for follow-up between April 6, 2022, and March 21, 2024.

At baseline, participants were required to be aged between 18 and 40 years old, capable of providing informed consent, and able to comply with study procedures. Participants in the Huntington's Disease Gene Expanded (HDGE) group at baseline were eligible with a CAG repeat length ≥40 and a Disease Burden Score (DBS) (age × (CAG – 35.5) of ≤240, estimating more than 18 years before clinical motor diagnosis[1,2]. Participants were excluded if they had significant comorbidities, were at-risk due to genetic status, or had reduced penetrance CAG repeat lengths (36-39 repeats). Control participants were stratified into three subcategories: genotype negative, family control, and community control. Genotype negative controls included individuals with a first or second-degree relative affected by HD, who had undergone predictive genetic testing for HD and were confirmed not to carry the HD expansion mutation, defined by a CAG repeat length of <36. Family controls comprised individuals who were not biologically related to HDGE, such as partners or spouses. Community controls were individuals not biologically related to HDGE and with no family history of HD. At follow-up, 57 out of 64 participants (89.1%) from the HDGE group returned, while 46 out of 67 participants (68.7%) from the control group returned. Among the 28 participants from baseline who did not return, reasons included 17 participants lost to follow-up, eight citing personal circumstances, two undergoing IVF/pregnancy, and one relocating abroad. At follow-up, participants were required to be capable of providing informed consent, and able to comply with study procedures, adhering to the same inclusion criteria as at baseline[3].

To account for participant dropout at follow-up, 58 potential new participants were considered for screening. New participants were recruited via the multidisciplinary HD clinic at the National Hospital for Neurology and Neurosurgery, Queen Square, and through engagement with the Huntington's Disease Youth Organisation (HDYO) and

the community. Following successful eligibility screening, 23 new participants were recruited at follow-up, comprising nine in the HDGE group and 14 in the control group.

Extended Data Figure 1 summarises the total cumulative assessments conducted, **Extended Data Table 6** lists all the outcomes, and **Supplementary Table 5** provides the number of assessments by modality, highlighting the completeness and scope of data collection throughout the study.

Clinical assessments

Clinical information, including participant eligibility, demographics, comorbidities, pharmacotherapy, non-pharmacologic therapies, nutritional supplements, and disclosure of recreational drug use, was collected. Prior to the lumbar puncture (LP) for cerebrospinal fluid (CSF) collection, participants underwent a neurological examination, including fundoscopy, and a general systems physical examination to rule out contraindications for proceeding with the LP. Clinical information relating to the LP, review of CSF cell counts post-LP, and records of telephone calls to participants 48 to 78 hours post-LP to monitor for adverse events were documented in the Electronic Patient Record system, Epic, which is the system employed by University College London Hospitals NHS Foundation Trust.

Functional and motor assessments were performed using the Unified Huntington's Disease Rating Scale (UHDRS), developed by the Huntington Study Group, having undergone rigorous reliability and validity testing to support ongoing utility in longitudinal studies[4]. The scale assesses four domains related to HD: motor, cognition, behaviour, and functional capacity. Motor signs were assessed using the UHDRS Total Motor Score (TMS). Comprising 31 items scored from grade 0 (normal) to grade 4 (most severe impairment), the total score ranges from 0 to 124. The UHDRS TMS assesses various motor features of HD, including ocular pursuit movements, saccade initiation and velocity, speech, tongue protrusion, coordination, bradykinesia, tone, the presence of chorea and/or dystonia, gait, and postural reflexes. Within the assessment of the UHDRS TMS, a Diagnostic Confidence Level score is assigned, graded from 0 (normal) to 4 (≥99% confidence, denoting unequivocal signs of motor abnormalities associated with HD)[4].

Independence was assessed using the UHDRS Total Functional Capacity (TFC). The TFC assesses the severity of limitation in functional capacity in HD across five domains, including occupation, finances, domestic chores, activities of daily living and the level of care required. The total TFC score ranges from 0 to 13, with 0 signifying total dependence for all care, and 13 indicating complete independence and full functional capacity[5]. Regarded as an extension of the TFC, the UHDRS Functional Assessment (FA) is a questionnaire comprising 25 items, which assesses the ability of an individual to perform specific tasks related to daily functioning. UHDRS FA scores range from 0 to 25, and the rater scores independence, ranging from 10% (tube-fed, total bed care) to 100% (no special care needed)[4].

Cognitive assessments

Cognitive testing was identical at baseline and follow-up with the exception of: a) the removal of the EMOTICOM Progressive Ratio Test due to participant feedback at baseline; and b) the introduction of the Goals Prior Assay. Additionally, Stroop interference was included within the core cognitive tasks at follow-up.

CANTAB Intra-Extra Dimensional Set Shifting (IED)
The IED **(Supplementary Figure 3a)** is a 7-minute test measuring cognitive flexibility and has similarities to a computerised version of the Wisconsin Card Sorting test. It initially features rule acquisition and reversal and then attentional set formation and set shifting. Participants are presented with two artificial dimensions including pink shapes and white lines. Through trial and error, the participant must select the correct rule. After six correct responses, the stimuli and/or rule changes. There are nine stages to the test. Initially the test presents simple stimuli with just one dimension (pink shapes). These later change to compound stimuli (white lines overlaid on pink shapes). Early in the test the shifts are intra-dimensional (ID) (pink shapes are relevant) to establish set formation. Then at stage eight a crucial extra-dimensional (ED) shift (white lines become relevant) occurs (attentional set-shifting). This latter stage is followed by a final reversal of the rule. Outcome measures include the number of pre-ED errors, ED shift errors, ED reversal errors and stages completed.

CANTAB One-Touch Stockings of Cambridge (OTS)
The OTS is a modified test of visuospatial planning and working memory based on the Tower of London which takes about 10 minutes to complete. Participants are shown example configurations of three coloured balls **(Supplementary Figure 3b)**. There are two displays and participants are asked the number of moves required to match their display to the example display without actually moving the balls. The problems differ in the number of moves required to match the example configuration, starting at one move progressing to six moves. The outcome measures include average response latency and number of problems solved efficiently at the first choice.

CANTAB Rapid Visual Information Processing (RVP)
The RVP is a 10-minute test which measures sustained attention by presenting a rapid stream of digits and requiring participants to detect target sequences. A white box is displayed in the centre of the screen in which digits 2-9 are rapidly presented at 100 digits per minute **(Supplementary Figure 3c)**. Participants are required to detect target sequences (e.g. 2-4-7, 3-5-7 or 4-6-8) and respond to this target sequence as quickly as possible. Outcome measures include A', a signal detection theory measure of target sensitivity, and mean response latency.

CANTAB Stop Signal Test (SST)
The SST is a test of response inhibition (impulse control) and takes 20 minutes to complete. The participant is shown an arrow in the centre of the screen and must respond with a button depending on the direction the arrow is pointing (left or right); **(Supplementary Figure 3d)**. If an audio tone is presented together with the arrow, the participant must withhold making the response (inhibition). The outcome measures are stop signal reaction time (SSRT), mean reaction time on go trials and the proportion of successful stops. The 'last-half' default setting was used, which calculates the SSRT from the last half of the trials.

CANTAB Paired Associates Learning (PAL)
The CANTAB PAL **(Supplementary Figure 3e)** is an 8-minute test assessing visuospatial memory and learning. Boxes are displayed on the screen in a spatial array and opened in a random order. One or more boxes contain a visual pattern. The patterns are subsequently displayed one by one in the middle of the screen and the

participant must select the box in which the pattern was previously presented. If the participant makes an error, the boxes are opened in the same order again. This is to remind the participant of the locations of the patterns before they attempt to remember again. The primary outcome measure is the total number of adjusted errors, which accounts for errors made and plus errors for stages that were not completed.

CANTAB Spatial Working Memory (SWM)
The CANTAB SWM **(Supplementary Figure 3f)** is a 9-minute test assessing spatial working memory. Test performance requires the retention and manipulation of visuospatial information. Coloured boxes are shown on screen and participants must select a box with a token. The token is stored on the edge of the screen and will not appear in the same location for the rest of the trial. Therefore, returning to the same location on the next search is an error. The colour and position of the boxes used are changed from trial to trial to discourage the use of stereotyped search strategies. The outcome measure is the total number of between-search errors.

The EMOTICOM battery was designed to measure aspects of social and emotional cognition and motivation[6]. We employed three subtests from the battery designed to assess emotion processing, motivation, and social cognition.

EMOTICOM Emotional Intensity Face Morphing
This task assesses the emotional intensity threshold at which participants recognise a facial emotion. During the 10-minute task, participants are shown faces where the emotional expression either increases or decreases in intensity. Participants are required to respond when they either first see the emotion (increasing) or can no longer see the emotion (decreasing). The outcome measures were average detection threshold of sad faces, for the increasing and decreasing condition separately.

EMOTICOM Moral Judgment Test
The 20-minute moral judgment test **(Supplementary Figure 4)** presents participants with cartoon depictions of moral scenarios. After each scenario, participants are asked to rate their levels of guilt, shame, annoyance, and feeling “bad”. Half of the cartoons are portrayed as deliberate harm and half as unintended harm. Participants were asked to rate their emotions from the perspective of both the victim and the perpetrator. The main outcome measure defined for this study was the guilt score collapsed across all conditions (deliberate vs. unintentional and perpetrator vs. victim).

Semantic Verbal Fluency
The verbal fluency test is a short test of semantic verbal retrieval. Participants are asked to list as many items as possible in a particular category (animals) in 60 seconds. The outcome measure is the number of words recited correctly.

Stroop Word Reading Test
In this test, participants are asked to read the words (colours) on the page as quickly possible. The outcome measure was the number of words read within 45 seconds.

Stroop Colour Naming Test
In this test, participants are asked to name the colours on the page as quickly as possible. The outcome measure was the number of colours named within 45 seconds.

Stroop Interference Task
In the Stroop Interference task[7] participants are presented with a list of words that represent colours, such as "red," "blue", "green", etc. Each word is printed in a colour that does not match the meaning of the word. For example, the word "red" is printed in blue ink. Participants are asked to name the colour of the ink in which each word is printed as quickly and accurately as possible, rather than reading the word itself. The participant has 45 seconds to read as many words correctly as possible. This test has shown impairments in HD[8].

Symbol Digit Modalities Test (SDMT)
The SDMT is a short test of psychomotor speed. It involves a substitution task, whereby using a reference key, the participant has 90 seconds to pair specific numbers and geometric figures.

Goals Prior Assay Task
Participants completed the 'Goal Priors Assay' task[9] which evaluates the mechanisms of apathy. The task is to land a virtual ball on a target **(Supplementary Figure 5)**. Participants press a force pad for 3 seconds and the force applied determines the initial velocity of the ball and therefore how far it travels in the direction of the target. The ball decelerates linearly towards the target. On a pseudo-random subset of trials, the ball disappears shortly after the button release, and participants are asked to estimate its final position. To indicate where they believe the ball to have reached, they refer to a numbered grid on screen. Each participant completes four blocks of the task which are varied by reward (points vs. no points) and effort (low force for a close target vs. high force for a far target). From comparison of estimation error vs. performance error, the precision of priors (expectation) on the outcome of actions is estimated, as a determinant of apathy. This task is novel to HD and is the first time it is used in this population, however, previous studies have shown evidence of apathy in HD[10], and the impact of apathy on task performance in Parkinson's disease and healthy adults[9,11].

Neuropsychiatry
To evaluate the prevalence of neuropsychiatric symptoms, the following additional assessments were administered:

Zung Self-Rating Depression Scale (SDS)
The SDS[12] is a self-rated scale that is a well-validated screening tool for depression. It covers affective, psychological, and somatic symptoms associated with depression. Items are scored on a four-point scale.

Spielberger State/Trait Anxiety (STAI)
The STAI[13] is a well-validated and commonly used self-report measure of anxiety, providing assessment of both state and trait levels of anxiety. Items are scored on a four-point scale that reflects frequency of anxious thoughts and behaviours.

Barratt Impulsivity Scale (BIS-11)
The BIS[14] is a self-report questionnaire designed to assess the personality/behavioural construct of impulsiveness. It measures three factors, including attentional, motor, and non-planning impulsivity. Items are scored on a four-point scale relating to frequency of behaviours.

Frontal Systems Behavioural Scale (FrSBE)

The FrSBE[15] provides a brief but reliable and valid measure of three frontal systems behavioural syndromes: 1) apathy; 2) disinhibition; and 3) executive dysfunction. The FrBSE is a 46-item behaviour rating scale. It includes a total score and scores across the three subscales, where 14 items relate to apathy, 15 items to disinhibition, and 17 items to executive dysfunction.

Obsessive-Compulsive Inventory (OCI-R)

The OCI[16] is a brief self-report instrument to determine severity of obsessive and compulsive behaviours. The items are scored on a five-point scale identifying how often an individual is distressed by behaviours relating to washing, checking, ordering, obsessing, hoarding, and neutralising.

Apathy Motivation Index (AMI)

Apathy is a disorder of motivation characterised by reduced action initiation and goal-directed behaviour. The AMI[17] is a recently developed (adapted from the Lille Apathy Rating Scale[18] apathy scale assessing three distinct subtypes of apathy: 1) behavioural activation; 2) social motivation; and 3) emotional sensitivity. The items are assessed on a five-point scale that represents how true each statement is.

Pittsburgh Sleep Quality Index (PSQI)

The PSQI[19] is a self-report questionnaire assessing several sub-categories including subjective quality of sleep, sleep onset latency, sleep duration, sleep efficiency, presence of sleep disturbances, use of hypnotic-sedative medication, and presence of daytime sleepiness.

MOS 36-Item Short-Form Health Survey (SF-36)

The SF-36[20] is a self-report scale assessing eight health concepts: 1) limitations in physical activities because of health problems; 2) limitations in social activities because of physical/emotional problems; 3) limitations in usual role because of physical health problems; 4) bodily pain; 5) general mental health (psychological distress and well-being); 6) limitations in usual role because of emotional problems; 7) vitality (energy and fatigue); and 8) general health.

Neuroimaging

Image acquisition

All MRI data were acquired at the Wellcome Centre for Human Neuroimaging using a 3-Tesla (3T) Prisma scanner (Siemens Healthcare, Germany) with radiofrequency body coil for transmission and a 64-channel head coil for signal reception using a protocol optimised for this study. Imaging protocols were identical at baseline and follow-up and there were no hardware or major software upgrades to the scanner over the 4.5-year time interval.

The T1-weighted (T1w) images were acquired using a 3D Magnetisation Prepared Rapid Gradient Echo sequence with the following parameters: repetition time (TR) = 2530 ms; time to echo (TE) = 3.34 ms; inversion time = 1100 ms; flip angle = 7°; field of view = $256 \times 256 \times 176$ mm^3 and a resolution of $1.0 \times 1.0 \times 1.0$ mm^3.

Diffusion weighted images (DWI) were acquired using a multiband spin-echo echo planar imaging (EPI) sequence with time-shifted radiofrequency pulses with acceleration factor 2, TR = 3260 ms, TE = 58 ms, flip angle = 88º, field of view = 220×220 mm^2, with 72 slices collected at a resolution of 2×2×2 mm^3. The multi-shell data consisted of b-values of 0 (n=10), 100 (n=8), 300 (n=8), 1000 (n=64) and 2000 (n=64) s/mm^2.

The Multiparametric Mapping (MPM) protocol consisted of three differently weighted 3D multiecho Fast Low Angle Shot acquisitions: Magnetisation Transfer weighted (MTw), Proton Density weighted (PDw) and T1w in addition to two scans collected to estimate participant-specific field inhomogeneities. The MTw, PDw and T1w scans were all acquired using a field of view of 256×224×179 mm^3, TR = 25 ms, flip angle of 6º and resolution of 0.8×0.8×0.8 mm^3. To improve image quality, i.e. maximise signal to noise ratio and minimise geometric distortion, eight gradient echoes from 2.34-18.44 ms were acquired for the PDw and T1w images and six from 2.34-13.84 ms for the MTw image. B1 Transmit bias field maps were collected using a 3D EPI acquisition of spin-echo and stimulated echo images. 48 slices, TR = 500ms, TE1 = 39.06, TE2 = 130 ms, slice thickness = 4 mm; field-of-view = 256×192×192 mm^3. Finally, the field maps were acquired with 64 slices using TR = 1020 ms, TE1 = 10 ms TE2 = 12.46 ms, slice thickness = 4 mm; field-of-view = 192×192 mm^3. Parallel imaging acceleration was used, and 3D distortion correction was applied to all images.

Baseline imaging was reprocessed in parallel with follow-up scans for all image analysis to ensure consistency of software pipelines and remove any potential for systematic bias between timepoints.

Volumetric MRI
T1w images were bias corrected using N3 bias correction[21] within the Medical Imaging Display and Analysis System (MIDAS) software tool[22]. Whole brain, total intracranial and ventricular volumes were delineated using MIDAS with semi-automated protocols[23,24]. Putamen and caudate volumes were derived using the Multi Atlas Label Propagation with Expectation Maximisation-Based Refinement (MALP-EM) software[25] (version 1.2; https://github.com/ledigchr/MALPEM), after linear registration into standard space using the ICBM 152 template (http://www.bic.mni.mcgill.ca/ServicesAtlases/ICBM152NLin2009). Voxel-based morphometry[26] (Statistical Parametric Mapping version 12; https://www.fil.ion.ucl.ac.uk/spm/software/spm12/) was used with the Computational Anatomy Toolbox (version 12; http://www.neuro.uni-jena.de/cat12/CAT12-Manual.pdf) to generate grey and white matter volumes in native space.

Longitudinal change was estimated using the boundary shift integral (BSI) for whole brain, ventricles, and caudate using established methods[22,27]. Change within the putamen was estimated by subtraction of the baseline and follow-up volumes generated by MALP-EM. Non-linear fluid registration[28] was used to derive within-subject voxel-compression maps which were convolved with individual baseline VBM-derived grey and white matter maps to determine change within those tissues[29].

Diffusion weighted imaging

DWI images were corrected for susceptibility-induced artefacts using topup, and for motion and eddy-current induced artefacts using eddy[30–32] both from the FMRIB Software Library (FSL) (version 5.0.11; https://fsl.fmrib.ox.ac.uk/fsl/fslwiki). Diffusion tensors (DT) were fitted to the artefact-corrected DWI data using FSL dtifit. DT indices, including fractional anisotropy (FA), mean diffusivity (MD), axial diffusivity (AD), and radial diffusivity (RD), were then calculated using DTI-TK (version 2.3.3; http://dti-tk.sf.net). The Neurite Orientation and Dispersion Density (NODDI) model[33] was fitted to artefact-corrected DWI data using the Accelerated Microstructure Imaging via Convex Optimisation toolbox (version 10a65b0, Nov 2016; http://amico.sourceforge.net/)[34]. NODDI distinguishes between markers of axonal density and axonal spatial organisation, while removing the potentially confounding effect of free water. Output indices were Neurite Density Index (NDI), Orientation Dispersion Index (ODI), and Free Water Fraction (FWF). Six pre-specified white matter regions of interest (ROIs) were used: genu, splenium and mid-body of the corpus callosum, anterior and posterior limbs of the internal capsules, and the external capsules, as defined in the Johns Hopkins University (JHU) white matter atlas[35]. For the internal and external capsules, the left and right hemispheric regions were combined as one. The linear and non-linear deformation in DTI-TK[36] was used to create a bootstrapped study-specific population template, which was transformed into JHU space. Individual diffusion images were aligned with the JHU space and all ROIs were visually quality controlled. The average values of DT and NODDI indices were extracted for each ROI in each participant[37]. Tissue-weighted averaging was applied to NODDI metrics to reduce estimation bias[38]. Change over time was determined by subtraction of follow-up from baseline value.

Structural connectivity

Structural connectivity analysis was performed for 103 baseline and 90 follow-up participants. Only right-handed participants were included to avoid confounding effects caused by differences in structural connectivity in those who are right-hemisphere dominant. See **Supplementary Figure 6** for analysis pipeline.

Seventy-six cortical ROIs were segmented on the T1w images using FreeSurfer (version 7.2.0; https://surfer.nmr.mgh.harvard.edu/)[39]. The cerebellum was not included as diffusion data was incomplete. Tissue partial volume maps of the brain white matter, grey matter, and CSF were prepared for anatomically constrained tractography (ACT)[40]. Previous studies have demonstrated that cortical rich club (hub regions that have the highest number of connections to other brain regions in the network) and striatal structural connections are selectively vulnerable in gene carriers closer to expected disease onset, with an accompanying loss of integration and increased segregation across the brain network[41,42]. The striatum was segmented using an atlas that splits the structure into three sub-regions in each hemisphere according to cortical-striatal anatomical connections[43]. These subregions are labelled limbic, executive, and sensorimotor based on the dominant cortical connectivity to each striatal subregion. A surface-based registration was used to register the atlas to participant T1 space before registering to participant diffusion space using the NiftyReg toolkit[44]. All diffusion processing steps were conducted using commands either implemented within MRtrix3 (version 3.0.4; www.mtrix.org), or using MRtrix scripts that interfaced with external software packages.

DWI were processed as above and fibre orientation distributions (FODs) were computed using multi-shell, multi-tissue constrained spherical deconvolution with group averaged response functions for white matter, grey matter, and CSF[45]. Multi-tissue informed log-domain global intensity normalisation was then performed. Whole-brain probabilistic tractography was performed in participant-space using the 2nd order integration over FODs (iFOD2) algorithm with a FOD threshold of 0.06. Ten million streamlines were generated for each scan using dynamic seeding in the white matter. The 'back-tracking' mechanism was used within the ACT framework[40] to allow tracks to be truncated and re-tracked if poor structural termination was encountered.

Connectomes were constructed by combining streamline tractograms with the participant's grey matter parcellation. Streamlines were assigned to the closest node within a 2 mm radius of each streamline endpoint[46]. Structural connections were weighted by streamline count and a cross-sectional area multiplier, as implemented in Spherical-Deconvolution Informed Filtering of Tractograms 2 (SIFT2)[46]. Connections were then combined into 82×82 undirected and weighted matrices. SIFT2 was chosen in preference to SIFT as it can retain the full connectome and requires significantly less processing time.

Graph metrics were calculated using the brain connectivity toolbox (version 2019-03-03)[47] and have been detailed elsewhere[48]. We measured connection strength of the 6 striatal regions, 12 cortical rich club regions, and whole brain network measures of modularity and global efficiency. The strength of each connection is calculated by the sum of its connection weights. The 12 cortical rich club regions selected were those with the highest connection strength in the network. These were the superior frontal, precentral, superior parietal, thalamus, inferior parietal and rostral middle frontal regions from both hemispheres. The rich club regions were the same for HDGE and controls and consistent with previous literature[40,49]. Whole brain connectivity was assessed by using measures of modularity and global efficiency. Average path length represents the average of shortest paths between brain regions in a network. Global efficiency is the inverse of the average shortest path length and a decrease represents loss of network integration. Modularity refers to the community structure within brain networks. Modules are clusters of nodes with dense interconnectivity within the cluster but sparse connections between nodes in different clusters. As modularity increases, the network is more segregated with fewer connections between different modules.

Change over time was estimated by subtraction of the follow-up from baseline values.

Multiparametric mapping
The MPM multi-echo protocol was created to estimate the longitudinal relaxation rate (R1), the effective transverse relaxation rate (R2*) the proton density (PD), and magnetisation transfer (MT)[50]. Data collection involved the acquisition of at least six images at different TE lengths for each of the PD, T1, and MT weighted acquisitions. These images were pre-processed to generate PD, R1, MT, and R2* quantitative maps[50]. Additional B1 and Field Maps were collected to correct for field inhomogeneities. R1 represents estimates of iron and myelin, R2* iron, MT myelin and PD sensitivity to water content. Scans were first converted to NIfTI format, and visual quality control performed. Pre-processing was then performed within the histology MRI (hMRI) toolbox version 0.6.1[51], using default settings with the exceptions of (1) calculation of R1 and PD did not use small angle approximation, (2) R2* fitting was

computed with WLS3, and (3) imperfect spoiling correction was enabled. This was run within the Statistical Parametric Mapping software (SPM version 12) in MATLAB version R2022b (The Mathworks Inc, Natick, MA, USA). RF sensitivity bias correction calculated via Unified Segmentation, and B1 sensitivity bias correction calculated via the RF transmit (B+1) and receive (B−1) field measurements. Quantitative maps were then calculated from the three multi-echo spoiled gradient echo scans; these were visually examined after preprocessing.

Eight ROIs were specified *a priori*, the six white matter ROIs from the JHU described above for diffusion imaging plus the caudate and putamen generated by MALP-EM. The caudate and putamen ROIs were registered and resampled to native MPM space for each participant via an affine registration using NiftyReg software (NifTK version 12.11)[44]. These regions were then binarised and eroded by one voxel in all planes to ensure that they contained no CSF or white matter. The JHU regions were registered and resampled to native MPM space for each participant via affine transformation followed by non-linear registrations (NifTK version 12.11). All masks were overlaid on the MPM maps and visually checked to ensure successful registration. Quantitative values representing the average value within each region were then extracted for each quantitative map, for every participant for both timepoints. Change over time was estimated by subtraction of the follow-up from baseline values.

Imaging quality control

In addition to checking consistency of acquisition parameters, all raw images from each modality were visually inspected, blinded to genetic status, to ensure appropriate brain coverage and to identify artefacts, e.g. motion. The image processing workflow outlined above involves multiple steps and further visual inspection was performed throughout the processing, including the final images produced. In particular, when scans were co-registered to atlas regions, great care was taken to ensure appropriate positional matching was achieved. Quality control was performed by experienced image analysts (RIS, NZH, ML) and where there was any uncertainty about image acceptability, scans were reviewed by all analysts to reach a consensus.

Supplementary Table 5 outlines the scans which passed quality control for each modality. All raw volumetric scans passed quality control, and all resulting regions derived from MALP-EM (caudate and putamen), MIDAS (whole brain, ventricles) and VBM (grey and white matter) were accepted, as well as the direct measures of change including the BSI and non-linear fluid registration.

At baseline one control did not undergo DWI scanning and one control was excluded due to a preprocessing failure. Three HDGE participants were excluded due to motion artefact. At follow-up one control did not undergo DWI imaging. One control was excluded due to a preprocessing failure and three participants (two HDGE, one control) were excluded due to motion artefact.

Structural connectivity used the DWI scans so participants outlined above were excluded with the exception of the two controls who failed the diffusion-specific preprocessing. In addition, only right-handed participants were included in this analysis which led to the exclusion of a further 14 participants (seven HDGE, seven controls) at baseline and 20 participants (10 HDGE, 10 controls) at follow-up.

For MPM acquisition, at baseline, two participants (one HDGE, one control) did not undergo MPM scanning due to claustrophobia. Six participants were excluded from the MPM analysis, two due to motion (one HDGE, one control) and four HDGE due to technical processing failure. There were an additional three partial fails due to motion (three controls), where only the R2* values were excluded from the analysis. At follow-up six participants (four HDGE, two controls) did not undergo or had incomplete MPM acquisitions due to scanner breakdown, anxiety or technical issues. Five participants failed MPM quality control due to either motion (three HDGE, one control) or technical processing failure (one control). There were an additional seven partial fails due to montion on one or more of the MPM maps (three HDGE, four controls).

Biofluids

Regarding platform and provider changes at follow-up compared to baseline, the Neurology 4-Plex A assay transitioned from the SIMOA HD-1 Analyzer at baseline to the SIMOA HD-X at follow-up. At baseline, intereukin-6 (IL-6) and IL-8 levels in CSF were measured using the SIMOA HD-1 Analyzer, but at follow-up, these cytokines were measured using both baseline and follow-up CSF samples on the Human IL-6 and Human IL-8 assays via the V-PLEX Meso Scale Discovery (MSD) platform. At baseline, CSF mHTT was quantified with the 2B7-MW1 immunoassay on the Single Molecule Counting (SMC) Erenna platform by Evotek. However, due to the current unavailability of this platform, CSF mHTT was measured using the SMCxPRO platform performed by IRBM at follow-up. See **Supplementary Table 19** for assay details.

Total huntingtin (tHTT) was not quantified at follow-up as this was an exploratory biofluid measure at baseline. The presence of the mutant *HTT* gene did not influence tHTT levels in CSF, with no significant difference observed between control and the HDGE groups at baseline[3].

Additionally, neurogranin, a marker of synaptic function, was not remeasured in CSF at follow-up, as there was no significant difference in CSF neurogranin levels between control and HDGE groups at baseline[3], which remains consistent with findings from a previous HDGE group[52].

Novel to this timepoint, PENK, a surrogate marker for striatal medium spiny neuron (MSN) state, was measured as an exploratory fluid biomarker beyond the primary fluid biomarkers specified in the pre-defined statistical analysis plan (ClinicalTrials.gov: NCT06391619). Unbiased liquid chromatography-mass spectrometry (LC-MS) based proteomics analysis was performed using the tandem mass tag (TMT) technique[53] to measure relative abundance of CSF PENK.

CSF samples were prepared with the addition of an initial multi-affinity depletion step to reduce interference by high-abundant blood derived proteins. Following this step, samples were subjected to reduction and alkylation of cysteine residues, digestion with trypsin and endoproteinase Lys-C, and isobaric labelling using TMTpro 18-plex reagents[55] (Thermo Fisher Scientific). TMT multiplex peptide samples were fractionated by high-pH reversed-phase high-performance LC (HPLC)[56,57], and analysed by nano-HPLC (EasyLC, Thermo Fisher Scientific) coupled to a high-resolution Orbitrap hybrid mass spectrometer (Orbitrap Lumos Tribrid, Thermo Fisher

Scientific). Protein identification and data processing for quantification was performed using Proteome Discoverer 2.5 (Thermo Fisher Scientific), and R Statistics.

Proteomics – for quantification of relative abundance of PENK
CSF sample aliquots (75 µl) were mixed with 75 µl resuspended depletion resin (High-Select Top14 Abundant Protein Depletion Resin (A36372), Thermo Fisher Scientific) in 96-well filter plates (AcroPrep Advance, 350 µl, 0.45 µm Supor membrane (518-0023), Cytiva Life Sciences) and incubated on a shaking table (900 RPM) at room temperature for 45 min. The flowthrough was collected in a polypropylene PCR plate (Armadillo, Thermo Fisher Scientific) by centrifugation at 1000 × g for 5 min. All reagents were added to the samples using a microfluidic liquid dispenser (Mantis, Formulatrix). Aliquots (10 µl) of a solution of 6.4% sodium deoxycholate (w/v) and 640 mM TEAB were added to the samples and the sample plates were sealed with cap strips, placed on a shaking table (2500 RPM) for 30 s, and incubated for 1 h at 56 °C in an oven to reduce cystines. After the sample plate had been let to cool to room temperature, 5 µl 266 mM freshly prepared iodoacetamide solution were added to the samples. The sample plates were placed on a shaking table (2500 RPM) for 30 s and incubated for 30 min in the dark, at room temperature, to carbamidomethylate cysteine residues. Aliquots (5 µl) of a mixture of trypsin and endoproteinase Lys-C (0.3 µg/µL, Trypsin/Lys-C Mass Spec Grade (V5072) Promega) were added to the samples. After sealing and shaking the plates as above, the plates were sealed and incubated over night at 37 °C. TMTpro reagents (Thermo Fisher Scientific) were dissolved in acetonitrile (25 µg/µl) and 10 µl were added to the samples. The plates were sealed and placed on a shaking table (900 RPM) for 1 h. The labelling reaction was quenched by adding 5 µl 6.15% hydroxylamine (v/v) and incubating on a shaker (900 RPM) for 30 min, after which the differently labelled samples were combined into 18-plex TMT sets. The samples were acidified by adding 550 µl 1 M HCl, and 0.1% TFA were added to increase the sample volume to 6 mL, necessary to decrease the acetonitrile concentration to 3%, to avoid losing peptides in the subsequent desalting step. The samples were desalted by solid phase extraction (Sep-Pak C18 (WAT023590), Waters) using a vacuum manifold. A solution of 0.1% trifluoroacetic acid (TFA) was used for washing (2 * 1 mL), and samples were eluted using 1 mL 80% acetonitrile (v/v), 0.1% TFA.

For peptide fractionation by reversed-phase HPLC at basic pH, the samples were redissolved in 22 µL 2.5 mM ammonium hydroxide and loaded on the HPLC (Ultimate 3000, Thermo Fisher Scientific) equipped with an XBridge BEH C18 column (pore size: 130 Å, inner diameter: 4.6 mm, Waters). The following gradient was employed for peptide elution: Buffer B ranging from 1-45% over a 65 min gradient (flow rate 100 µL/min), Buffer C=10% (solvent A: H2O, solvent B: 84% acetonitrile, solvent C: 25 mM ammonium hydroxide). Fractions were collected at 1 min time intervals circling over two rows in a 96-well microtiter plate, resulting in 24 concatenated fractions. The column was then cleaned at 90% B, 10% C for 10 min and subsequently equilibrated at 1% B, 10% C for 10 min. Fractions were dried by vacuum centrifugation and stored at -20°C pending LC-MS analysis.

LC-MS
The fractions were analysed on an Orbitrap Lumos Tribrid mass spectrometer equipped with a FAIMS Pro ion mobility system and interfaced with an Easy-nLC 1200 liquid chromatography system (all Thermo Fisher Scientific). Peptides were trapped

on an Acclaim Pepmap 100 C18 trap column (100 µm × 2 cm, particle size 5 µm, Thermo Fisher Scientific) and separated on an in-house packed analytical column (35 cm × 75 µm, particle size 3 µm, Reprosil-Pur C18, Dr. Maisch) using a stepped gradient from 5% to 35% B over 77 min at a flow of 300 nL/min (solvent A: 0.2% formic acid, solvent B: 80% ACN, 0.2% formic acid). The mass spectrometer was operated in the positive ion mode, using data-dependent acquisition. Alternating MS/MS cycles were performed (cycle time=1.5 s) at compensation voltages -50 V and -70 V. First, a full Orbitrap MS scan was recorded (R = 120 k, AGC target = 100%, max injection time = 50 ms), followed by data dependent Orbitrap MS/MS scans (isolation window = 0.7 m/z, activation type = HCD, R = 50 k, AGC target = 200%, max. injection time = 120 ms).

LC-MS data processing

Proteome Discoverer Version 2.5 (Thermo Fisher Scientific) was used for protein identification and quantification. Peak integration for reporter ion quantification was performed with the integration method of most confident centroid (integration tolerance = 20 ppm). Peptides were identified using SequestHT search engine with UniProtKB Swiss-Prot (TaxID = 9606, Homo sapiens) set as database. The search parameters included precursor Δm tolerance = 5 ppm, fragment Δm tolerance = 0.02 Da, missed cleavages = 2, min. peptide length = 6, fixed modifications= carbamidomethyl (Cys), TMTpro (peptide N-terminus, K residues). The Percolator algorithm was used for peptide scoring with an identification threshold of 1% False Discovery Rate (FDR) on the protein level. For quantification, peptide groups were considered based on their uniqueness (unique peptides) and in accordance with the principle of parsimony (razor peptides). Missing values were not imputed. Protein ratios were calculated by dividing protein abundances in each study sample TMT channel with the abundance in the global internal standard channel (TMT channel 135N). Data normalisation was then performed by dividing each protein ratio by the respective sample median using R Statistics.

Statistics

False Discovery Rates were calculated separately per measurement domain (cognitive, volumetric imaging, bioassays, etc.) to provide conceptually and technically sensible sets of underlying *P* values. Note that this is a relevant consideration for the appropriate application of FDR, whereas the total number of comparisons simultaneously considered is not.

The Goals Prior Assay and Stroop interference tasks were introduced at follow-up and tested by conventional linear regression rather than the mixed-effect linear models with a participant random effect used for longitudinal data.

To assess potential causal implications of somatic expansion ratio (SER)-biomarker associations, we compared the statistical strength of SER as a predictor of the biomarker before and after controlling for age and CAG length **(Extended Data Figure 4)**. A substantially weakened SER relationship after age and CAG control would suggest that the associations were due to mutual influence of CAG length over time without more direct causality. Conversely, we assessed the strength of the age-CAG versus biomarker relationships with and without SER control. Weakened age-CAG associations in the presence of a significant predictive SER effect would be consistent

with an intermediate causal role for CNS somatic expansion (as indirectly assessed by SER in white blood cell DNA).

We used the same regression methods as for the somatic expansion models to assess associations between log NfL levels and volumetric outcomes. However, we attempted no causal interpretation of NfL versus volumetric relationships. Although these are both HD-related biomarkers there is no well-justified conception of one of these changes "causing" the other.

Supplementary Results and Discussion

Participant characteristics

Out of 131 baseline participants, 108 returned for follow-up. Retention was 89% in the HDGE group and 69% in controls. Dropout was influenced by the long interval between visits and a young mobile cohort who had significant changes in circumstances including childcare responsibilities. There was greater dropout in controls, which, in some cases was due to relationship breakups, since many controls were unaffected partners of the HDGE group. In the HDGE group there was slightly greater dropout in females (15% compared with 10%) due to pregnancy and undergoing preimplantation genetic testing via IVF.

Participants were recruited from across the UK and the study was limited by severe transport strikes (35 UK rail and 6 London underground strikes), delaying the final visit from September 2023 to March 2024.

Clinical

There was a significant difference between the HDGE group and controls in the UHDRS TMS (P=5x10^{-5}). However, the mean score for the HDGE group was 0.8 and individuals with a TMS of <6 are considered not to exhibit motor signs typical of HD[58]. The TMS has poor inter-rater reliability [59] and there were a total of six clinical raters across the two timepoints. For that reason, we do not have confidence in this finding and do not believe there is any clinically meaningful difference in motor performance between the two groups.

Cognitive

Cross-sectional differences between the HDGE group and controls were found in the CANTAB SST (*P*=0.01; FDR=0.13) **(Supplementary Table 3 and Supplementary Figure 1)**, RVP A' (*P*=0.03; FDR=0.15), and semantic verbal fluency (*P*=0.04; FDR=0.15). There were no cross-sectional associations with age and CAG. There were some longitudinal associations with age and CAG. Specifically, PAL and SDMT scores decrease with increasing values of age-by-CAG interaction, and RVP scores decrease with age and age-by-CAG interaction. There was no significant additional contribution from the main effect of CAG length outside the age interaction.

There were no other notable associations between any of these measures/outcomes and influence of age, CAG and age-by-CAG interaction (all FDR>0.15).

We employed an extensive battery of neuropsychological tests, and after controlling for multiple comparisons, we found poorer performance in the HDGE group for the

CANTAB SST, CANTAB RVP and semantic verbal fluency. Previous studies have shown early changes in verbal fluency in HD[60]. Importantly, there were no differences between groups for the RVP mean latency, which indicates that this change is specific to attention and not simply a motor slowing. Notably, the RVP A’ measure showed both a cross-sectional group difference as well as a longitudinal association with the age and CAG interaction. In addition, at the baseline the RVP A’ showed some group differences, although these did not survive correction for multiple comparisons. Taken together, these findings suggest that there may be a subtle, but early impairment in sustained attention in HD.

Neuroimaging studies have shown that a frontal-parietal network including the inferior frontal gyrus is involved in RVP performance[61]. Importantly, the same region is involved in response inhibition and performance on the SST task[62]. Therefore, the early changes in both RVP and SST, indicates that the inferior frontal gyrus should be a focus for future research in early HD. These cognitive changes may also be related to increased impulsive behaviours and difficulty with concentration and thinking, which are often reported by individuals with HD[63]. It is also worth noting that both the RVP [64] and SST[65] are known to be affected in the neurodevelopmental disorder attention deficit hyperactivity disorder. Which may suggest that there is a component of early neural development being affected in HD. However, the age and CAG association in the RVP test does seem to suggest this is related to HD progression. Further longitudinal follow-up as planned may better elucidate the contribution of neurodevelopmental and neurodegenerative impairments.

Neuropsychiatric

Cross-sectionally, the HDGE group perceived a worse general health status compared to the control group (FDR=0.10), according to the SF-36 General Health questionnaire **(Supplementary Table 4 and Supplementary Figure 1)**. There were no other significant group differences. Importantly, there were no significant associations between any of the neuropsychiatric measures/outcomes and influence of age, CAG and age-by-CAG interaction (all FDR>0.15). This suggests that the SF-36 changes may not directly reflect a HDGE individual’s own disease status, but may instead reflect the familial and developmental context of HD.

Neuroimaging

Volumetric

There were five significant cross-sectional differences in volumetric measures between the HDGE and control groups: putamen (P=4.03×10^{-5}; FDR=2.42×10^{-4}); caudate (P=9.47×10^{-3}; FDR=0.029); whole brain (P=3.50×10^{-2}; FDR=0.070); white matter (P=9.33×10^{-2}; FDR=0.140); and grey matter (P=1.20×10^{-1}; FDR=0.144) **(Supplementary Table 8)**. All six volumetric measures reached FDR threshold for association with age, CAG, and age-by-CAG interaction: putamen (P=3.17×10^{-16}; FDR=1.66×10^{-15}); caudate (P=5.53×10^{-16}; FDR=1.66×10^{-15}); grey matter (P=1.39×10^{-4}; FDR=2.60×10^{-4}); whole brain (P=1.73×10^{-4}; FDR=2.60×10^{-4}); and ventricles (P=7.35×10^{-4}; FDR=8.82×10^{-4}). White matter significance was far lower than the other volumetric measures (P=0.138; FDR=0.138).

Group differences in longitudinal volumetric change and associations with age and CAG are described in the main manuscript and summary statistics are provided in **Extended Data Table 2**.

Diffusion weighted imaging
There were no cross-sectional differences between the HDGE and control groups in any of the diffusion metrics (FDR>0.18) **(Supplementary Table 9)**. There were no age and CAG associations (FDR>0.21).

Diffusion weighted imaging demonstrated significant longitudinal increases in mean diffusivity in HDGE compared with controls in all ROIs (all FDR<0.15) **(Extended Data Table 3)**. The HDGE group showed region-specific significant increases in radial and axial diffusivity (FDR<0.15) and decreases in fractional anisotropy in the splenium and mid corpus callosum (FDR<0.01). There was a significant disease-related reduction in neurite density index in all sub-regions of the corpus callosum (FDR<0.15) and the external capsule (FDR=0.10) whilst the free water fraction was elevated over time in HDGE compared with controls in the splenium (FDR=0.009) and mid corpus callosum (FDR=0.05). Orientation dispersion index was elevated in the anterior internal capsule (FDR=0.004) and the mid corpus callosum (FDR=0.07) in the HDGE compared with controls. All these changes in diffusion metrics are consistent with loss of microstructural integrity over time. The anterior internal capsule showed disease-related increases in fractional anisotropy (FDR=0.008) and orientation dispersion index (FDR=0.004).

Structural connectivity
There were no significant cross-sectional group differences in any of the structural connectivity measures (FDR>0.31) **(Supplementary Table 10)**. There were no significant age and CAG associations (FDR>0.20). Longitudinal metrics did not show any difference between the HDGE and control groups (FDR>0.43) **(Supplementary Table 6)** and there were no associations with age and CAG (FDR>0.15).

Multiparametric mapping
MPM data demonstrated significant cross-sectional differences between the HDGE and control groups only in the putamen where R1 was elevated in the HDGE group (P=0.003; FDR=0.102), suggestive of increased levels of iron. All other measures did not reach the significance threshold for FDR (FDR>0.25) **(Supplementary Table 11)**. There were associations with age and CAG in the following metrics: putamen R2s (P=0.003; FDR=0.082); putamen R1 (P=0.007; FDR=0.112); EC PD (P=0.014; FDR=0.112); CC posterior PD (P=0.017; FDR=0.112); and putamen PD (P=0.017; FDR=0.112).

There were no significant group differences in longitudinal change for any of the MPM metrics (FDR>0.20) and summary statistics are provided in **Supplementary Table 7**. There were no longitudinal associations with age and CAG (FDR>0.77).

Biofluids
Longitudinally, 53 of the HDGE group (93%) and 33 controls (72%) underwent a LP for CSF collection at follow-up. Among the 17 longitudinal participants who did not a LP at follow-up, reasons documented were declined (n=11), previous low-pressure syndrome after baseline (n=2), history of migraine (n=1), needle phobia (n=2), and scoliosis (n=1). Summary statistics for the longitudinal data are provided in

Supplementary Table 17 and boxplots of biofluids with non-significant longitudinal change are shown in **Extended Data Figure 2**.

There were four notable cross-sectional differences in fluid biomarkers between the HDGE and control groups: CSF NfL, CSF PENK, plasma NfL, and CSF-YKL-40 **(Supplementary Table 18)**. Cross-sectionally, log concentrations of both CSF NfL (P=5.37×10^{-30}; FDR=6.45×10^{-29}) and PENK (P=1.72×10^{-7}; FDR=1.04×10^{-6}) were highly associated with age, CAG length, and their interaction. There was also evidence for an influence on longitudinal change in CSF NfL (P=0.0269; FDR=0.322) and PENK (P=0.0547, FDR=0.328). Plasma NfL had a similar cross-sectional association (P=7.24×10^{-7}; FDR=2.90×10^{-6}) but no significant longitudinal association with age and CAG. These results controlled for age and sex effects in the controls and for the average log concentrations in the HDGE group. Regression coefficients are reported in **Supplementary Tables 12-14**.

There were no significant cross-sectional differences in CSF mHTT levels influenced by age, CAG length, or the age-by-CAG interaction in the HDGE group. The estimated effect per year of age was -0.0256 (SE=0.0166, Df=82.925, t=-1.548, P=0.125).

Longitudinal analysis revealed no significant differences regarding the influence of age, CAG length, and their interaction on CSF mHTT. Specifically, tests for age, CAG length, and age-by-CAG interaction were non-significant (P=0.390, 3 Df). Similarly, the test for CAG length and age-by-CAG interaction, controlling for age, was non-significant (P=0.300, 2 Df). The test for age effect without considering CAG length also showed no significance (P=0.227, 1 Df), indicating a non-significant decrease/year.

At this stage of the disease, CSF mHTT levels are very low. **Supplementary Figure 2** shows the frequency of outcomes from the CSF mHTT assay performance in 120 CSF samples from the HDGE group. The outcomes are categorised as follows: 33 samples were below the lower limit of quantification (<LLoQ), 1 sample was below the limit of detection (<LoD), 23 samples had an unacceptable coefficient of variation greater than 30% (CV% >30%), 9 samples showed inconsistency, 41 samples were quantifiable (green bar), and 13 samples resulted in run failure (grey bar). Each bar represents the number of samples (n) in each category.

Predictors

After controlling for CAG length, age, age-by-CAG, sex, and SER effects, compared to typical allele structure, the CAACAG CCGCCA loss atypical allele had significant effects on rates of caudate (P=1.90×10^{-5}) **(Manuscript – Figure 5b.i)**, putamen (P=0.007) **(Manuscript – Figure 5b.ii)**, grey matter (P=0.005), and whole brain (P=0.0049) atrophy. No other significant associations were observed between the typical allele and the CAACAG CCGCCA or CCGCCA loss atypical alleles, nor between the two atypical alleles themselves, in relation to other volumetric changes.

After controlling for CAG length, age, age-by-CAG, sex, and SER effects, compared to typical allele structure, the CAACAG CCGCCA loss atypical allele had significant effects on cross-sectional CSF NfL (P=0.002) **(Manuscript – Figure 5b.iii),** CSF PENK (P=0.001) **(Manuscript – Figure 5b.iv)**, plasma NfL (P=0.005); CSF GFAP

(*P*=0.029), and CSF UCH-L1 (*P*=0.013) levels. No other significant associations were observed between the typical allele and the CAACAG CCGCCA or CCGCCA loss atypical alleles, nor between the two atypical alleles themselves, in relation to other cross-sectional biofluid difference.

Supplementary Table 1: Longitudinal cognitive data

Outcomes	Control Mean	HDGE Mean	Estimate of Mean Difference	Df	Lower CL	Upper CL	*P* value	FDR
IED ED Shift Error	0.137	-0.333	-0.470	133	-1.177	0.238	0.191	0.577
IED ED Reversal Error	0.068	-0.110	-0.175	144	-0.604	0.253	0.420	0.577
IED Pre ED Error	-0.022	0.076	0.096	152	-0.157	0.394	0.454	0.577
OTS (Minimum Moves)	0.143	0.062	-0.081	140	-0.250	0.088	0.343	0.885
SDMT	0.065	-0.218	-0.283	124	-0.918	0.353	0.380	0.885
RVP 'A	1.48×10^{-3}	4.21×10^{-4}	-0.001	121	-0.004	0.002	0.424	0.885
IED Stage Completion	-0.030	-0.004	0.025	150	-0.064	0.114	0.577	0.577
SSRT	1.970	3.020	1.053	149	-3.249	5.355	0.629	0.885
Verbal Fluency (Total Correct)	0.510	0.416	-0.094	126	-0.487	0.299	0.635	0.885
Agent Guilt Score	0.010	-0.002	-0.012	138	-0.062	0.038	0.644	0.885
SWM (Between Errors)	-0.532	-0.005	0.527	127	-2.244	3.299	0.707	0.885
Intensity Face Morphing (negative)	1.210	1.180	-0.026	156	-0.279	0.227	0.839	0.885
PAL (Total Error Adjusted)	1.440	1.230	-0.207	133	-2.546	2.132	0.861	0.885
RVP (Mean Latency)	3.030	3.450	0.424	131	-5.370	6.217	0.885	0.885

Table showing adjusted mean longitudinal changes in cognitive outcomes comparing control participants to HDGE participants. Outcomes are arranged in ascending order of *P* value. Statistical analyses are based on mixed-effect linear models with a participant random effect. All models are controlled for age, sex, and age-by-sex interaction. These cognitive models also controlled for ISCED education level and estimated IQ via the NART score. Statistical two-sided group comparisons were adjusted for multiple comparisons using the FDR, with *P* values, degrees of freedom, and confidence limits provided in the table.

Abbreviations:
CL=Confidence Limit. Df=Degrees of Freedom. ED=Extra-Dimensional. FDR=False Discovery Rate. HDGE=HD Gene Expanded. IED=Intra-Extra Dimensional Set Shift. IQ=Intelligence Quotient. ISCED=International Standard Classification of Education. NART=National Adult Reading Test. OTS=One Touch Stockings of Cambridge. PAL=Paired Associates Learning. RVP 'A=Rapid Visual Processing Accuracy. SDMT=Symbol Digit Modalities Test. SSRT=Stop Signal Reaction Time. SWM=Spatial Working Memory.

Supplementary Table 2: Longitudinal neuropsychiatric data

Outcomes	Control Mean	HDGE Mean	Estimate of Mean Difference	Df	Lower CL	Upper CL	*P* value	FDR
FrsBe Disinhibition	-0.837	0.215	1.052	125	0.026	2.077	0.045	0.379
SF-36 Physical Function	0.384	-0.831	-1.216	153	-2.543	0.111	0.072	0.379
SF-36 Energy Fatigue	-1.10	0.10	1.202	126	-0.134	2.538	0.077	0.379
FrsBe Executive Function	-1.319	-0.146	1.173	130	-0.161	2.508	0.084	0.379
SF-36 Limitations Physical Health	0.379	-0.735	-1.114	142	-2.592	0.364	0.139	0.499
STAI State	-0.444	-0.042	0.402	124	-0.254	1.059	0.227	0.682
SF-36 Pain	0.376	-0.323	-0.699	144	-2.090	0.692	0.322	0.747
SF-36 Limitations Emotional Problems	0.048	-1.181	-1.229	131	-3.727	1.269	0.332	0.747
AMI (Total)	-0.160	-0.196	-0.036	153	-0.148	0.075	0.521	0.947
PSQI (Total)	0.062	-0.004	-0.065	123	-0.269	0.138	0.526	0.947
FrsBe Apathy	-0.225	0.104	0.329	127	-0.883	1.541	0.592	0.960
SDS (Total)	0.157	0.029	-0.128	123	-0.680	0.425	0.648	0.960
STAI Trait	-0.211	-0.094	0.116	119	-0.466	0.699	0.693	0.960
SF-36 Social Functioning	0.421	0.185	-0.236	141	-1.869	1.397	0.776	0.961
SF-36 Emotional Wellbeing	0.072	-0.054	-0.125	120	-1.106	0.855	0.801	0.961
BIS-11 (Total)	-0.310	-0.365	-0.055	119	-0.724	0.614	0.871	0.979
SF-36 General Health	-0.165	-0.140	0.025	124	-1.203	1.254	0.968	0.999
OCI-R (Total)	-0.408	-0.408	0.000	118	-0.486	0.487	0.999	0.999

Table showing adjusted mean longitudinal changes in neuropsychiatric outcomes comparing controls to HDGE participants. Outcomes are arranged in ascending order of *P* value. Statistical analyses are based on mixed-effect linear models with a participant random effect. All models are controlled for age, sex, and age-by-sex interaction. Statistical two-sided group comparisons were adjusted for multiple comparisons using the FDR, with *P* values, degrees of freedom, and confidence limits provided in the table.

Abbreviations:
AMI=Apathy Motivation Index. BIS-11=Barratt Impulsiveness Scale. CL=Confidence Limit. Df=Degrees of Freedom. FDR=False Discovery Rate. FrsBe=Frontal Systems Behaviour Scale. HDGE=HD Gene Expanded. OCI-R=Obsessive Compulsive Inventory-Revised. PSQI=Pittsburgh Sleep Quality Index. SDS=Zung Self-Rating Depression Scale (SDS). SF-36=Short Form-36 Health Status. STAI=State-Trait Anxiety Inventory.

Supplementary Table 3: Cross-sectional cognitive data

Outcomes	Control Mean	HDGE Mean	Estimate of Mean Difference	Df	Lower CL	Upper CL	*P* value	FDR
**SSRT**	197	211	14.069	152	3.281	24.858	**0.011**	**0.131**
RVP 'A	0.93	0.92	-0.015	160	-0.028	-0.002	0.026	0.154
Verbal fluency (total correct)	25.9	24.1	-1.797	159	-3.498	-0.095	0.039	0.154
PAL (total error adjusted)	20.2	25.8	5.778	156	-2.372	13.928	0.163	0.410
SDMT	60.9	58.9	-2.006	159	-4.886	0.873	0.171	0.410
Goals Prior Assay (slope)	-0.68	-0.62	0.053	117	-0.033	0.139	0.221	0.442
RVP (mean latency)	451	462	11.679	158	-8.839	32.198	0.263	0.450
Intensity Face Morphing (negative)	7.71	8.04	0.328	143	-0.563	1.220	0.467	0.701
IED ED Shift Error	5.98	6.74	0.759	156	-1.432	2.950	0.495	0.835
IED Stage Completion	8.77	8.70	-0.072	148	-0.284	0.140	0.504	0.835
IED Pre ED Error	5.85	6.00	0.147	148	-0.449	0.743	0.626	0.835
IED ED Reversal Error	2.12	2.03	-0.090	145	-1.108	0.927	0.861	0.861
Agent Guilt Score	6.13	6.10	-0.029	156	-0.185	0.127	0.715	0.934
Stroop Interference	52.7	52.4	-0.304	119	-3.714	3.105	0.860	0.934
SWM (between errors)	69.9	70.8	0.861	159	-10.618	12.340	0.882	0.934
OTS (minimum moves)	11.9	11.8	-0.021	156	-0.533	0.490	0.934	0.934

Table showing adjusted mean cross-sectional cognitive outcomes comparing control participants to HDGE participants. Outcomes are arranged in ascending order of *P* value. Significant values at FDR <0.15 are highlighted in bold. Statistical analyses are based on mixed-effect linear models with a participant random effect. All models are controlled for age, sex, and age-by-sex interaction. These cognitive models also controlled for ISCED education level and estimated IQ via the NART score. Note the Goals Prior Assay and Stroop interference tasks were introduced at follow-up and tested by conventional linear regression rather than the mixed-effect linear models with a participant random effect used for longitudinal data. Statistical two-sided group comparisons were adjusted for multiple comparisons using the FDR, with *P* values, degrees of freedom, and confidence limits provided in the table.

Abbreviations:
CL=Confidence Limit. Df=Degrees of Freedom. ED=Extra-Dimensional. FDR=False Discovery Rate. HDGE=HD Gene Expanded. IED=Intra-Extra Dimensional Set Shift. IQ=Intelligence Quotient. ISCED=International Standard Classification of Education.

NART=National Adult Reading Test. OTS=One Touch Stockings of Cambridge. PAL=Paired Associates Learning. RVP 'A=Rapid Visual Processing Accuracy. SDMT=Symbol Digit Modalities Test. SSRT=Stop Signal Reaction Time. SWM=Spatial Working Memory.

Supplementary Table 4: Cross-sectional neuropsychiatric data

Outcomes	Control Mean	HDGE Mean	Estimate of Mean Difference	Df	Lower CL	Upper CL	*P* value	FDR
**SF-36 General Health**	74.3	67.2	-7.14	155	-12.1	-2.15	**0.005**	**0.096**
PSQI (total)	5.13	4.36	-0.77	153	-1.51	-0.03	0.042	0.381
OCI-R (total)	9.43	7.58	-1.85	156	-4.22	0.52	0.126	0.753
SF-36 Energy Fatigue	58.7	62.0	3.27	154	-2.00	8.53	0.222	0.798
SF-36 Pain	89.3	91.8	2.46	149	-1.18	6.10	0.183	0.798
BIS-11 (total)	59.9	60.4	0.48	156	-2.70	3.67	0.765	0.885
STAI Trait	39.6	39.1	-0.41	156	-3.22	2.41	0.777	0.885
SDS (total)	33.3	33.7	0.40	155	-1.95	2.75	0.737	0.885
AMI (total)	3.23	3.30	0.07	141	-0.21	0.35	0.627	0.885
FrsBe Apathy	27.6	27.0	-0.61	154	-5.08	3.85	0.786	0.885
FrsBe Disinhibition	28.4	26.9	-1.48	154	-5.43	2.48	0.463	0.885
SF-36 Physical Function	96.5	95.6	-0.88	141	-3.99	2.24	0.580	0.885
SF-36 Physical Limitations	95.2	96.3	1.12	150	-2.81	5.05	0.575	0.885
SF-36 Emotional Limitations	84.7	88.4	3.74	153	-4.60	12.1	0.377	0.885
SF-36 Emotional Wellbeing	75.8	76.5	0.73	156	-3.84	5.29	0.754	0.885
SF-36 Social Functioning	88.1	89.2	1.01	150	-3.38	5.39	0.651	0.885
FrsBe Executive Function	30.4	30.7	0.24	153	-4.33	4.81	0.917	0.971
STAI State	33.7	33.8	0.03	155	-2.58	2.63	0.984	0.984

Table showing adjusted mean cross-sectional neuropsychiatric outcomes comparing control participants to HDGE participants. Outcomes are arranged in ascending order of *P* value. Significant values at FDR <0.15 are highlighted in bold. Statistical analyses are based on mixed-effect linear models with a participant random effect. All models are controlled for age, sex, and age-by-sex interaction. Statistical two-sided group comparisons were adjusted for multiple comparisons using the FDR, with *P* values, degrees of freedom, and confidence limits provided in the table.

Abbreviations:
AMI=Apathy Motivation Index. BIS-11=Barratt Impulsiveness Scale. CL=Confidence Limit. Df=Degrees of Freedom. FDR=False Discovery Rate. FrsBe=Frontal Systems Behaviour Scale. HDGE=HD Gene Expanded. OCI-R=Obsessive Compulsive Inventory-

Revised. PSQI=Pittsburgh Sleep Quality Index. SDS=Zung Self-Rating Depression Scale (SDS). SF-36=Short Form-36 Health Status. STAI=State-Trait Anxiety Inventory.

Supplementary Table 5: Data included by modality

	Baseline		Follow-up		Longitudinal	
	HDGE	Controls	HDGE	Controls	HDGE	Controls
**Participants**	64	67	66	60	57	46
**T1 volumetric**	61	62	64	50	54	34
**DWI**	58	60	62	47	50	33
**MPM**	55	60	57	46	43	32
**Structural connectivity**	51	55	52	38	43	27
**Plasma**	58	51	62	45	53	33
**CSF**	58	51	62	45	53	33

Table summarising data included for each modality. Data were excluded either due to collection omissions or processing failures, as detailed in the text. Note that for structural connectivity, only data from right-handed participants were included.

Abbreviations:
CSF=Cerebrospinal Fluid. DWI=Diffusion Weighted Imaging. HDGE=HD Gene Expanded. MPM=Multiparametric Mapping.

Supplementary Table 6: Longitudinal structural connectivity data

Outcomes	Control Mean	HDGE Mean	Estimate of Mean Difference	Df	Lower CL	Upper CL	*P* value	FDR
Modularity	4.77×10^{-4}	3.31×10^{-3}	0.003	101	4.37×10^{-5}	5.62×10^{-3}	0.047	0.435
L Thalamus	-2.76×10^{-5}	-2.18×10^{-4}	0.000	88	-3.91×10^{-4}	9.61×10^{-6}	0.062	0.435
L Sensorimotor	4.99×10^{-5}	9.98×10^{-5}	0.000	102	-2.16×10^{-5}	3.21×10^{-4}	0.086	0.435
Efficiency	3.58×10^{-7}	3.45×10^{-7}	0.000	81	-1.51×10^{-6}	1.05×10^{-7}	0.087	0.435
R Sensorimotor	1.34×10^{-4}	-5.27×10^{-6}	0.000	107	-3.26×10^{-4}	4.81×10^{-5}	0.144	0.575
R Inferior Parietal	-1.34×10^{-5}	3.52×10^{-5}	0.000	76	-2.48×10^{-5}	1.22×10^{-4}	0.191	0.586
R Thalamus	-1.55×10^{-4}	-2.65×10^{-4}	0.000	84	-2.86×10^{-4}	6.70×10^{-5}	0.220	0.586
R Executive	6.91×10^{-5}	-5.06×10^{-5}	0.000	101	-3.25×10^{-4}	8.57×10^{-5}	0.250	0.586
R Rostral Middle Frontal	-1.13×10^{-4}	-3.48×10^{-5}	0.000	80	-6.01×10^{-5}	2.17×10^{-4}	0.264	0.586
R Superior Parietal	-2.66×10^{-5}	5.79×10^{-5}	0.000	82	-8.26×10^{-5}	2.52×10^{-4}	0.318	0.629
L Limbic	1.96×10^{-5}	-2.55×10^{-5}	0.000	104	-1.44×10^{-4}	5.35×10^{-5}	0.367	0.629
R Superior Frontal	-2.19×10^{-4}	-1.06×10^{-4}	0.000	86	-1.42×10^{-4}	3.68×10^{-4}	0.380	0.629
L Executive	8.63×10^{-5}	2.85×10^{-7}	0.000	98	-2.92×10^{-4}	1.20×10^{-4}	0.409	0.629
L Superior Frontal	-1.44×10^{-4}	-7.99×10^{-5}	0.000	81	-1.16×10^{-4}	2.44×10^{-4}	0.481	0.667
R Limbic	-1.71×10^{-5}	1.14×10^{-5}	0.000	96	-5.52×10^{-5}	1.12×10^{-4}	0.500	0.667
L Superior Parietal	4.34×10^{-5}	7.32×10^{-5}	0.000	83	-1.37×10^{-4}	1.97×10^{-4}	0.723	0.877
R Precentral	6.48×10^{-5}	3.37×10^{-5}	0.000	82	-2.22×10^{-4}	1.59×10^{-4}	0.746	0.877
L Inferior Parietal	4.25×10^{-5}	3.17×10^{-5}	0.000	79	-1.16×10^{-4}	9.43×10^{-5}	0.838	0.931
L Precentral	-8.87×10^{-5}	-8.64×10^{-5}	0.000	78	-9.06×10^{-5}	9.52×10^{-5}	0.961	0.963
L Rostral Middle Frontal	8.64×10^{-5}	8.38×10^{-5}	0.000	80	-1.15×10^{-4}	1.10×10^{-4}	0.963	0.963

Table showing adjusted mean longitudinal structural connectivity changes comparing control participants to HDGE participants. Outcomes are arranged in ascending order of *P* value. Statistical analyses are based on mixed-effect linear models with a participant random effect. All models are controlled for age, sex, and age-by-sex interaction. Statistical two-sided group comparisons were adjusted for multiple comparisons using the FDR, with *P* values, degrees of freedom, and confidence limits provided in the table.

Abbreviations:
CL=Confidence Limit. Df=Degrees of Freedom. FDR=False Discovery Rate. HDGE=HD Gene Expanded. L=Left. R=Right.

Supplementary Table 7: Longitudinal multiparametric mapping data

Outcomes	Control Mean	HDGE Mean	Estimate of Mean Difference	Df	Lower CL	Upper CL	*P* value	FDR
Putamen R2*	0.173	0.313	0.140	79	0.036	0.244	0.009	0.291
Mid Corpus Callosum MT	-8.29×10^{-4}	-5.62×10^{-3}	-4.80×10^{-3}	88	-0.009	-0.0004	0.033	0.420
Splenium Corpus Callosum R2*	0.039	-0.101	-0.140	93	-0.281	0.0004	0.051	0.420
Mid Corpus Callosum R2*	1.43×10^{-3}	-0.0677	-0.069	89	-0.139	0.001	0.053	0.420
Anterior Internal Capsule PD	-6.15×10^{-3}	-0.0711	-0.065	102	-0.147	0.017	0.120	0.731
Putamen PD	-0.038	-0.149	-0.111	97	-0.265	0.043	0.157	0.731
Anterior Internal Capsule R2*	0.070	-0.012	-0.082	96	-0.205	0.041	0.188	0.731
Genu Corpus Callosum R2*	-0.027	-0.110	-0.084	94	-0.211	0.044	0.196	0.731
External Capsule PD	-0.041	-0.109	-0.069	101	-0.182	0.045	0.234	0.731
Splenium Corpus Callosum R1	-1.57×10^{-3}	-1.34×10^{-4}	1.70×10^{-3}	100	-0.001	0.005	0.288	0.731
Genu Corpus Callosum MT	-3.46×10^{-3}	-6.89×10^{-3}	-3.40×10^{-3}	92	-0.010	0.003	0.295	0.731
Splenium Corpus Callosum MT	-5.85×10^{-4}	-4.33×10^{-3}	-3.70×10^{-3}	102	-0.012	0.004	0.369	0.731
Caudate MT	-1.25×10^{-3}	4.68×10^{-4}	1.70×10^{-3}	106	-0.002	0.006	0.372	0.731
External Capsule MT	1.58×10^{-4}	-1.37×10^{-3}	-1.50×10^{-3}	84	-0.005	0.002	0.375	0.731
Caudate PD	-0.066	-0.112	-0.046	95	-0.149	0.057	0.378	0.731
Anterior Internal Capsule MT	-3.22×10^{-3}	-4.57×10^{-4}	2.80×10^{-3}	97	-0.003	0.009	0.382	0.731
Mid Corpus Callosum PD	-2.90×10^{-3}	0.018	0.021	108	-0.028	0.069	0.397	0.731
Posterior Internal Capsule MT	-1.71×10^{-3}	-4.11×10^{-3}	-2.00×10^{-3}	94	-0.008	0.004	0.423	0.731
Putamen R1	1.86×10^{-3}	2.61×10^{-3}	7.00×10^{-4}	97	-0.001	0.003	0.434	0.731
Anterior Internal Capsule R1	5.80×10^{-4}	1.65×10^{-3}	1.10×10^{-3}	98	-0.002	0.004	0.513	0.800
Mid Corpus Callosum R1	8.30×10^{-4}	2.05×10^{-4}	-6.00×10^{-4}	89	-0.003	0.001	0.541	0.800
External Capsule R2*	0.028	8.11×10^{-3}	-0.020	90	-0.086	0.046	0.550	0.800
Splenium Corpus Callosum PD	9.33×10^{-3}	-5.33×10^{-3}	-0.015	108	-0.074	0.045	0.627	0.853
Posterior Internal Capsule PD	0.019	-5.42×10^{-4}	-0.020	105	-0.104	0.064	0.639	0.853
Caudate R1	1.51×10^{-3}	1.99×10^{-3}	1.00×10^{-3}	104	-0.002	0.003	0.702	0.882
Genu Corpus Callosum PD	0.026	0.014	-0.012	110	-0.083	0.059	0.736	0.882
Genu Corpus Callosum R1	-2.08×10^{-3}	-2.74×10^{-3}	-1.00×10^{-3}	101	-0.005	0.003	0.744	0.882
Caudate R2*	0.086	0.070	-0.016	86	-0.132	0.100	0.788	0.883
Putamen MT	-5.36×10^{-4}	-1.90×10^{-4}	3.00×10^{-4}	93	-0.002	0.003	0.800	0.883
External Capsule R1	7.50×10^{-4}	6.16×10^{-4}	-1.00×10^{-4}	92	-0.002	0.002	0.887	0.929

Posterior Internal Capsule R1	1.28×10^{-3}	1.49×10^{-3}	2.00×10^{-4}	102	-0.003	0.004	0.900	0.929
Posterior Internal Capsule R2*	2.34×10^{-3}	6.33×10^{-3}	4.00×10^{-3}	95	-0.109	0.117	0.944	0.944

Table showing adjusted mean longitudinal changes in multiparametric mapping outcomes comparing controls to HDGE participants. Outcomes are arranged in ascending order of *P* value. Statistical analyses are based on mixed-effect linear models with a participant random effect. All models are controlled for age, sex, and age-by-sex interaction. Statistical two-sided group comparisons were adjusted for multiple comparisons using the FDR, with *P* values, degrees of freedom, and confidence limits provided in the table.

Abbreviations:

CL=Confidence Limit. Df=Degrees of Freedom. FDR=False Discovery Rate. HDGE=HD Gene Expanded. MT=Magnetisation Transfer. PD=Proton Density. R1=Longitudinal Relaxation Rate. R2=Transverse Relaxation Rate.*

Supplementary Table 8: Cross-sectional volumetric data

Outcomes (% of ICV)	Control Mean	HDGE Mean	Estimate of Mean Difference	Df	Lower CL	Upper CL	*P* value	FDR
**Putamen**	0.65	0.60	-4.70×10^{-4}	153	-7.00×10^{-4}	-2.53×10^{-4}	**4.0×10^{-4}**	**0.002**
**Caudate**	0.49	0.49	-2.30×10^{-4}	152	-4.10×10^{-4}	-6.00×10^{-5}	**9.7×10^{-4}**	**0.029**
**Whole brain**	80.7	79.9	-8.35×10^{-3}	152	-0.016	-6.00×10^{-4}	**3.5×10^{-4}**	**0.070**
**White matter**	34.9	34.5	-4.15×10^{-3}	152	-9.00×10^{-3}	7.00×10^{-4}	**0.093**	**0.140**
**Grey matter**	43.6	43.2	-4.22×10^{-3}	153	-9.55×10^{-3}	1.11×10^{-3}	**0.120**	**0.144**
Ventricles	0.86	0.92	-6.60×10^{-4}	152	-6.50×10^{-4}	1.98×10^{-3}	0.032	0.319

Table showing adjusted mean cross-sectional volumetric outcomes comparing control participants to HDGE participants. Outcomes are arranged in ascending order of *P* value. Significant values at FDR <0.15 are highlighted in bold. Statistical analyses are based on mixed-effect linear models with a participant random effect. All models are controlled for age, sex, and age-by-sex interaction. Statistical two-sided group comparisons were adjusted for multiple comparisons using the FDR, with *P* values, degrees of freedom, and confidence limits provided in the table.

Abbreviations:

CL=Confidence Limit. Df=Degrees of Freedom. FDR=False Discovery Rate. HDGE=HD Gene Expanded.

Supplementary Table 9: Cross-sectional diffusion data

Outcomes	Control Mean	HDGE Mean	Estimate of Mean Difference	Df	Lower CL	Upper CL	*P* value	FDR
Posterior Internal Capsule ODI	0.133	0.127	-0.005	128	-0.009	-0.002	0.004	0.177
Genu Corpus Callosum AD	1.181	1.171	-0.010	128	-0.018	-0.002	0.011	0.234
Anterior Internal Capsule AD	0.976	0.983	0.007	128	0.0001	0.014	0.047	0.663
Genu Corpus Callosum FWF	0.087	0.084	-0.003	125	-0.007	0.0004	0.080	0.712
Anterior Internal Capsule FA	0.560	0.565	0.005	127	-0.001	0.011	0.104	0.712
Anterior Internal Capsule FWF	0.053	0.055	0.002	126	-0.0004	0.004	0.107	0.712
Genu Corpus Callosum MD	0.623	0.618	-0.005	128	-0.011	0.0013	0.121	0.712
Anterior Internal Capsule ODI	0.137	0.134	-0.002	128	-0.005	0.0007	0.136	0.712
External Capsule ODI	0.215	0.213	-0.002	128	-0.005	0.001	0.184	0.859
Posterior Internal Capsule FWF	0.074	0.076	0.002	121	-0.001	0.006	0.219	0.908
Posterior Internal Capsule NDI	0.669	0.673	0.004	128	-0.003	0.012	0.238	0.908
Mid Corpus Callosum NDI	0.628	0.622	-0.005	128	-0.015	0.004	0.290	0.917
Genu Corpus Callosum ODI	0.110	0.108	-0.001	125	-0.004	0.001	0.295	0.917
Posterior Internal Capsule FA	0.636	0.638	0.002	128	-0.003	0.007	0.364	0.917
Splenium Corpus Callosum FWF	0.179	0.174	-0.005	128	-0.015	0.006	0.382	0.917
Anterior Internal Capsule RD	0.360	0.358	-0.002	127	-0.007	0.003	0.417	0.917
Mid Corpus Callosum RD	0.365	0.368	0.003	128	-0.007	0.013	0.501	0.917
Mid Corpus Callosum FA	0.657	0.654	-0.002	128	-0.010	0.005	0.514	0.917
Posterior Internal Capsule RD	0.313	0.311	-0.001	128	-0.006	0.003	0.528	0.917
External Capsule FWF	0.022	0.023	0.0005	127	-0.001	0.002	0.542	0.917
Splenium Corpus Callosum AD	1.340	1.340	-0.004	127	-0.016	0.009	0.549	0.917
Posterior Internal Capsule AD	1.018	1.020	0.002	128	-0.005	0.010	0.554	0.917
Anterior Internal Capsule MD	0.565	0.566	0.001	128	-0.003	0.005	0.556	0.917
External Capsule FA	0.445	0.446	0.001	128	-0.003	0.005	0.558	0.917
Genu Corpus Callosum RD	0.344	0.342	-0.002	128	-0.009	0.005	0.561	0.917
Splenium Corpus Callosum NDI	0.662	0.659	-0.002	128	-0.011	0.007	0.629	0.917
Mid Corpus Callosum AD	1.280	1.280	-0.002	128	-0.012	0.008	0.633	0.917
External Capsule AD	0.942	0.944	0.001	128	-0.004	0.007	0.636	0.917
Splenium Corpus Callosum ODI	0.071	0.071	-0.0003	129	-0.002	0.001	0.641	0.917
Anterior Internal Capsule NDI	0.621	0.622	0.002	128	-0.005	0.008	0.661	0.917

Splenium Corpus Callosum FA	0.724	0.723	-0.0009	128	-0.006	0.004	0.732	0.917
Mid Corpus Callosum MD	0.669	0.671	0.002	128	-0.008	0.011	0.749	0.917
Splenium Corpus Callosum MD	0.661	0.659	-0.001	128	-0.010	0.008	0.779	0.917
Mid Corpus Callosum ODI	0.094	0.094	0.0004	127	-0.002	0.003	0.786	0.917
External Capsule RD	0.457	0.457	-0.0005	128	-0.005	0.004	0.802	0.917
Mid Corpus Callosum FWF	0.181	0.179	-0.001	128	-0.011	0.009	0.803	0.917
External Capsule NDI	0.515	0.516	0.0007	128	-0.005	0.006	0.807	0.917
Genu Corpus Callosum NDI	0.596	0.596	0.0004	128	-0.009	0.010	0.933	0.982
Posterior Internal Capsule MD	0.548	0.548	-0.0001	128	-0.004	0.004	0.960	0.982
Genu Corpus Callosum FA	0.636	0.636	0.0001	128	-0.006	0.006	0.964	0.982
External Capsule MD	0.619	0.619	0.0001	128	-0.004	0.004	0.974	0.982
Splenium Corpus Callosum RD	0.321	0.321	-0.0001	128	-0.0087	0.0085	0.982	0.982

Table showing adjusted mean cross-sectional diffusion outcomes comparing control participants to HDGE participants. Outcomes are arranged in ascending order of *P* value. Diffusion (AD, FA, MD, and RD) and NODDI (FWF, ODI, and NDI) metrics were derived for the following regions of interest: corpus callosum (genu, mid and splenium), internal capsule (anterior and posterior) and external capsule. Statistical analyses are based on mixed-effect linear models with a participant random effect. All models are controlled for age, sex, and age-by-sex interaction. Statistical two-sided group comparisons were adjusted for multiple comparisons using the FDR, with *P* values, degrees of freedom, and confidence limits provided in the table.

Abbreviations:
AD=Axial Diffusivity. Df=Degrees of Freedom. FA=Fractional Anisotropy. CL=Confidence Limit. FDR=False Discovery Rate. FWF=Free Water Fraction. HDGE=HD Gene Expanded. MD=Mean Diffusivity. NDI=Neurite Density Index. NODDI=Neurite Orientation Dispersion & Density Imaging. ODI=Orientation Dispersion Index. RD=Radial Diffusivity.

Supplementary Table 10: Cross-sectional structural connectivity data

Outcomes	Control Mean	HDGE Mean	Estimate of Mean Difference	Df	Lower CL	Upper CL	*P* value	FDR
L Superior Parietal	0.027	0.028	1.14×10^{-3}	127	2.20×10^{-4}	2.07×10^{-3}	0.016	0.312
R Executive	0.020	0.019	-5.46×10^{-4}	125	-1.21×10^{-3}	1.13×10^{-4}	0.104	0.690
R Rostral Middle Frontal	0.021	0.022	6.35×10^{-4}	127	-2.91×10^{-4}	1.56×10^{-3}	0.177	0.690
R Superior Frontal	0.041	0.041	8.27×10^{-4}	127	-3.79×10^{-4}	2.03×10^{-3}	0.177	0.690
L Rostral Middle Frontal	0.021	0.020	-4.83×10^{-4}	127	-1.24×10^{-3}	2.77×10^{-4}	0.211	0.690
L Thalamus	0.030	0.031	5.63×10^{-4}	127	-3.31×10^{-4}	1.46×10^{-3}	0.215	0.690
Modularity	0.441	0.435	5.34×10^{-3}	125	-1.43×10^{-2}	3.64×10^{-3}	0.242	0.690
L Precentral	0.032	0.032	4.40×10^{-4}	127	3.67×10^{-4}	1.25×10^{-3}	0.283	0.706
L Superior Frontal	0.042	0.043	5.46×10^{-4}	127	-5.55×10^{-4}	1.65×10^{-3}	0.328	0.730
R Precentral	0.031	0.030	4.54×10^{-4}	127	-1.54×10^{-3}	6.37×10^{-4}	0.412	0.794
L Inferior Parietal	0.020	0.020	3.13×10^{-4}	127	-4.83×10^{-4}	1.11×10^{-3}	0.438	0.794
R Thalamus	0.030	0.030	-3.38×10^{-4}	127	-1.28×10^{-3}	5.99×10^{-4}	0.476	0.794
L Limbic	0.008	0.008	-8.08×10^{-5}	125	-3.70×10^{-4}	2.09×10^{-4}	0.582	0.866
R Inferior Parietal	0.022	0.0215	2.02×10^{-4}	126	-1.04×10^{-3}	-6.34×10^{-4}	0.633	0.866
Efficiency	5.62×10^{-4}	5.63×10^{-4}	1.09×10^{-6}	127	4.06×10^{-5}	-6.24×10^{-6}	0.677	0.866
R Superior Parietal	0.028	0.0279	-1.58×10^{-4}	127	-1.17×10^{-3}	-8.50×10^{-4}	0.756	0.866
R Sensorimotor	0.011	0.0112	8.12×10^{-5}	124	-6.21×10^{-4}	-4.58×10^{-4}	0.766	0.866
L Executive	0.018	0.0182	9.90×10^{-5}	126	7.97×10^{-4}	-5.99×10^{-4}	0.780	0.866
L Sensorimotor	0.012	0.012	-1.70×10^{-5}	125	5.56×10^{-4}-	5.22×10^{-4}	0.950	0.962
R Limbic	8.24×10^{-3}	0.00825	7.02×10^{-6}	126	-2.84×10^{-4}	2.98×10^{-4}	0.962	0.962

Table showing adjusted mean cross-sectional structural connectivity outcomes comparing control participants to HDGE participants. Outcomes are arranged in ascending order of *P* value. Statistical analyses are based on mixed-effect linear models with a participant random effect. All models are controlled for age, sex, and age-by-sex interaction. Statistical two-sided group comparisons were adjusted for multiple comparisons using the FDR, with *P* values, degrees of freedom, and confidence limits provided in the table.

Abbreviations:
CL=Confidence Limit. Df=Degrees of Freedom. FDR=False Discovery Rate. HDGE=HD Gene Expanded. L=Left. R=Right.

Supplementary Table 11: Cross-sectional multiparametric mapping data

Outcomes	Control Mean	HDGE Mean	Estimate of Mean Difference	Df	Lower CL	Upper CL	*P* value	FDR
Putamen R1	0.766	0.777	0.011	125	0.004	0.019	0.003	0.102
Posterior Internal Capsule R2*	20.7	21.2	0.465	122	0.061	0.868	0.024	0.245
Caudate R1	0.719	0.728	0.465	123	0.001	0.017	0.025	0.245
Putamen R2s	22.0	22.9	0.465	123	0.086	1.738	0.031	0.245
External Capsule R1	0.902	0.910	0.008	126	-0.001	0.017	0.065	0.416
External Capsule R2*	18.7	19.0	0.248	122	-0.028	0.525	0.078	0.417
Anterior Internal Capsule R1	1.02	1.03	0.010	125	-0.003	0.022	0.128	0.584
Putamen MT	0.912	0.905	-0.007	124	-0.017	0.004	0.202	0.686
Anterior Internal Capsule PD	69.38	69.21	-0.177	123	-0.454	0.100	0.208	0.686
Anterior Internal Capsule R2*	22.9	22.6	-0.268	121	-0.703	0.168	0.226	0.686
Caudate R2*	18.9	19.2	0.334	123	-0.221	0.889	0.236	0.686
Genu Corpus Callosum MT	1.70	1.69	-0.015	124	-0.040	0.011	0.263	0.701
Splenium Corpus Callosum R2*	24.0	23.7	-0.267	122	-0.804	0.271	0.328	0.807
Mid Corpus Callosum R1	1.03	1.03	0.004	126	-0.007	0.015	0.458	0.867
Anterior Internal Capsule MT	1.53	1.52	-0.008	123	-0.029	0.014	0.470	0.867
Posterior Internal Capsule MT	1.54	1.53	-0.008	123	-0.029	0.014	0.482	0.867
External Capsule PD	71.18	71.04	-0.144	123	-0.548	0.261	0.484	0.867
Mid Corpus Callosum MT	1.58	1.57	-0.007	125	-0.027	0.013	0.487	0.867
Posterior Internal Capsule PD	67.47	67.39	-0.080	122	-0.342	0.183	0.549	0.890
Caudate MT	0.840	0.837	-0.003	120	-0.014	0.008	0.573	0.890
Genu Corpus Callosum R1	1.08	1.09	0.004	124	-0.010	0.018	0.593	0.890
Genu Corpus Callosum PD	67.58	67.64	0.051	121	-0.149	0.252	0.612	0.890
Putamen PD	78.4	78.3	-0.125	124	-0.723	0.472	0.678	0.916
Posterior Internal Capsule R1	1.01	1.01	0.002	124	-0.009	0.014	0.720	0.916
Mid Corpus Callosum PD	68.14	68.17	0.025	122	-0.119	0.169	0.729	0.916
Splenium Corpus Callosum R1	1.04	1.04	0.002	124	-0.010	0.013	0.760	0.916
Splenium Corpus Callosum PD	68.08	68.05	-0.026	121	-0.207	0.154	0.773	0.916
External Capsule MT	1.39	1.39	0.002	125	-0.016	0.020	0.827	0.930
Splenium Corpus Callosum MT	1.61	1.61	-0.003	121	-0.028	0.023	0.843	0.930

Mid Corpus Callosum R2*	21.1	21.1	0.018	122	-0.283	0.319	0.906	0.967
Genu Corpus Callosum R2*	22.9	22.8	-0.010	122	-0.483	0.463	0.966	0.997
Caudate PD	80.12	80.12	0.001	125	-0.433	0.434	0.998	0.998

Table showing adjusted mean cross-sectional multiparametric mapping outcomes comparing controls to HDGE participants. Outcomes are arranged in ascending order of *P* value. Statistical analyses are based on mixed-effect linear models with a participant random effect. All models are controlled for age, sex, and age-by-sex interaction. Statistical two-sided group comparisons were adjusted for multiple comparisons using the FDR, with *P* values, degrees of freedom, and confidence limits provided in the table.

Abbreviations:
CL=Confidence Limit. Df=Degrees of Freedom. FDR=False Discovery Rate. HDGE=HD Gene Expanded. MT=Magnetisation Transfer. PD=Proton Density. R1=Longitudinal Relaxation Rate. R2=Transverse Relaxation Rate.*

Supplementary Table 12: Log CSF NfL cross-sectional model

	**Regression Coefficient**	**SE**	**Df**	**t value**	***P* value**
(Intercept)	5.136	0.103	141	49.745	$2.99{\times}10^{-91}$
Age - 30	0.059	0.012	179	5.102	$8.50{\times}10^{-7}$
Sex	0.187	0.063	141	2.967	$3.53{\times}10^{-3}$
HDGE(a)	-0.032	0.095	166	-0.329	$7.43{\times}10^{-1}$
(Age - 30) * Sex	-0.023	0.007	184	-3.218	$1.52{\times}10^{-3}$
HDGE * (CAG - 40)	0.347	0.031	161	11.100	$1.31{\times}10^{-21}$
(Age - 30) * HDGE	0.045	0.010	176	4.457	$1.48{\times}10^{-5}$
(Age - 30) * (CAG - 40) * HDGE	0.013	0.003	166	3.991	$9.86{\times}10^{-5}$

a HDGE =1 if HDGE present; =0 for controls

Joint test of the age and CAG terms interaction within HDGE group: χ^2=139.3, 3 Df, $P<10^{-15}$

Table showing the cross-sectional regression model for log-transformed CSF NfL. Additionally, cross-sectionally, log concentrations of CSF NfL (χ^2=139.3, 3 Df, $P<10^{-15}$) were highly associated jointly with age, CAG length, and their interaction within the HDGE group. As indicated in the above regression coefficient table, age and CAG effects specific to the HDGE group controlled for age and sex effects in the controls and for the average log concentrations in the HDGE group. To make the main effects of age and CAG more interpretable relative to values observed in the study, we center age at 30 and CAG length at 40. After adjustment for age and sex effects in the controls and for the average log concentrations in the HDGE group.

Abbreviations:

CSF=Cerebrospinal Fluid. Df=Degrees of Freedom. HDGE=HD Gene Expanded. NfL=Neurofilament light. SE=Standard Error.

Supplementary Table 13: Log CSF PENK cross-sectional model

	Regression Coefficient	SE	Df	t value	*P* value
(Intercept)	1.3897	0.0497	136	27.945	2.52×10^{-58}
Age - 30	-0.0133	0.0052	161	-2.547	1.18×10^{-2}
Sex	-0.0976	0.0303	137	-3.222	1.59×10^{-3}
HDGE(a)	0.0247	0.0457	161	0.540	5.90×10^{-1}
(Age - 30) * Sex	0.0030	0.0032	166	0.932	3.53×10^{-1}
HDGE * (CAG - 40)	-0.0704	0.0150	156	-4.704	5.59×10^{-6}
(Age - 30) * HDGE	-0.0077	0.0046	156	-1.688	9.34×10^{-2}
(Age - 30) * (CAG - 40) * HDGE	-0.0031	0.0015	144	-2.026	4.46×10^{-2}

a HDGE =1 if HDGE present; =0 for controls

Joint test of the age and CAG terms interaction within HDGE group: χ^2=34.3, 3 Df, *P*=1.72×10^{-7}

Table showing the cross-sectional regression model for log-transformed CSF PENK. Additionally, cross-sectionally, log concentrations of CSF PENK (χ^2=34.3, 3 Df, *P*=1.72×10^{-7}) were highly associated jointly with age, CAG length, and their interaction within the HDGE group. As indicated in the above regression coefficient table, age and CAG effects specific to the HDGE group controlled for age and sex effects in the controls and for the average log concentrations in the HDGE group. To make the main effects of age and CAG more interpretable relative to values observed in the study, we center age at 30 and CAG length at 40. After adjustment for age and sex effects in the controls and for the average log concentrations in the HDGE group.

Abbreviations:

CSF=Cerebrospinal Fluid. Df=Degrees of Freedom. HDGE=HD Gene Expanded. PENK=Proenkephalin. SE=Standard Error.

Supplementary Table 14: Log plasma NfL cross-sectional model

	**Regression Coefficient**	**SE**	**Df**	**t value**	***P* value**
(Intercept)	1.8968	0.115	145	16.432	6.91×10^{-35}
Age - 30	0.0366	0.017	205	2.220	2.75×10^{-2}
Sex	-0.0059	0.070	146	-0.083	9.34×10^{-1}
HDGE(a)	-0.1206	0.113	166	-1.065	2.89×10^{-1}
(Age - 30) * Sex	-0.0119	0.010	204	-1.186	2.37×10^{-1}
HDGE * (CAG - 40)	0.1859	0.036	159	5.146	7.76×10^{-7}
(Age - 30) * HDGE	0.0329	0.015	207	2.238	2.63×10^{-2}
(Age - 30) * (CAG - 40) * HDGE	0.0048	0.005	209	0.977	3.30×10^{-1}

a HDGE =1 if HDGE present; =0 for controls

Joint test of the age and CAG terms interaction within HDGE group: χ^2=31.33, 3 Df, *P*=7.24×10^{-7}

Table showing the cross-sectional regression model for log-transformed plasma NfL. Additionally, cross-sectionally, log concentrations of plasma NfL (χ^2=31.33, 3 Df, *P*=7.24×10^{-7}) were highly associated jointly with age, CAG length, and their interaction within the HDGE group. As indicated in the above regression coefficient table, age and CAG effects specific to the HDGE group controlled for age and sex effects in the controls and for the average log concentrations in the HDGE group. To make the main effects of age and CAG more interpretable relative to values observed in the study, we center age at 30 and CAG length at 40. After adjustment for age and sex effects in the controls and for the average log concentrations in the HDGE group.

Abbreviations:

CSF=Cerebrospinal Fluid. Df=Degrees of Freedom. HDGE=HD Gene Expanded. NfL=Neurofilament light. SE=Standard Error.

Supplementary Table 15: Cross-sectional HD-ISS progression comparisons for CSF NfL

Controlling for age and sex (and age-by-sex interaction)						
**Group**	**emmean (pg/ml, log scale)**	**SE**	**Df**	**Lower CL**	**Upper CL**	***P* value**
HD-ISS 0 → 0 – stable	6.11	0.097	44	5.92	6.31	$7.67×10^{-45}$
HD-ISS 0 → 1	6.89	0.165	43	6.55	7.22	$7.44×10^{-36}$
HD-ISS 1 → 1 – stable	6.37	0.125	42	6.12	6.62	$2.21×10^{-39}$

Results are averaged over the levels of: sex; Df method: Kenward-Roger; CL used: 0.95.

**Contrast**	**Mean Difference**	**SE**	**Df**	**t-ratio**	***P* value**
HD-ISS 0 progressors vs stable	-0.776	0.187	43	-4.146	0.0004
HD-ISS 1 stable vs HD-ISS 0 stable	-0.261	0.161	43	-1.623	0.2469
HD-ISS 0 progressors vs HD-ISS 1 stable	0.515	0.209	43	2.464	0.0461*

** Significant if controlling for age and sex but non-significant if these are not controlled (<u>see below</u>).*

**<u>Not</u> controlling for age and sex**						
**Group**	**emmean (pg/ml, log scale)**	**SE**	**Df**	**Lower CL**	**Upper CL**	***P* value**
HD-ISS 0 → 0 – stable	6.08	0.108	48	5.86	6.30	$8.67×10^{-45}$
HD-ISS 0 → 1	6.77	0.181	46	6.41	7.13	$4.38×10^{-36}$
HD-ISS 1 → 1 – stable	6.39	0.140	45	6.11	6.67	$2.48×10^{-39}$

Df method: Kenward-Roger; CL used: 0.95.

**Contrast**	**Mean Difference**	**SE**	**Df**	**t-ratio**	***P* value**
HD-ISS 0 progressors vs stable	-0.690	0.211	47	-3.276	0.0056
HD-ISS 1 stable vs HD-ISS 0 stable	-0.306	0.176	45	-1.736	0.2028
HD-ISS 0 progressors vs HD-ISS 1 stable	0.384	0.220	46	1.680	0.2237

Table showing HD-ISS progression comparisons for CSF NfL based on repeated cross-sectional measures over the two visits. Estimated marginal means (emmeans) of log-transformed CSF NfL are shown for each grouping, with comparisons both controlled and not controlled for age, sex, and age-by-sex interaction. The Kenward-Roger method was used for degrees of freedom and a 95% confidence level was applied. Here, HD-ISS 0 → 0 indicates stable HD-ISS 0 participants, HD-ISS 0 → 1 represents progressors from HD-ISS 0 to HD-ISS 1, and HD-ISS 1 → 1 denotes stable HD-ISS 1 participants.

Abbreviations:

CL=Confidence Limit. CSF=Cerebrospinal Fluid. Df=Degrees of Freedom. HDGE=HD Gene Expanded. HD-ISS=Huntington's Disease Integrated Staging System. NfL=Neurofilament light. SE=Standard Error.

Supplementary Table 16: Cross-sectional HD-ISS progression comparisons for plasma NfL

**Controlling for age and sex (and age-by-sex interaction)**						
**Group**	**emmean (pg/ml, log scale)**	**SE**	**Df**	**Lower CL**	**Upper CL**	***P* value**
HD-ISS 0 → 0 – stable	2.15	0.081	44	1.99	2.32	1.27×10^{-28}
HD-ISS 0 → 1	2.47	0.140	43	2.18	2.75	2.29×10^{-21}
HD-ISS 1 → 1 – stable	2.45	0.106	43	2.26	2.69	4.35×10^{-26}

Results are averaged over the levels of: sex; Df method: Kenward-Roger; CL used: 0.95.

**Contrast**	**Mean Difference**	**SE**	**Df**	**t-ratio**	***P* value**
HD-ISS 0 progressors vs stable	-0.312	0.158	44	-1.976	0.130
HD-ISS 1 stable vs HD-ISS 0 stable	0.323	0.136	44	-2.379	0.056
HD-ISS 0 progressors vs HD-ISS 1 stable	-0.011	0.177	43	-0.062	0.998

**Not controlling for age and sex**						
**Group**	**emmean (pg/ml, log scale)**	**SE**	**Df**	**Lower CL**	**Upper CL**	***P* value**
HD-ISS 0 → 0 – stable	2.17	0.085	47	2.00	2.34	3.57×10^{-29}
HD-ISS 0 → 1	2.46	0.144	47	2.17	2.75	1.56×10^{-21}
HD-ISS 1 → 1 – stable	2.45	0.111	46	2.23	2.68	4.25×10^{-26}

Df method: Kenward-Roger; CL used: 0.95.

**Contrast**	**Mean Difference**	**SE**	**Df**	**t-ratio**	***P* value**
HD-ISS 0 progressors vs stable	-0.294	0.167	47	-1.758	0.195
HD-ISS 1 stable vs HD-ISS 0 stable	-0.284	0.140	47	-2.029	0.117
HD-ISS 0 progressors vs HD-ISS 1 stable	-0.010	0.182	46	-0.055	0.998

Table showing HD-ISS progression comparisons for plasma NfL based on repeated cross-sectional measures over the two visits. Estimated marginal means (emmeans) of log-transformed plasma NfL are shown for each grouping, with comparisons both controlled and not controlled for age, sex, and age-by-sex interaction. The Kenward-Roger method was used for degrees of freedom and a 95% confidence level was applied. Here, HD-ISS 0 → 0 indicates stable HD-ISS 0 participants, HD-ISS 0 → 1 represents progressors from HD-ISS 0 to HD-ISS 1, and HD-ISS 1 → 1 denotes stable HD-ISS 1 participants.

Abbreviations:
CL=Confidence Limit. Df=Degrees of Freedom. HDGE=HD Gene Expanded. HD-ISS=Huntington’s Disease Integrated Staging System. NfL=Neurofilament light. SE=Standard Error.

Supplementary Table 17: Longitudinal biofluid data

Fluid biomarker [log transformed]	Control Mean	HDGE Mean	Estimate of Mean Difference	Lower CL	Upper CL	*P* value	FDR
**CSF NfL**	0.019	0.103	0.084	0.064	0.103	**3.2×10^{-13}**	**3.2×10^{-12}**
**CSF PENK**	-0.008	-0.024	-0.016	-0.025	-0.007	**4.14×10^{-4}**	**2.6×10^{-3}**
**CSF YKL-40**	0.010	0.031	0.021	0.004	0.038	**0.014**	**0.056**
CSF Tau	0.024	0.039	0.015	-0.002	0.030	0.075	0.230
Plasma Tau	0.037	0.001	-0.036	-0.093	0.021	0.217	0.570
Plasma NfL	0.045	0.064	0.019	-0.020	0.058	0.336	0.669
Plasma UCH-L1	-0.052	-0.093	-0.041	-0.135	0.053	0.387	0.669
CSF IL-6	0.047	0.061	0.013	-0.032	0.058	0.559	0.848
CSF GFAP	0.005	0.0028	-0.003	-0.022	0.017	0.799	0.917
CSF UCH-L1	0.036	0.032	-0.004	-0.039	0.031	0.831	0.917
CSF IL-8	-0.008	-0.010	-0.002	-0.027	0.023	0.855	0.917
Plasma GFAP	-0.005	-0.006	-0.001	-0.029	0.027	0.922	0.917

Table showing estimated mean change in longitudinal biofluid measures comparing control participants to HDGE participants. All statistical analyses were conducted using mixed-effect linear models with a participant-specific random effect, controlling for age, sex, and their interaction. Natural log-transformed concentrations served as the outcomes in these models. Statistical two-sided group comparisons were adjusted for multiple comparisons using the FDR, with *P* values and confidence limits provided in the table. Significant values at FDR <0.15 are highlighted in bold. The SIMOA Neurology 4-Plex A (GFAP, NfL, Tau, UCH-L1) was measured in singlicate, yielding the following inter-plate coefficients of variation (%CV): CSF GFAP (3.7%), CSF NfL (8.2%), CSF Tau (11.1%), CSF UCH-L1 (62.8%), plasma GFAP (5.6%), plasma NfL (5.0%), plasma Tau (11.1%), and plasma UCH-L1 (63.8%). CSF IL-6, IL-8, and YKL-40 were measured in duplicate, with the following %CVs: CSF YKL-40 (10.4%), CSF IL-6 (29.2%), and CSF IL-8 (12.2%). Relative CSF PENK levels were quantified in single measurements using an unbiased liquid chromatography-mass spectrometry-based proteomics approach with tandem mass tag labelling.

Abbreviations:

CL=Confidence Limit. CSF=Cerebrospinal Fluid. FDR=False Discovery Rate. GFAP=Glial Fibrillary Acidic Protein. HDGE=HD Gene Expanded. IL=Interleukin. NfL=Neurofilament Light. PENK=Proenkephalin. SIMOA=Single Molecule Array. UCH-L1=Ubiquitin Carboxyl-terminal Hydrolase L1. YKL-40, also known as Chitinase-3 like-protein-1 (CHI3L1).

Supplementary Table 18: Cross-sectional biofluid data

Fluid biomarker [log transformed]	Control Mean	HDGE Mean	Estimate of Mean Difference	Lower CL	Upper CL	*P* value	FDR
**CSF NfL**	5.48	6.23	0.750	0.587	0.913	**7.5×10^{-16}**	**8.2×10^{-15}**
**CSF PENK**	1.22	1.09	-0.130	-0.192	-0.067	**7.5×10^{-5}**	**3.0×10^{-4}**
**Plasma NfL**	1.93	2.25	0.324	0.173	0.476	**4.1×10^{-5}**	**2.2×10^{-4}**
**CSF YKL-40**	11.2	11.3	0.174	0.079	0.269	**4.3×10^{-4}**	**1.6×10^{-3}**
CSF IL-8	3.44	3.48	0.046	-0.035	0.128	0.263	0.615
Plasma GFAP	4.36	4.43	0.073	-0.060	0.207	0.280	0.615
Plasma Tau	2.10	2.03	-0.070	-0.221	0.082	0.364	0.668
CSF Tau	4.65	4.64	-0.004	-0.101	0.093	0.935	0.935
CSF GFAP	8.88	8.88	-0.008	-0.119	0.103	0.882	0.935
CSF UCH-L1	7.48	7.50	0.016	-0.066	0.098	0.701	0.935
CSF IL-6	0.47	0.49	0.023	-0.103	0.149	0.716	0.935
Plasma UCH-L1	3.99	3.98	-0.012	-0.230	0.206	0.913	0.935

Table showing estimated mean cross-sectional biofluid measures comparing control participants to HDGE participants. All statistical analyses were conducted using mixed-effect linear models with a participant-specific random effect, controlling for age, sex, and their interaction. Natural log-transformed concentrations served as the outcomes in these models. Statistical two-sided group comparisons were adjusted for multiple comparisons using the FDR, with *P* values and confidence limits provided in the table. Significant values at FDR <0.15 are highlighted in bold. The SIMOA Neurology 4-Plex A (GFAP, NfL, Tau, UCH-L1) was measured in singlicate, yielding the following inter-plate coefficients of variation (%CV): CSF GFAP (3.7%), CSF NfL (8.2%), CSF Tau (11.1%), CSF UCH-L1 (62.8%), plasma GFAP (5.6%), plasma NfL (5.0%), plasma Tau (11.1%), and plasma UCH-L1 (63.8%). CSF IL-6, IL-8, and YKL-40 were measured in duplicate, with the following %CVs: CSF YKL-40 (10.4%), CSF IL-6 (29.2%), and CSF IL-8 (12.2%). Relative CSF PENK levels were quantified in single measurements using an unbiased liquid chromatography-mass spectrometry-based proteomics approach with tandem mass tag labelling.

Abbreviations:

CL=Confidence Limit. CSF=Cerebrospinal Fluid. FDR=False Discovery Rate. GFAP=Glial Fibrillary Acidic Protein. HDGE=HD Gene Expanded. IL=Interleukin. NfL=Neurofilament Light. PENK=Proenkephalin. SIMOA=Single Molecule Array. UCH-L1=Ubiquitin Carboxyl-terminal Hydrolase L1. YKL-40, also known as Chitinase-3 like-protein-1 (CHI3L1).

Supplementary Table 19: Biofluid assay details

Analyte	Source	Assay	Platform	Manufacturer	Performed by
mHTT	CSF	2B7-MW1	SMCxPRO	MilliporeSigma	IRBM
NfL	CSF	Neurology 4-Plex A	SIMOA HD-X	Quanterix	UCL
NfL	Plasma	Neurology 4-Plex A	SIMOA HD-X	Quanterix	UCL
Tau	CSF	Neurology 4-Plex A	SIMOA HD-X	Quanterix	UCL
Tau	Plasma	Neurology 4-Plex A	SIMOA HD-X	Quanterix	UCL
GFAP	CSF	Neurology 4-Plex A	SIMOA HD-X	Quanterix	UCL
GFAP	Plasma	Neurology 4-Plex A	SIMOA HD-X	Quanterix	UCL
UCH-L1	CSF	Neurology 4-Plex A	SIMOA HD-X	Quanterix	UCL
UCH-L1	Plasma	Neurology 4-Plex A	SIMOA HD-X	Quanterix	UCL
YKL-40	CSF	Human YKL-40	U-PLEX	MSD	UCL
IL-6	CSF	Human IL-6	V-PLEX	MSD	UCL
IL-8	CSF	Human IL-8	V-PLEX	MSD	UCL
PENK	CSF	Unbiased TMT-based LC-MS proteomics	Orbitrap Lumos Tribrid with FAIMS Pro	Thermo Fisher Scientific	Gothenburg

Table providing details on the assays used to measure various biofluid analytes in CSF and plasma. For each analyte, the table specifies the source, assay type, platform, manufacturer, and institution responsible for performing the assay.

Abbreviations:
CSF=Cerebrospinal Fluid. FAIMS=Field Asymmetric Ion Mobility Spectrometry. GFAP=Glial Fibrillary Acidic Protein. IL=Interleukin. LC-MS=Liquid Chromatography-Mass Spectrometry. mHTT=Mutant Huntingtin. MSD=Meso Scale Discovery. NfL=Neurofilament Light. PENK=Proenkephalin. SIMOA=Single Molecule Array. SMC=Single Molecule Counting. TMT=Tandem Mass Tag. UCH-L1=Ubiquitin Carboxy-terminal Hydrolase L1. YKL-40, also known as Chitinase-3 like-protein-1 (CHI3L1).

Supplementary Figure 1: Cross-sectional differences in (a) cognitive and (b) neuropsychiatric measures between the HDGE and control groups

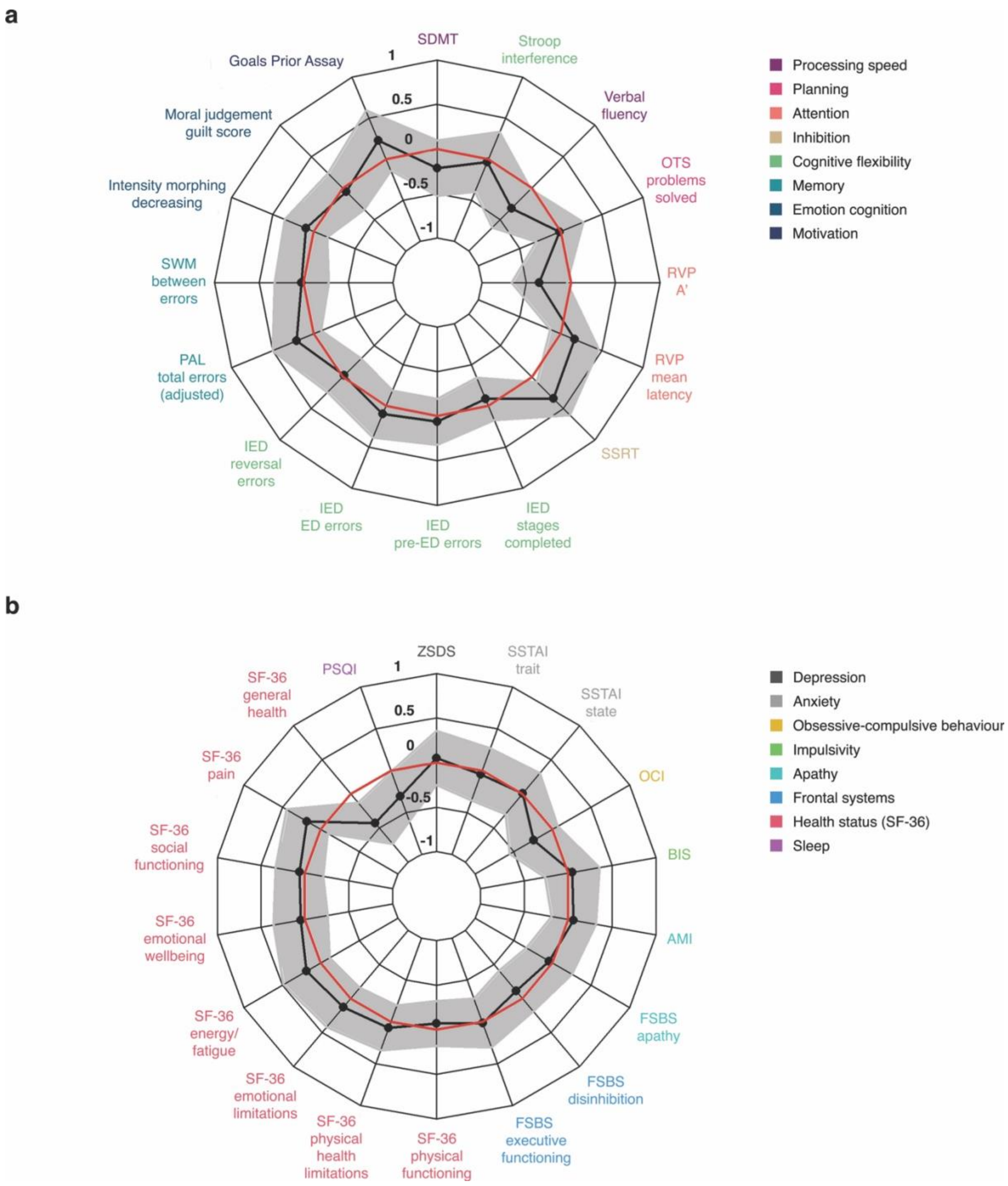

Radar plot showing cross-sectional differences between HDGE and control participants in (a) cognitive and (b) neuropsychiatric measures. The black line shows the standardised mean difference between the HDGE and control groups, with conventional frequentist 95% CI shaded in grey. The red circle represents no difference between means (i.e., the null hypothesis) and values within this circle represent lower scores in the HDGE group. After FDR correction for multiple comparisons, there were poorer performances in the HDGE group compared to

controls in the SST, RVP and verbal fluency task. The HDGE group reported poorer general health on the SF-36 compared to controls.

Abbreviations:
AMI=Apathy Motivation Index. BIS=Barratt Impulsivity Scale. CI=Confidence Interval. ED=Extra Dimensional. FDR=False Discovery Rate. FSBS=Frontal Systems Behavioural Scale. HDGE=HD Gene Expanded. HD-ISS=Huntington's Disease Integrated Staging System. IED=Intra-Extra Dimensional Set Shifting. OCI=Obsessive Compulsive Inventory. OTS=One Touch Stockings. PAL=Paired Associates Learning. RT=Reaction Time. RVP=Rapid Visual Processing. RVP A'=a signal detection theory measure of target sensitivity and mean response latency. SDMT=Symbol Digit Modalities Test. SF36=36-item self-report survey. SST=Stop Signal Task. SWM=Spatial Working Memory. SSTAI=Speilberger State Trait Anxiety Inventory. PSQI=Pittsburgh Sleep Quality Index. ZSDS=Zung Self-rating Depression Score.

Supplementary Figure 2: CSF mHTT assay performance

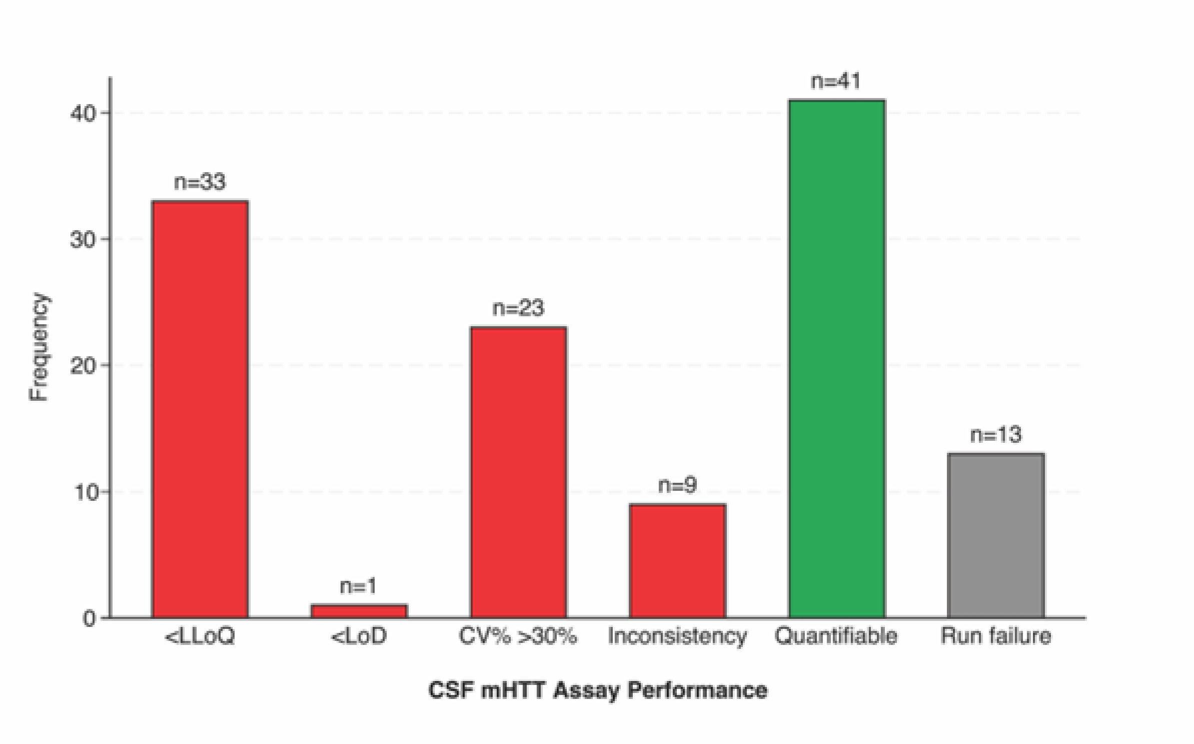

Bar chart showing the frequency of outcomes from the CSF mHTT assay performance in 120 CSF samples from the HDGE group. The outcomes are categorised as follows: 33 samples were below the lower limit of quantification (<LLoQ), 1 sample was below the limit of detection (<LoD), 23 samples had an unacceptable coefficient of variation greater than 30% (CV% >30%), 9 samples showed inconsistency, 41 samples were quantifiable (green bar), and 13 samples resulted in run failure (grey bar). Each bar represents the number of samples (n) in each category.

Abbreviations:

CSF=Cerebrospinal Fluid. CV%=Coefficient of Variation. <LLoQ=Below Lower Limit of Quantification. <LoD=Below Limit of Detection. mHTT=Mutant Huntingtin.

Supplementary Figure 3: Composite image of the CANTAB tests

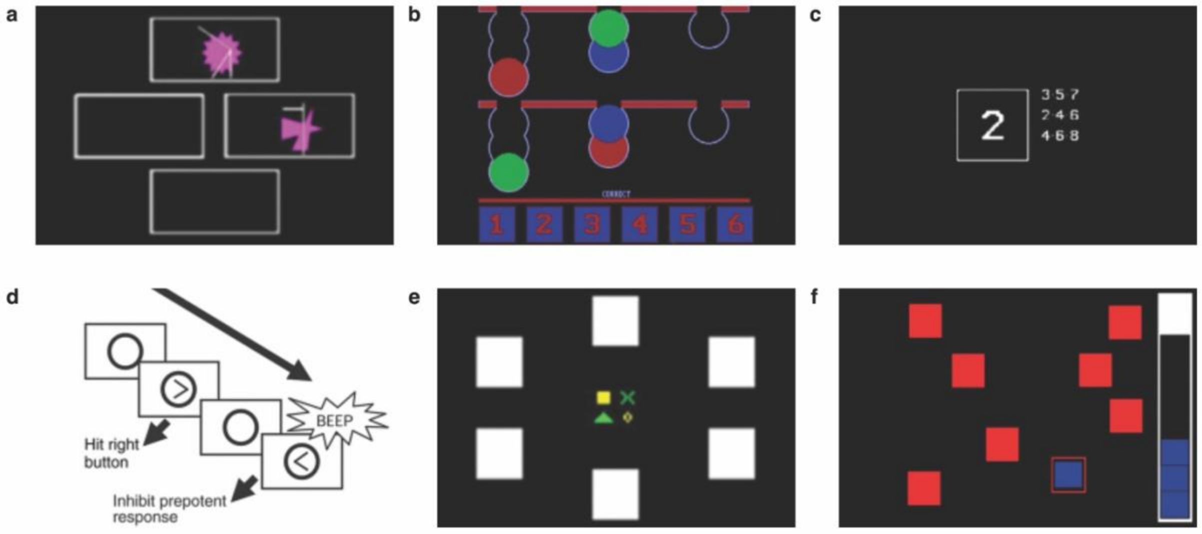

Panel illustrating CANTAB tests: (a) Intra-Extra Dimensional Set Shifting; (b) One Touch Stockings of Cambridge; (c) Rapid Visual Informational Visual Processing; (d) Stop Signal Test; (e) Paired Associate Leaning; and (f) Spatial Working Memory. For further details on each test, please refer to the **Supplementary Methods.**

Supplementary Figure 4: EMOTICOM Moral Judgment test

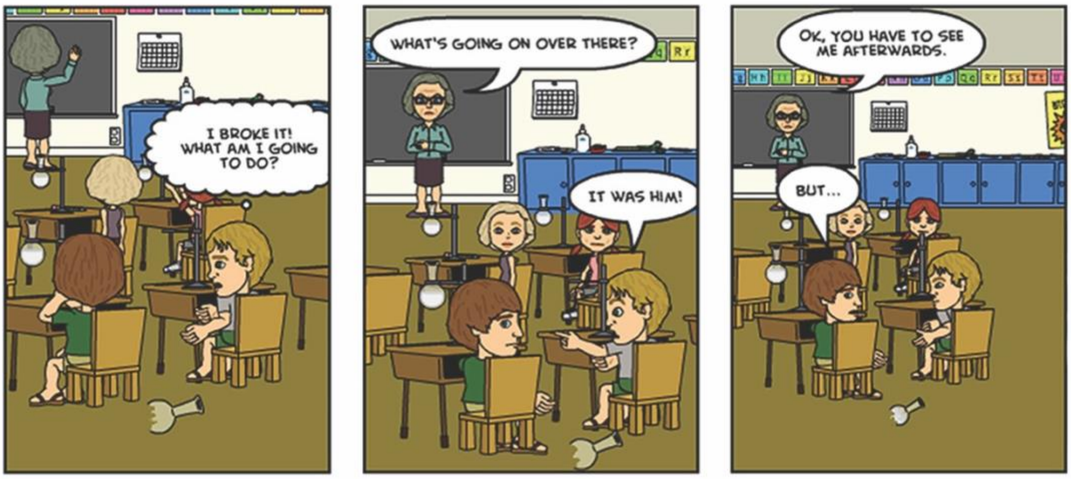

Panel illustrating EMOTICOM Moral Judgment test. This figure was created from the task itself, which is the task devised by Bland et al. (2016).[6] The 20-minute moral emotions test presents participants with cartoon depictions of moral scenarios. After each scenario, participants are asked to rate their levels of guilt, shame, annoyance, and feeling “bad”. Half of the cartoons are portrayed as deliberate harm and half as unintended harm. Participants were asked to rate their emotions from the perspective of both the victim and the perpetrator. The main outcome measure defined for this study was the guilt score collapsed across all conditions (deliberate vs. unintentional and perpetrator vs. victim).

Supplementary Figure 5: Goals Prior Assay task

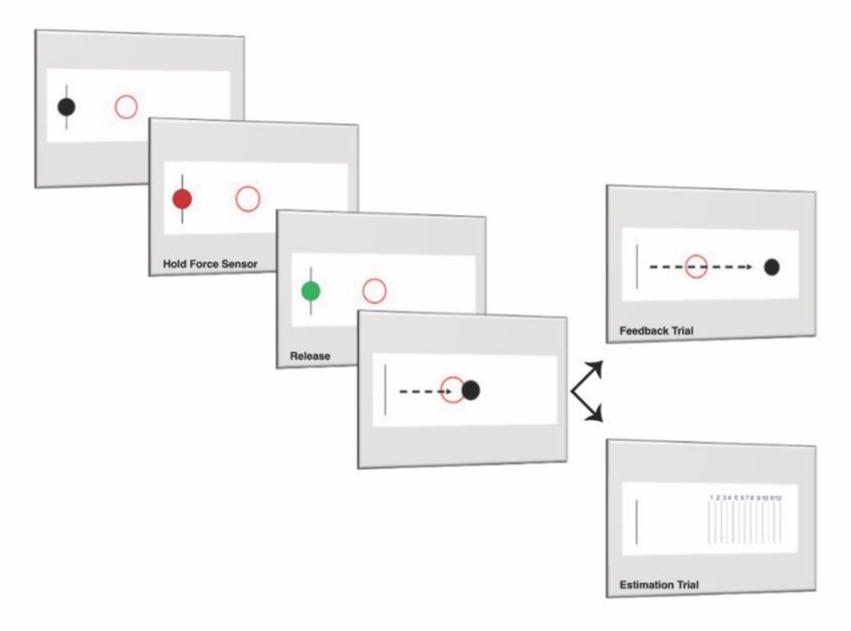

Panel showing pipeline of the 'Goals Prior Assay' task, which originated as a behavioural paradigm of apathy under a Bayesian conceptual framework[9]. When the ball appears, participants are asked to press a force sensor, at which point the ball will turn red. After 3 seconds the ball turns green, and participants are instructed to release the force sensor. At this point the virtual ball travels across the screen, the distance travelled determined by the force applied to the sensor within the initial 3-second window. In non-catch trials, the ball is seen to stop. In pseudo-random catch trials, the ball disappears early in its trajectory and participants are asked to estimate the final position of the ball using an estimation scale that appears on screen.

Supplementary Figure 6: Structural connectivity pipeline

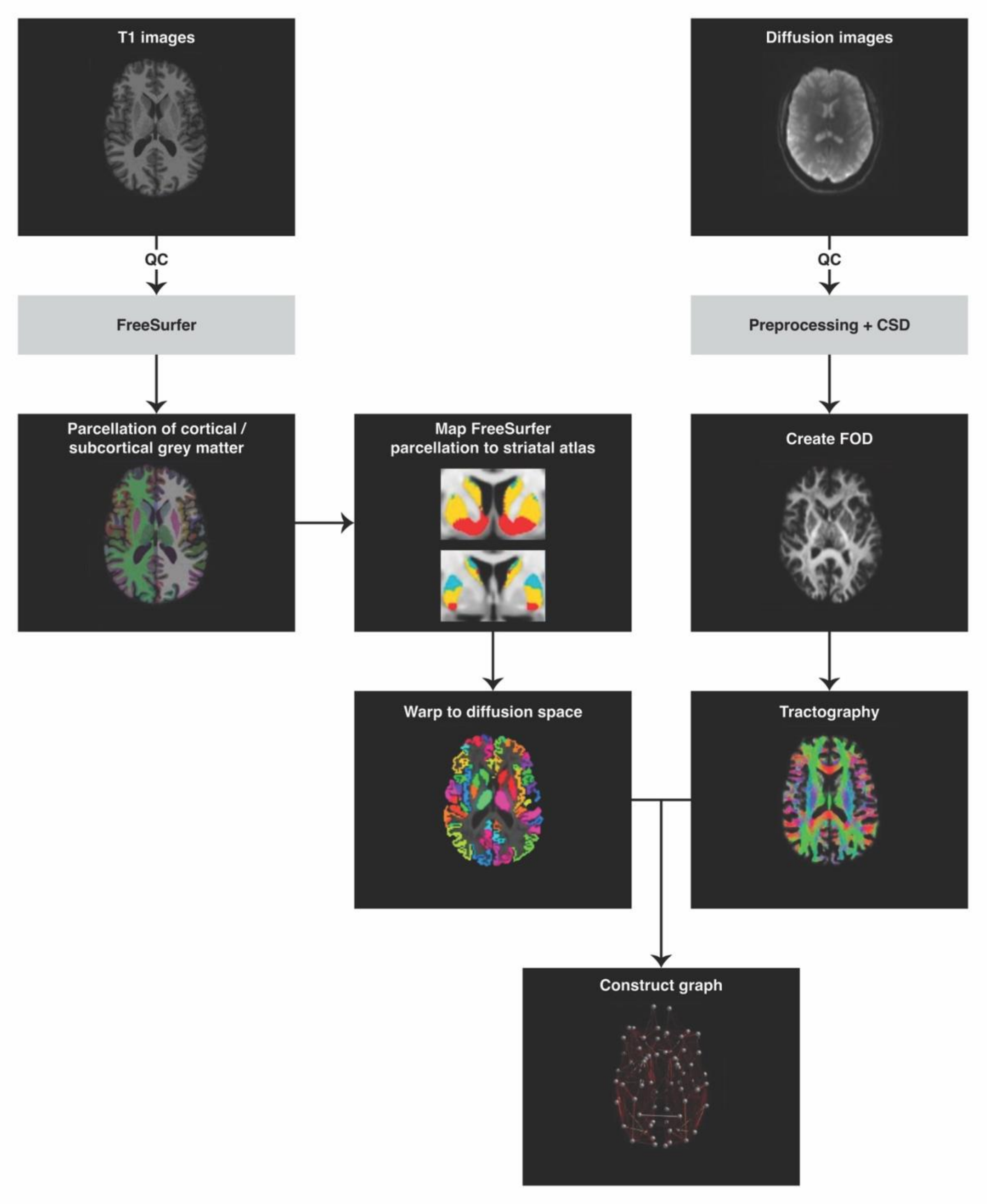

Panel showing structural connectivity pipeline.

Abbreviations: *CSD=Multi-shell Multi-tissue Constrained Spherical Deconvolution. QC=Quality Control. FOD=Fibre Orientation Distributions. Preprocessing – eddy current and motion correction, bias correction. Striatal atlas reprinted from FSL https://fsl.fmrib.ox.ac.uk/fsl/fslwiki/Atlases/striatumconn.*

References:

1. Penney JB, Vonsattel JP, MacDonald ME, et al. CAG repeat number governs the development rate of pathology in huntington's disease. *Ann Neurol* 1997; 41: 689–692.
2. Langbehn DR, Brinkman RR, Falush D, et al. A new model for prediction of the age of onset and penetrance for Huntington's disease based on CAG length. *Clin Genet* 2004; 65: 267–277.
3. Scahill RI, Zeun P, Osborne-Crowley K, et al. Biological and clinical characteristics of gene carriers far from predicted onset in the Huntington's disease Young Adult Study (HD-YAS): a cross-sectional analysis. *Lancet Neurol* 2020; 19: 502–512.
4. Unified Huntington's Disease Rating Scale: reliability and consistency. Huntington Study Group. *Mov Disord* 1996; 11: 136–42.
5. Shoulson I, Fahn S. Huntington disease: clinical care and evaluation. *Neurology* 1979; 29: 1–3.
6. Bland AR, Roiser JP, Mehta MA, et al. EMOTICOM: A Neuropsychological Test Battery to Evaluate Emotion, Motivation, Impulsivity, and Social Cognition. *Front Behav Neurosci* 2016; 10: 25.
7. Stroop JR. Studies of interference in serial verbal reactions. *J Exp Psychol* 1935; 18: 643–662.
8. Tabrizi SJ, Scahill RI, Owen G, et al. Predictors of phenotypic progression and disease onset in premanifest and early-stage Huntington's disease in the TRACK-HD study: analysis of 36-month observational data. *Lancet Neurol* 2013; 12: 637–49.
9. Hezemans FH, Wolpe N, Rowe JB. Apathy is associated with reduced precision of prior beliefs about action outcomes. *J Exp Psychol Gen* 2020; 149: 1767–1777.
10. Heath CJ, O'Callaghan C, Mason SL, et al. A Touchscreen Motivation Assessment Evaluated in Huntington's Disease Patients and R6/1 Model Mice. *Front Neurol* 2019; 10: 858.
11. Hezemans FH, Wolpe N, O'Callaghan C, et al. Noradrenergic deficits contribute to apathy in Parkinson's disease through the precision of expected outcomes. *PLoS Comput Biol* 2022; 18: e1010079.
12. Zung WW, Richards CB, Short MJ. Self-rating depression scale in an outpatient clinic. Further validation of the SDS. *Arch Gen Psychiatry* 1965; 13: 508–15.
13. Spielberger CD GRLRVPJG. *Manual for the State-Trait Anxiety Inventory*. Palo Alto, CA: Consulting Psychologists Press, Inc., 1983.
14. Patton JH, Stanford MS, Barratt ES. Factor structure of the Barratt impulsiveness scale. *J Clin Psychol* 1995; 51: 768–74.
15. Stout JC, Ready RE, Grace J, et al. Factor analysis of the frontal systems behavior scale (FrSBe). *Assessment* 2003; 10: 79–85.
16. Foa EB, Huppert JD, Leiberg S, et al. The Obsessive-Compulsive Inventory: development and validation of a short version. *Psychol Assess* 2002; 14: 485–96.
17. Ang Y-S, Lockwood P, Apps MAJ, et al. Distinct Subtypes of Apathy Revealed by the Apathy Motivation Index. *PLoS One* 2017; 12: e0169938.

18. Sockeel P, Dujardin K, Devos D, et al. The Lille apathy rating scale (LARS), a new instrument for detecting and quantifying apathy: validation in Parkinson's disease. *J Neurol Neurosurg Psychiatry* 2006; 77: 579–84.
19. Buysse DJ, Reynolds CF, Monk TH, et al. The Pittsburgh Sleep Quality Index: a new instrument for psychiatric practice and research. *Psychiatry Res* 1989; 28: 193–213.
20. Ware JE, Sherbourne CD. The MOS 36-item short-form health survey (SF-36). I. Conceptual framework and item selection. *Med Care* 1992; 30: 473–83.
21. Sled JG, Zijdenbos AP, Evans AC. A nonparametric method for automatic correction of intensity nonuniformity in MRI data. *IEEE Trans Med Imaging* 1998; 17: 87–97.
22. Freeborough PA, Fox NC, Kitney RI. Interactive algorithms for the segmentation and quantitation of 3-D MRI brain scans. *Comput Methods Programs Biomed* 1997; 53: 15–25.
23. Fox NC, Freeborough PA, Rossor MN. Visualisation and quantification of rates of atrophy in Alzheimer's disease. *Lancet* 1996; 348: 94–7.
24. Whitwell JL, Crum WR, Watt HC, et al. Normalization of cerebral volumes by use of intracranial volume: implications for longitudinal quantitative MR imaging. *AJNR Am J Neuroradiol* 2001; 22: 1483–9.
25. Ledig C, Heckemann RA, Hammers A, et al. Robust whole-brain segmentation: application to traumatic brain injury. *Med Image Anal* 2015; 21: 40–58.
26. Ashburner J, Friston KJ. Voxel-based morphometry--the methods. *Neuroimage* 2000; 11: 805–21.
27. Mansoor NM, Vanniyasingam T, Malone I, et al. Validating Automated Segmentation Tools in the Assessment of Caudate Atrophy in Huntington's Disease. *Front Neurol* 2021; 12: 616272.
28. Freeborough PA, Fox NC. Modeling brain deformations in Alzheimer disease by fluid registration of serial 3D MR images. *J Comput Assist Tomogr* 1998; 22: 838–43.
29. Tabrizi SJ, Scahill RI, Durr A, et al. Biological and clinical changes in premanifest and early stage Huntington's disease in the TRACK-HD study: the 12-month longitudinal analysis. *Lancet Neurol* 2011; 10: 31–42.
30. Jenkinson M, Bannister P, Brady M, et al. Improved optimization for the robust and accurate linear registration and motion correction of brain images. *Neuroimage* 2002; 17: 825–41.
31. Andersson JLR, Sotiropoulos SN. An integrated approach to correction for off-resonance effects and subject movement in diffusion MR imaging. *Neuroimage* 2016; 125: 1063–1078.
32. Graham MS, Drobnjak I, Zhang H. Realistic simulation of artefacts in diffusion MRI for validating post-processing correction techniques. *Neuroimage* 2016; 125: 1079–1094.
33. Zhang H, Schneider T, Wheeler-Kingshott CA, et al. NODDI: practical in vivo neurite orientation dispersion and density imaging of the human brain. *Neuroimage* 2012; 61: 1000–16.
34. Daducci A, Canales-Rodríguez EJ, Zhang H, et al. Accelerated Microstructure Imaging via Convex Optimization (AMICO) from diffusion MRI data. *Neuroimage* 2015; 105: 32–44.
35. Mori S, Oishi K, Jiang H, et al. Stereotaxic white matter atlas based on diffusion tensor imaging in an ICBM template. *Neuroimage* 2008; 40: 570–582.

36. Keihaninejad S, Zhang H, Ryan NS, et al. An unbiased longitudinal analysis framework for tracking white matter changes using diffusion tensor imaging with application to Alzheimer's disease. *Neuroimage* 2013; 72: 153–63.
37. Zhang J, Gregory S, Scahill RI, et al. In vivo characterization of white matter pathology in premanifest huntington's disease. *Ann Neurol* 2018; 84: 497–504.
38. Parker CS, Veale T, Bocchetta M, et al. Not all voxels are created equal: Reducing estimation bias in regional NODDI metrics using tissue-weighted means. *Neuroimage* 2021; 245: 118749.
39. Desikan RS, Ségonne F, Fischl B, et al. An automated labeling system for subdividing the human cerebral cortex on MRI scans into gyral based regions of interest. *Neuroimage* 2006; 31: 968–80.
40. Smith RE, Tournier J-D, Calamante F, et al. Anatomically-constrained tractography: improved diffusion MRI streamlines tractography through effective use of anatomical information. *Neuroimage* 2012; 62: 1924–38.
41. McColgan P, Seunarine KK, Razi A, et al. Selective vulnerability of Rich Club brain regions is an organizational principle of structural connectivity loss in Huntington's disease. *Brain* 2015; 138: 3327–44.
42. McColgan P, Seunarine KK, Gregory S, et al. Topological length of white matter connections predicts their rate of atrophy in premanifest Huntington's disease. *JCI Insight*; 2. Epub ahead of print 20 April 2017. DOI: 10.1172/jci.insight.92641.
43. Tziortzi AC, Haber SN, Searle GE, et al. Connectivity-based functional analysis of dopamine release in the striatum using diffusion-weighted MRI and positron emission tomography. *Cereb Cortex* 2014; 24: 1165–77.
44. Modat M, Ridgway GR, Taylor ZA, et al. Fast free-form deformation using graphics processing units. *Comput Methods Programs Biomed* 2010; 98: 278–84.
45. Jeurissen B, Tournier J-D, Dhollander T, et al. Multi-tissue constrained spherical deconvolution for improved analysis of multi-shell diffusion MRI data. *Neuroimage* 2014; 103: 411–426.
46. Smith RE, Tournier J-D, Calamante F, et al. SIFT2: Enabling dense quantitative assessment of brain white matter connectivity using streamlines tractography. *Neuroimage* 2015; 119: 338–51.
47. Rubinov M, Sporns O. Complex network measures of brain connectivity: uses and interpretations. *Neuroimage* 2010; 52: 1059–69.
48. Bullmore E, Sporns O. Complex brain networks: graph theoretical analysis of structural and functional systems. *Nat Rev Neurosci* 2009; 10: 186–98.
49. Baggio HC, Segura B, Junque C. Resting-state functional brain networks in Parkinson's disease. *CNS Neurosci Ther* 2015; 21: 793–801.
50. Weiskopf N, Suckling J, Williams G, et al. Quantitative multi-parameter mapping of R1, PD(*), MT, and R2(*) at 3T: a multi-center validation. *Front Neurosci* 2013; 7: 95.
51. Tabelow K, Balteau E, Ashburner J, et al. hMRI - A toolbox for quantitative MRI in neuroscience and clinical research. *Neuroimage* 2019; 194: 191–210.
52. Byrne LM, Rodrigues FB, Johnson EB, et al. Cerebrospinal fluid neurogranin and TREM2 in Huntington's disease. *Sci Rep* 2018; 8: 4260.
53. Thompson A, Schäfer J, Kuhn K, et al. Tandem mass tags: a novel quantification strategy for comparative analysis of complex protein mixtures by MS/MS. *Anal Chem* 2003; 75: 1895–904.

54. Tijms BM, Gobom J, Reus L, et al. Pathophysiological subtypes of Alzheimer's disease based on cerebrospinal fluid proteomics. *Brain* 2020; 143: 3776–3792.
55. Li J, Cai Z, Vaites LP, et al. Proteome-wide mapping of short-lived proteins in human cells. *Mol Cell* 2021; 81: 4722-4735.e5.
56. Batth TS, Francavilla C, Olsen J V. Off-line high-pH reversed-phase fractionation for in-depth phosphoproteomics. *J Proteome Res* 2014; 13: 6176–86.
57. UniProt Consortium. UniProt: a worldwide hub of protein knowledge. *Nucleic Acids Res* 2019; 47: D506–D515.
58. Tabrizi SJ, Langbehn DR, Leavitt BR, et al. Biological and clinical manifestations of Huntington's disease in the longitudinal TRACK-HD study: cross-sectional analysis of baseline data. *Lancet Neurol* 2009; 8: 791–801.
59. Winder JY, Roos RAC, Burgunder J-M, et al. Interrater Reliability of the Unified Huntington's Disease Rating Scale-Total Motor Score Certification. *Mov Disord Clin Pract* 2018; 5: 290–295.
60. Lawrence AD, Hodges JR, Rosser AE, et al. Evidence for specific cognitive deficits in preclinical Huntington's disease. *Brain* 1998; 121 (Pt 7): 1329–41.
61. Coull JT, Frith CD, Frackowiak RS, et al. A fronto-parietal network for rapid visual information processing: a PET study of sustained attention and working memory. *Neuropsychologia* 1996; 34: 1085–95.
62. Aron AR, Poldrack RA. Cortical and subcortical contributions to Stop signal response inhibition: role of the subthalamic nucleus. *J Neurosci* 2006; 26: 2424–33.
63. Goh AM, Wibawa P, Loi SM, et al. Huntington's disease: Neuropsychiatric manifestations of Huntington's disease. *Australas Psychiatry* 2018; 26: 366–375.
64. Pironti VA, Lai M-C, Müller U, et al. Neuroanatomical abnormalities and cognitive impairments are shared by adults with attention-deficit/hyperactivity disorder and their unaffected first-degree relatives. *Biol Psychiatry* 2014; 76: 639–47.
65. Aron AR, Dowson JH, Sahakian BJ, et al. Methylphenidate improves response inhibition in adults with attention-deficit/hyperactivity disorder. *Biol Psychiatry* 2003; 54: 1465–8.
